# Maternal Medication Use and Childhood Cancer in Offspring—Systematic Review and Considerations for Researchers

**DOI:** 10.1093/aje/kwab154

**Published:** 2021-05-21

**Authors:** Sarah Hjorth, Caroline H Hemmingsen, Justine Bénévent, Anne Broe, Anton Pottegaard, Lina S Mørch, Maarit K Leinonen, Susanne K Kjaer, Marie Hargreave, Hedvig Nordeng

**Keywords:** cancer, child, delayed effects, medications, pharmacoepidemiology, prenatal exposure

## Abstract

Cancer is an important cause of childhood mortality, yet the etiology is largely unknown. A combination of pre- and postnatal factors is thought to be implicated, including maternal medication use. We aimed to provide: 1) a systematic review of peer-reviewed publications on associations between maternal medication use and childhood cancer, with a focus on study design and methodology; and 2) suggestions for how to increase transparency, limit potential biases, and improve comparability in studies on maternal medication use and childhood cancer. We conducted a systematic search in the PubMed, Embase, Scopus, Cochrane, and Web of Science databases to June 8, 2020. Altogether, 112 studies were identified. The reviewed studies were heterogeneous in study design, exposure, and outcome classification. In 21 studies (19%), the outcome was any childhood cancer. Of the 91 papers that reported on specific types of cancer, 62% did not report the cancer classification system. The most frequently investigated medication groups were sex hormones (46 studies, excluding fertility medications), and antiinfectives (37 studies). Suggestions for strengthening future pharmacoepidemiologic studies on maternal medication use and childhood cancer relate to choice of cancer classification system, exposure windows, and methods for identification of, and control for, potential confounders.

## Abbreviations

ICCC: *International Classification of Childhood Cancers*
ICD: *International Classification of Diseases*

The worldwide incidence of childhood cancer is estimated at 140 per million person-years and is increasing (1–3). The etiology of childhood cancer is largely unknown but thought to be explained by both pre- and postnatal factors (4–6). The increasing incidence of childhood cancer could point to environmental risk factors that have changed over time (2–4).

The only fully established transplacental chemical carcinogen is diethylstilbestrol (7). The research on diethylstilbestrol sparked an interest in the investigation of maternal medication use and risk of childhood cancers (8). In recent years, some studies (9–12), but not all (13, 14), pointed to an association between maternal medication use and childhood cancer. A review of the literature before 1997 illustrated the heterogeneity in the literature, in particular with regard to the applied cancer classification (8). Suggestions for methods have been provided for pharmacoepidemiologic studies of medication-cancer associations in adults (15), but to our knowledge, guidance has not been provided for maternal medication–childhood cancer associations. Therefore, we aimed to provide: 1) a review of peer-reviewed publications on associations between maternal medication use and childhood cancer, with a focus on study design and methodology; and 2) suggestions on how to increase transparency, limit potential biases, and improve comparability in studies on maternal medication use and childhood cancer.

## METHODS

### Search strategy

We conducted a systematic search in PubMed (National Center for Biotechnology Information, Bethesda, Maryland), Embase (Elsevier BV, Amsterdam, the Netherlands), Scopus (Elsevier BV), Cochrane (John Wiley & Sons, Hoboken, NJ), and Web of Science (Core collection, The Thomson Corporation, Toronto, Canada) from database inception (1966 or earlier depending on database) to June 8, 2020, to address the following specific questions:

- Classification of childhood cancer: How was the outcome classified (as any cancer, or according to specific diagnoses; specified by the International Classification of Diseases (ICD), the International Classification of Childhood Cancers (ICCC), or other)?
- Exposure windows: What timing of maternal medication use was investigated (during pregnancy only, including periods before pregnancy, or during breastfeeding)?
- Study design: What study designs were used?
- Methods and statistics: What statistical and epidemiologic methods were used?
- Follow-up/age at case ascertainment: What was the maximum age at follow-up (cohort studies)/age at case ascertainment (case-control studies)?

####  

Reference lists of relevant reviews and included studies were screened to ensure complete coverage of the published literature. Our initial search used the search terms “child” AND “prenatal” AND “medication” AND “cancer,” including relevant synonyms. However, this search proved too narrow, as more studies were identified from reference lists than from the search itself. Many such studies identified via reference lists did not include the term “medication” in their titles or abstracts. The search was therefore repeated using only “child” AND “prenatal” AND “cancer,” and relevant synonyms. An example of search terms and search strategy for the PubMed database can be found in Web Table 1 (available at https://doi.org/10.1093/aje/kwab154).

####  

References were imported into the reference management program Endnote (16), where duplicates were removed. The remaining references were imported to Rayyan QCRI (17), a platform for management of systematic review data. Title and abstract screening, as well as full text screening were performed independently by 2 reviewers (S.H. and C.H.H.). Any disagreement was solved by a discussion among all authors.

### Inclusion criteria

Studies were considered eligible for inclusion if they fulfilled the following criteria for participants, exposures, comparators, outcomes, and study design: Participants were required to be children, defined as individuals under the age of 20 years. The exposure was restricted to maternal prepregnancy or pregnancy use of prescription or over-the-counter medication, as identified in prescription data or from self-reported data. To maintain the study focus on therapeutic medications, studies on supplements (vitamins/minerals) and studies on use of illegal substances were not included. Studies that classified exposure as “any medication” were also excluded. Comparators were children born to mothers who did not use the specified medications. This included children born to healthy mothers (population comparators), children born to mothers with illnesses but not receiving treatment (disease comparators), and children born to mothers who used specified medications other than the medication of interest for the study (active comparators). The outcome was childhood cancer. Studies that used children with other types of cancer as controls were excluded. Randomized controlled trials, cohort studies, and case-control studies were eligible for inclusion. Papers that were not original studies (e.g., reviews and editorials), studies without a comparison group, cross-sectional studies, ecological studies, and animal studies were excluded, as were conference abstracts, study protocols, and pilot studies. No restrictions were applied as to study date or setting, but for resource reasons, the search was limited to peer-reviewed publications in English, French, or one of the Scandinavian languages.

### Data extraction

Data items extracted from the included studies were decided a priori as follows: study design, setting, sample size, number of exposed, number of cases, age at end of follow-up/case ascertainment, exposure and outcome classification, statistical analysis, and adjustment variables. Data was extracted by S.H. and J.B.

No systematic tool like GRADE (18) or ROBINS-I (19) was used to assess risk of bias in the individual studies. Instead, all eligible studies were assessed according to the prespecified questions mentioned above, and studies were discussed in the author group to identify suggestions for future research.

Data was grouped by medication exposure according to indication for use. Given the study aims, no synthesis of study findings was planned.

### Post hoc sensitivity analyses

We performed 2 sensitivity analyses post hoc to assess the robustness of the findings. The first was restricted to the most recent studies, published between 2011 and 2020, as methodological developments over the years might have had an impact on the quality of the included studies. The second was a restriction to studies where childhood cancer was the main outcome, given that analyses of main and secondary outcomes might differ systematically.

**Figure 1:**
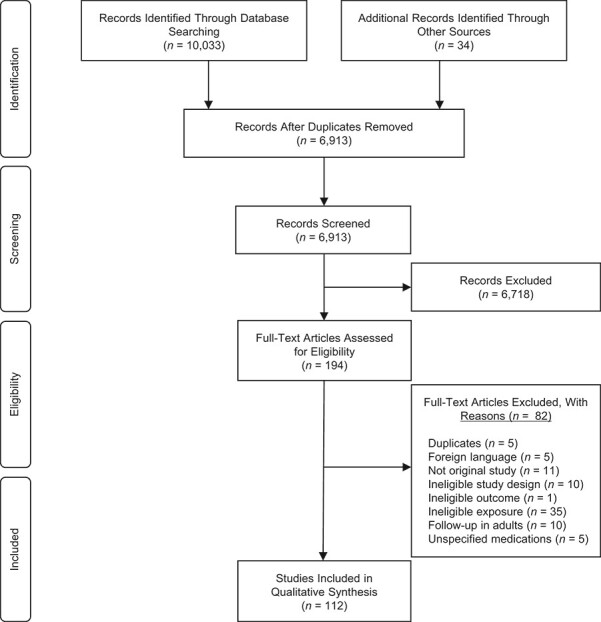
Flowchart for selection of studies relating to the associations between maternal medication use and childhood cancer, according to Preferred Reporting Items for Systematic Reviews and Meta-Analyses (125).

## RESULTS

The literature search yielded 10,033 studies. After removal of duplicate records, 6,879 studies were left for title and abstract screening. Of these, 160 were relevant for full-text assessment, and 78 were eligible for inclusion (7, 9–14, 20–90). An additional 34 studies were identified from reference lists of included studies and relevant reviews (91–124). See Figure 1 for flowchart showing selection according to Preferred Reporting Items for Systematic Reviews and Meta-Analyses (125).

Data was extracted from 112 studies performed between 1958 and 2020. Studies originated from Europe (50 studies), North America (48 studies), Asia (6 studies), South America (4 studies), and Australia (2 studies). In addition, there were 2 transcontinental studies (Table 1).

### Classification of childhood cancer

In 21 studies (19%), the outcome was a composite endpoint of “any childhood cancer” (Table 1). An additional 13 studies (12%) reported results for any childhood cancer, in addition to specific types of cancer. Of the 91 papers that reported on specific types of cancer, 62% did not report the cancer classification system. In the remaining papers, the most commonly reported classification system was the ICD. Some studies had their classification system labeled as “other,” for instance, because they used more detailed classifications by disease staging (74). Several studies (32%) reported more than one group or specific type of cancer (range 1–16). The most commonly reported main diagnostic groups were leukemias (25 studies) and central nervous system tumors (14 studies). The most commonly investigated subgroups of childhood cancer were acute lymphoblastic leukemias (26 studies), acute myeloid leukemias (15 studies), and neuroblastomas (14 studies).

**Table 1:** Summary Characteristics of 112 Included Studies on Associations Between Maternal Medication Use and Childhood Cancer, Worldwide, 1958–2020

**Study Characteristic**	**No. of Studies (*n* = 112)**	**%**
Year of publication		
<1971	1	1
1971–1980	6	5
1981–1990	21	19
1991–2000	19	17
2001–2010	27	24
2011–2020	38	34
Continent where study was conducted		
Africa	0	0
Asia	6	5
Australia	2	2
Europe	50	45
North America	48	43
South America	4	4
Transcontinental	2	2
No. of medication groups investigated		
1	67	60
2	11	10
3	7	6
≥4	27	23
Cancer classification system		
Any cancer	21	19
International Classification of Childhood Cancers	10	9
International Classification of Diseases	17	15
International Classification of Diseases for Oncology	4	4
Other	4	4
Not specified	56	50
Study design^a^		
Case-control	81	72
Case-cohort	7	6
Cohort	24	21
Randomized controlled trial	0	0
Exposure ascertainment^b^		
Maternal retrospective self-report	70	63
Maternal prospective self-report	4	4
Routinely collected health data	43	38
Statistical analysis		
Cox regression	17	15
Descriptive analysis	12	11
Logistic regression	65	58
Poisson regression	2	2
Other	16	14
Maximum follow-up		
<5 years	10	9
5–9 years	11	10
10–14 years	51	46
≥15 years	33	29
Not specified	7	6
Comparator group^c^		
Population comparator	101	90
Disease comparator	8	7
Active comparator	2	2
Other^d^	3	3
Sample size		
<100	2	2
100–499	34	30
500–999	23	21
1,000–9,999	29	26
≥10,000	24	21
No. of cases^e^		
<100	32	29
100–499	48	43
500–999	17	15
≥1,000	16	14
No. of exposed^e^		
<100	68	61
100–499	42	38
500–999	4	4
≥1,000	21	19
Not stated	9	8
Adjustment or matching strategy in main analysis		
No adjustment or matching	8	7
Only confounders	82	73
At least 1 intermediate factor	19	17
Not specified	3	3

^a^ The numbers do not add up because one study presented results from 2 countries. In one country, the design was case-cohort, and in the other, case-control.

^b^ The numbers do not add up because 5 studies used 2 sources of exposure ascertainment.

^c^ The numbers do not add up because 2 studies had more than 1 type of comparators.

^d^ In one study the comparator group included cousins of exposed, in another study it included children of unexposed mothers and exposed fathers, and in the third study it included children of women who used the medication prior to pregnancy only.

^e^ Numbers reported for the main analysis. If main exposure or outcome were not stated, all exposures and outcomes were considered equal, and the study could end up in more than one category.

### Exposure

Several studies (40%) investigated more than 1 medication group (range 1–13) (Table 1). The most frequently investigated medication groups were sex hormones (46 studies, excluding fertility medications), antiinfectives (37 studies), and fertility medications (30 studies) (Web Table 2).

In 15% of studies, more than 1 exposure window was investigated. The majority (56%), investigated maternal medication use anytime during pregnancy, whereas only 6% investigated use in specific trimesters of pregnancy. Many studies (37%) investigated use prior to pregnancy, mainly use of fertility medications or sex hormones. Although most studies distinguished between use before, during, and after pregnancy, 14% of studies investigated an exposure window including more than one peripregnancy period (e.g., prepregnancy and pregnancy, or pregnancy and breastfeeding). Analyses by dose or duration were available in 10 studies (9%).

Exposure was ascertained by maternal retrospective self-report in 70 studies (63%), from routinely collected health data in 43 studies (38%), and from maternal prospective self-report in 4 studies (4%). Five studies (4%) used 2 sources of exposure ascertainment. A total of 27 studies (24%) accounted for potential exposure misclassification; 24 of these had ascertained exposure by maternal retrospective recall.

### Study design

The majority of studies were case-control studies (81 studies, 72%), 21% were cohort studies, and the remainder were case-cohort studies (Table 1). Two studies had less than 100 participants, and 21% of studies had more than 10,000 participants. In contrast to the high number of participants, 61% of the studies had less than 100 exposed, and 29% of studies had less than 100 cases in their main analysis. In 9 studies, there were either no cases, or no exposed cases or controls, for the main analysis. Almost all studies used population comparators (90%), but disease comparators were used in 8 studies, and active comparators were used in 2 studies. Some studies used more than 1 type of comparator.

### Statistical and epidemiologic methods

The most commonly used statistical analysis was logistic regression (58% of studies). Cox regression was used in 15% of studies, and 2 studies used Poisson regression. Purely descriptive analysis was used in 11% of the studies, mainly when the sample size was limited, or when cancer was not the main outcome of the study. The studies using other statistical methods (14%) were predominantly studies performed in the 1970s and 1980s. An example of a statistical method used in these studies is the Mantel-Haenszel method for stratified analysis (44, 54, 103). Whereas 73% of studies included matching or adjustment for 1 or more potential confounding factors, 7% had no adjustment, and 17% adjusted for at least 1 potential intermediate factor in their main analysis (Table 1). The most frequently included intermediate factors were gestational age at birth (11 studies) and birth weight (10 studies).

### Follow-up/age at case ascertainment

The mean upper limit of follow-up/age at case ascertainment for the children was 14 years. In 35% of studies, the age range was 0–14 years, and in 18% of studies, the age range was 0–19 years. The remaining studies used other age ranges, either determined by the peak incidence of the type of cancer investigated (e.g., less than 5 years of age for hepatoblastoma (104)), or by data availability (e.g., less than 9 years in a study using data from an existing birth cohort (82)).

### Post hoc sensitivity analyses

In the first sensitivity analysis of 38 studies published between 2011 and 2020, “any childhood cancer” was used as the outcome by a larger proportion of studies than in the studies published before 2011 (26% compared with 15%), and more studies on specific cancer types reported on the classification system used (14 of 28 studies on specific cancer types, 50% compared with 33% in the studies published before 2011). A lower proportion of studies ascertained exposure by maternal retrospective report (39% compared with 74%), but a larger proportion adjusted for at least 1 intermediate factor in a main analysis (29% compared with 11%).

The second sensitivity analysis excluded 7 studies that did not have childhood cancer as their main outcome. The results were largely similar to the main analysis, except that a lower proportion of studies (14 of 105 studies, 13%) had investigated childhood cancer as a composite outcome.

## DISCUSSION

### Main findings

In this review of 112 studies on maternal use of medication before or during pregnancy and childhood cancer in offspring, 19% used “any cancer” as the outcome. A majority of the studies that investigated specific types of cancer did not report the cancer classification system (62%). In most studies (56%), the exposure window was anytime during pregnancy, but 15% of studies investigated more than one exposure window. A majority of studies were case-control studies (72%). Most studies (73%) accounted for potential confounding by matching and/or adjustment, but 17% of studies adjusted for at least 1 intermediate factor in a main analysis. The mean upper limit of follow-up/age at case ascertainment was 14 years.

### Limitations

Of 112 included studies, 34 were identified from reference lists. Half of these investigated fertility medications, whereas the other half did not have any pattern of common characteristics. This could indicate that the search terms included in the literature search were not exhaustive for fertility medications. Because it takes some time from when a study is published to when it can be cited, we might have incomplete coverage of the literature from late 2019 and early 2020. In addition, we had to exclude 5 studies due to language restrictions. For these studies, we have not been able to assess eligibility in a full-text reading, and thus we do not know whether they fulfilled the inclusion criteria for the present review. Authors for whom English is not their first language are more likely to publish studies with negative findings in local, non-English-language journals (126). It is not known whether methods or reporting differ systematically between studies published in English and studies published in local journals.

### Considerations for future research

#### Reporting of cancer types according to the ICCC when possible.

It has been argued that “any cancer” should not be used as an outcome in studies of medication-cancer associations in adults (15). In brief, this is because cancer is a heterogeneous disease, and no known carcinogens increase the risk of every type of cancer (15). This also applies to childhood cancer, arguing for investigations of specific types of cancer when possible. Although limited sample sizes can render a detailed outcome classification impracticable, it should be noted that the grouping of outcomes might in fact reduce study precision. This seemingly counterintuitive claim stems from the fact that investigating several cancer types in one group will introduce heterogeneity leading to increased variance and therefore wider confidence intervals (127). However, for new and rarely used treatments (e.g., biologicals), studies with any cancer as the outcome might be the only option, and might still provide important reassurance or serve to flag initial safety signals. Yet, even if the risk estimate for overall cancer is not increased, this does not rule out the possibility that the studied medication increases the risk of a specific type of cancer. Therefore, results indicating null associations from studies using any cancer as the outcome should be reported and interpreted with caution. On the other hand, if studies using imprecise outcomes do identify a signal, this could warrant further investigation. To prioritize among multiple signals for further investigation, it can be useful to employ methods such as empirical Bayes shrinkage, which adjusts observed estimates of association for random variation and is thought to reduce the number of false positive findings (128). In studies with only a few exposed cases, it might be beneficial to apply a lesson learned from teratology (129, 130) and report on any patterns of specific cancer types, even if statistical analysis is feasible only for a combination of all cancers. It was the presence of a specific type of cancer that first suggested the transplacental carcinogenicity of diethylstilbestrol (7), just as the specific patterns of syndromes or malformations flagged the potential teratogenicity of thalidomide and valproic acid (129).

For studies that have the statistical power to report risk estimates for specific types of childhood cancer, the next question is what classification scheme to use. To facilitate pooling of data in meta-analyses, a standardization of the outcome classification would be helpful (8). The need for standardization led to the development of the ICCC in 1987 (131). Childhood cancers differ from cancers in adults by being embryonal in type and arising in organ systems that do not map onto the traditional sites that are used in classification of adult cancers (131). Therefore, ICCC is based primarily on the type and behavior of the cancer cells, with some site-based groupings to facilitate comparisons with ICD. In addition, the most common childhood cancers have individual codes (131). This makes the ICCC preferable to ICD for classification of childhood cancers. The International Classification of Diseases for Oncology, while not adapted specifically to childhood cancers, is still preferable to the ICD, because it takes morphology into account. From a pharmacological point of view, it is plausible that morphologically similar cancers will react to medication exposures in a similar fashion, whereas cancers of the same site might not (15). Hence, researchers should consider using the ICCC classification system when possible. The International Classification of Diseases for Oncology, recommended for use in cancer registries, could be an alternative in situations where mapping to the ICCC is not feasible.

#### Biological plausibility should guide the exposure definition and exposure windows.

Both preclinical findings (e.g., from in vitro or animal studies) and signals from previous human studies could be helpful to inform the exposure definition. If, for instance, the hypothesis is that hormonal disruption from exposure to oral contraceptives plays a causal role in the development of childhood nonlymphoid leukemias (9), it might be insufficient to study oral contraceptives as a group. Most oral contraceptives contain an estrogen analog and progestin, whereas others contain progestin only. Findings would then be attenuated for “any oral contraceptives,” if estrogen was the causally important substance. Further, analyses by dose or duration of exposure are helpful when feasible, given that there might be threshold levels for effect (15).

Once the exposure has been defined, the relevant exposure window should be chosen. As opposed to teratology, where the relevant exposure window for most malformations is the first trimester (129), relevant exposure window(s) for childhood cancers are largely unknown. One proposed relevant exposure window is immediately after fertilization, when the epigenome is thought to be highly sensitive to environmental factors (132). In the present review, different approaches were seen across studies, mainly ranging from a year before conception until the end of pregnancy. One study considered maternal exposure when the mother was a fetus (84). Seeing that the ovaries and egg cells for future offspring are formed in fetal life (133), it is possible that medications taken throughout the life course could affect the egg cells. Some studies investigated maternal medication use during pregnancy and/or breastfeeding (30, 63, 66, 89, 100). In studies where the exposure window extends beyond the prenatal period, the outcome can happen during the exposure window, thus potentially introducing immortal time bias (134). Immortal time bias can be avoided, for example, by moving the start of follow-up or case ascertainment to the end of the exposure window (134). The use of study design diagrams, as advocated by Schneeweiss et al. (135), could also help to clarify the timing of eligibility, exposure, covariate, and outcome assessment in the study. In Web Figure 1, we provide an example of a study design diagram. Because the relevant exposure window is uncertain, it could be beneficial to investigate several exposure definitions within one study (i.e., ever before pregnancy, 1 year before pregnancy, during pregnancy, during the first trimester, etc.). Another argument for the use of several different exposure definitions is specific to registry- or claims-based studies. In these studies, exposure in a given time window, such as the first trimester, is not driven solely by prescriptions filled in the time window. Prescriptions filled immediately before the time window might also contribute, if the dispensed medications covered part of the time window (136).

Regardless of the data source, authors should consider the risk of exposure misclassification. With prospective exposure ascertainment, the misclassification is often (but not always) nondifferential, whereas this cannot be assumed for retrospective maternal recall (137). Probabilistic sensitivity analysis is a useful tool to assess the potential impact of misclassification (138).

#### Use the new developments in confounder assessment and control.

Most studies in the present review addressed potential confounding through matching, adjustment, or both. Some, mainly newer, studies adjusted for potential intermediate factors such as gestational age and birth weight. In the best case scenario, adjusting for intermediate factors can preclude an estimation of the total effect of the exposure (139). However, it might introduce collider-stratification bias from unmeasured variables associated with both the intermediate factor and the outcome (139). Use of directed acyclic graphs could help ensure that adjustments are not made for intermediate factors (140). In Web Figure 2, we present an example of a directed acyclic graph. In many studies, the limited sample size can pose additional challenges for confounder adjustment. To avoid overfitting the models, serial change-in-estimate approaches for variable selection (141) or confounder summary scores can be used (142). Propensity score methods may be used if the exposure is more prevalent than the disease, because propensity scores are constructed from regression models with exposure as the dependent variable and covariates as independent variables (142).

Another method to estimate confounding in etiological epidemiology is by introducing a negative control (142, 143). A negative exposure control that has been suggested in perinatal pharmacoepidemiology is maternal medication use before pregnancy (142). However, as stated above, it cannot be ruled out that maternal prepregnancy exposure can affect the risk of childhood cancers, and so other negative controls should probably be preferred. Maternal medication use after birth can be used as a negative control in noncommunicable diseases, especially if maternal medication use is assessed when the breastfeeding has ceased. Paternal medication use while the mother is pregnant has been proposed to assess confounding by unmeasured genetic factors, or other unknown factors associated with medication use (144). If the main concern is confounding by the underlying maternal illness, the choice of disease or active comparators could be considered.

A majority of the studies in the present review used population comparators. This is a good choice in situations where the indication for medication use is not thought to influence cancer risk (e.g., contraceptives). However, if the underlying maternal disease has been linked to an increased risk of childhood cancer (e.g., autoimmune disease, human immunodeficiency virus infection (145)), use of population comparators might be inadequate. Here a disease comparator could instead be used, comparing illness treated with medications to the same illness managed without medication. An active comparator (i.e., comparing different substances used for the same indication, or comparing mono- and polytherapy) should be considered if a suitable comparator exists (15). Sibling comparators are useful primarily if the main source of confounding is thought to be inherited genetic risk (142). Provided that life-course maternal exposure to medications can affect risk of childhood cancers, carryover effects between siblings should be considered (146).

Compared with the choice of epidemiologic methods, the choice of statistical methods is less important. That is because childhood cancer, in particular specific types of cancer, occurs so rarely that estimates of rate ratios and risk ratios will be virtually identical (147).

## CONCLUSION

Studying associations between maternal medication use and childhood cancer is methodologically challenging. This systematic literature review showed that such studies are largely heterogeneous in their study design, exposure, and outcome classification. To improve the transparency, limit potential biases, and improve comparability of future studies, we propose 3 points of consideration bridging the fields of prenatal pharmacoepidemiology and cancer epidemiology. The points include: 1) investigating specific types of childhood cancer according to the ICCC classification when possible, or as a minimum stating the classification system used; 2) carefully considering relevant exposure windows, including whether several exposure windows should be investigated; and 3) using appropriate methods for identification of potential covariates (i.e., directed acyclic graphs) and control of confounding (e.g., disease comparators, active comparators, negative controls).

## Supplementary Material

Web_Material_kwab154
