# Supplementary material for: Maternal Medication Use and Childhood Cancer in Offspring—Systematic Review and Considerations for Researchers
Source: Am J Epidemiol. 2021 May 21;190(11):2487–99. doi: 10.1093/aje/kwab154 (PMC8561129; doi:10.1093/aje/kwab154)
Supplement: Web_Material_kwab154 [file web_material_kwab154.pdf]

Web Material

Maternal Medication Use and Childhood Cancer in Offspring – Systematic Review and Considerations for Researchers

Sarah Hjorth, Caroline H. Hemmingsen, Justine Bénévent, Anne Broe, Anton Pottegaard, Lina S. Mørch, Maarit K. Leinonen, Susanne K. Kjaer, Marie Hargreave*, and Hedvig Nordeng*

*Shared last authorship

Table of Contents

Web Table 1. Example of Search Terms and Search Strategy for the PubMed Database.

Web Table 2. Characteristics of the Included Studies by Medication Group.

Web Figure 1. Example of Study Design Diagram.

Web Figure 2. Example of Directed Acyclic Graph.

Web Table 1. Example of Search Terms and Search Strategy for the PubMed Database

**Patient:** Children	**Exposure:** Prenatal exposure	**Outcome:** Cancer
	**MeSH terms**	
Child<br>Child, preschool<br>Adolescent<br>Infant	Prenatal Exposure Delayed Effects<br>Maternal Exposure/*adverse effects<br>Pregnancy Complications/*drug therapy<br>Fetus/*drug effects<br>Maternal-Fetal Exchange	Neoplasms
	**All fields**	
Child<br>Childhood	Prenatal exposure*<br>Maternal exposure*<br>Pregnancy exposure*<br>Fetal exposure*<br>Foetal exposure*<br>In utero exposure*	Neoplasm*<br>Cancer*<br>Tumor*<br>Tumour*<br>Malignanc*<br>Leukemia*<br>Leukaemia*<br>Lymphoma*<br>Astrocytoma*<br>Neuroblastoma*<br>Nephroblastoma*<br>Rhabdomyosarcoma*

Search string:

((((((("prenatal exposure") OR "maternal exposure") OR "pregnancy exposure") OR "fetal exposure") OR "foetal exposure") OR "in utero exposure")) OR ((((((("Prenatal Exposure Delayed Effects"[Mesh]) OR "Maternal Exposure/adverse effects"[Mesh]) OR "Pregnancy Complications/drug therapy"[Mesh])) OR "Fetus/drug effects"[Mesh]) OR "Maternal-Fetal Exchange"[Mesh]))) AND ((((("Child"[Mesh] OR "Child, Preschool"[Mesh]) OR "Infant"[Mesh]) OR "Adolescent"[Mesh])) OR ((child) OR childhood))) AND (((((((((((((neoplasm*) OR cancer*) OR tumor*) OR tumour*) OR malignanc*) OR leukemia*) OR leukaemia*) OR lymphoma*) OR astrocytoma*) OR neuroblastoma*) OR nephroblastoma*) OR rhabdomyosarcoma*)) OR "Neoplasms"[Mesh]))

Web Table 2. Characteristics of the Included Studies by Medication Group

Reference	Exposure	Outcome	Materials and methods	
First author, year	Medication/*exposed*<br>*Ascertainment*<br>Exposure window: during pregnancy, unless otherwise stated	Classification system, type of cancer/*cases*	Design/*sample size*<br>*Study characteristics*<br>Statistical analysis	Method to account for confounding in the main analysis
ANALGESICS				
Bonaventure, 2015 (1)	Other analgesics and antipyretics (ATC group N02B)/*343*<br>*Routinely collected health data*	Classification: ICD<br>ALL/*725*, AML/*128*, NHL/*83*, HL/*31*, astrocytoma/*100*, medulloblastoma/*48*, rhabdomyosarcoma/*54*, Ewings sarcoma/*20*, neuroblastoma/*78*, nephroblastoma/*60*	Case-control/*4122*<br>*UK, UKCCS, mean age 5.8 (max age 15 years)*<br>Unconditional logistic regression	Matching on sex, month and year of birth, and region of residence. Adjustment for year of birth
Bunin, 1994 (2)	Prescription analgesics/*21*<br>*Maternal retrospective report*	Classification system not stated<br>Astrocytoma/*155*<br>PNET/*166*	Case-control/*332*<br>*US and Canada, Children's Cancer Group, ages 0-5 years*<br>Conditional logistic regression	Matching on telephone area code, year of birth and ethnicity. Adjustment for income (only for astrocytoma)
Carozza, 1995 (3)	Lidocaine/*1*, Propoxyphene HCL/*15*, Propranolol/*3*, Tetracaine/*1*<br>*Maternal retrospective report*	Classification: Histological Typing of Tumors of the Central Nervous System. International Histological Classification of Tumors<br>Any brain tumors/*361*	Case-control/*1444*<br>*US, SEER, upper age limit 18 years*<br>Conditional logistic regression, for main exposure (any n-nitrosatable drugs), descriptive statistics for medications stated here	Matching on age, sex and maternal ethnicity
Cook, 2004 (4)	Acetaminophen/*327*, Aspirin/*24*, Codeine/*28*, Ibuprofen/*28*<br>Each of the above during pregnancy or breastfeeding<br>*Maternal retrospective report*	Classification system not stated<br>Neuroblastoma/*504*	Case-control/*1008*<br>*US and Canada, Children's Cancer Group, upper age limit 18 years*<br>Conditional logistic regression	Matching on age. Adjustment for maternal age, education, and ethnicity
Couto, 2015 (5)	Acetaminophen/*83*, Dipyrone/*226*, Acetaminophen and dipyrone/*41*, Acetyl salicylic acid/*25*<br>*Maternal retrospective report, uses controls with other life-threatening illnesses to reduce recall bias*	Classification system not stated<br>ALL/*176*, AML/*55*, acute leukemia with rearrangements in the MLL gene/*43*	Case-control/*642*<br>*Brazil, ages 0-2 years*<br>Unconditional logistic regression	Matching on region. Adjustment for ethnicity, maternal age, education, pesticide exposure, and

Reference	Exposure	Outcome	Materials and methods	
First author, year	Medication/*exposed*<br>*Ascertainment*<br>Exposure window: during pregnancy, unless otherwise stated	Classification system, type of cancer/*cases*	Design/*sample size*<br>*Study characteristics*<br>Statistical analysis	Method to account for confounding in the main analysis
				hormone use during pregnancy, and child birth weight
Gilman, 1989 (6)	Analgesics and antipyretics/*444*<br>*Maternal retrospective report and medical records. Children are considered exposed if medication use is present in either source*	Classification system not stated<br>Any cancer/*8059*	Case-control/*16,118*<br>*UK, OSCC, age not specified, only that cases are childhood deaths from cancer*<br>Conditional logistic regression	Matching on date of birth, sex and district. Adjustment for abdominal x-rays in pregnancy, birth order among live born siblings, maternal age, and socioeconomic position
Grufferman, 1982 (7)	Aspirin/*67*, Analgesics and cold medications/*38*<br>Each of the above 1 year prior to pregnancy or during pregnancy<br>*Maternal retrospective report*	Classification system not stated<br>Rhabdomyosarcoma/*33*	Case-control/*132*<br>*US ages 0-14 years*<br>"Unmatched methods", not specified further	Matching on age, sex, and ethnicity
Hartley, 1988 (8)	Analgesics/*not reported*<br>*Maternal retrospective report, validated in prescription data*	Classification system not stated<br>Bone or soft tissue sarcomas/*73*	Case-control/*219*<br>*UK, IRESCC, ages 0-15 years*<br>Mantel-Haenszel, 95% CI by Cornfield's formula	Matching. Matching factors not reported, but they refer to a paper where methods are described (age and sex)
Heck, 2015 (9)	Prescription pain medication/*18*<br>*Maternal retrospective report*	Classification system not stated<br>Sporadic retinoblastoma, unilateral/*187*, Sporadic retinoblastoma, bilateral/*95*	Case-control/*426*<br>*US/Canada, ages 0-15 years*<br>Unconditional logistic regression	Matching on year of birth. Adjustment for maternal ethnicity, education, income, age, and smoking in pregnancy
Kelty & Hulse, 2017 (10)	Opioid maintenance treatment (methadone, buprenorphine, naltrexone)/*387*<br>*Routinely collected health data*	Classification: ICD<br>Any cancer/*4*	Cohort/*774*<br>*Australia, ages 0-11 years*<br>Descriptive statistics	No adjustment
Kramer, 1987 (11)	Prescription analgesics/*not reported*<br>*Maternal retrospective report, validated in medical records for a subsample. Validity compared between cases and controls*	Classification system not stated<br>Neuroblastoma/*104*	Case-control/*205*<br>*US, median age 1 year*<br>90% CI calculated by Miettinen's technique	Matching on telephone area code, ethnicity, and year of birth
Kuijten, 1990 (12)	General anesthesia/*not reported*<br>*Maternal retrospective report*	Classification system not stated<br>Astrocytoma/*163*	Case-control/*326*<br>*US, ages 0-14 years*	Matching on telephone area code, ethnicity, and age

Reference	Exposure	Outcome	Materials and methods	
First author, year	Medication/*exposed*<br>*Ascertainment*<br>Exposure window: during pregnancy, unless otherwise stated	Classification system, type of cancer/*cases*	Design/*sample size*<br>*Study characteristics*<br>Statistical analysis	Method to account for confounding in the main analysis
			Conditional logistic regression	
McCredie, 1994 (13)	Non-prescription analgesics in the month before or during pregnancy/*14*<br>*Maternal retrospective report*	Classification: ICD<br>Brain or cranial nerves tumors/*82*	Case-control/*246*<br>*Australia, ages 0-14 years*<br>Conditional logistic regression	Matching on sex and age. Adjustment for parental education
McKinney, 1987 (14)	Analgesics, Narcotic analgesics/*not reported*<br>*Maternal retrospective report and medical records, findings from the two data sources are reported separately*	Classification system not stated<br>Main outcome:<br>Leukemias or lymphomas/*234*<br>Secondary outcomes:<br>Leukemias/*171*<br>Only reported separately for significant associations	Case-control/*702*<br>*UK, IRESCC, ages 0-15 years*<br>Mantel-Haenszel	Matching on age and sex
McKinney, 1999 (15)	Analgesics/*36*, General anesthetics/*6*<br>*Routinely collected health data*	Classification: ICCC<br>Main outcome: Leukemia/*144*<br>Secondary outcome: ALL/*124*, lymphomas/*45*, CNS tumors/*75*, other solid tumors/*126*	Case-control/*415*<br>*Scotland, ages 0-14 years*<br>Conditional logistic regression	Matching on age, area of residence, and sex
Ognjanovic, 2011 (16)	Aspirin/*59 before, 21 after knowledge of pregnancy*<br>Non-aspirin NSAIDs/*193 before, 58 after knowledge of pregnancy*<br>Acetaminophen/*377 before, 407 after knowledge of pregnancy*<br>Also by any use or regular use<br>*Maternal retrospective report*	Classification system not stated<br>Infant ALL/*262*, infant AML/*172*, also analyzed by MLL gene translocation status	Case-control/*582*<br>*US, Children's Oncology Group, ages 0-1 years*<br>Unconditional logistic regression	Matching on year of birth and region of residence. Adjustment for maternal age, ethnicity, alcohol use during pregnancy, income, and child year of birth
Pombo de Oliveira, 2006 (17)	Dipyrone/*352*, Others (Acetaminophen, aspirin, hyoscine, codeine)/*161*<br>Each of the above 3 months before pregnancy, during pregnancy, or during breastfeeding<br>*Maternal retrospective report*	Classification system not stated<br>Infant acute leukemias/*202*	Case-control/*642*<br>*Brazil, ages 0-21 months*<br>Unconditional logistic regression	Matching on age and region. Adjustment for region, sex, birth weight, maternal age, and income
Preston-Martin, 1982 (18)	General anesthesia/*6 discordant pairs*<br>*Maternal retrospective report*	Classification system not stated<br>Tumor of the brain or cranial meninges, excluding tumors in infants/*209*	Case-control/*418*<br>*US, ages 0-25 years*<br>Conditional logistic regression	Matching on sex, ethnicity, birth year, and socioeconomic status

Reference	Exposure	Outcome	Materials and methods	
First author, year	Medication/*exposed*<br>*Ascertainment*<br>Exposure window: during pregnancy, unless otherwise stated	Classification system, type of cancer/*cases*	Design/*sample size*<br>*Study characteristics*<br>Statistical analysis	Method to account for confounding in the main analysis
Ross, 2003 (19)	Acetaminophen with codeine/*19*<br>*Routinely collected health data*	Classification system not stated<br>IL/*243*, ALL/*157*, AML/*77*	Case-control/*636*<br>*US, ages 0-18 months*<br>Conditional logistic regression	Matching on birth year and telephone area. Adjustment for maternal age, education and income
Salonen, 1976 (20)	Salicylates/*4*, Pyrazol derivatives/*7*, Aniline derivatives/*4*,<br>Euphoriant analgesics/*16*,<br>Medication for migraine and nausea/*23*<br>*Routinely collected health data*	Classification: ICD<br>Main outcomes: Leukemias/*373*, brain tumors/*245*, other tumors/*354*<br>Secondary outcomes: Subgroups of the "other tumors"-category; kidney tumors/*96*, eye tumors/*37*, bone tumors/*56* (only reported for most commonly used medications)	Case-control/*1944*<br>*Finland, ages 0-14 years*<br>MacNemar's test, descriptive for rarely used medications	Matching on date of birth
Schüz, 2007 (21)	Analgesics/*142*<br>*Maternal retrospective report from questionnaire data validated through phone interview*	Classification system not stated<br>ALL/*650*, AML/*105*, NHL/*172*, CNS tumors/*399*, neuroblastoma/*157*, nephroblastoma/*147*, bone tumor/*97*, soft tissue sarcoma/*137*	Case-control/*2652*<br>*Germany, ages 0-14 years*<br>Frequency matched conditional logistic regression	Matching on sex, date of birth, and district. Adjustment for age, sex, year of birth, degree of urbanization, and socioeconomic position
Sharpe & Franco, 1996 (22)	Dipyrone/*70*, Analgesics (other than NSAIDs)/*145*, NSAIDs/*24*<br>*Maternal retrospective report*	Classification system not stated<br>Nephroblastoma/*109*	Case-control/*327*<br>*Brazil, age range not reported, mean age 3 years and 5 months*<br>Logistic regression	Matching on age, sex, and interviewer, stratified on socioeconomic position
Shaw, 2004 (23)	Anti-inflammatories/*4*, Analgesics/*29*<br>*Maternal retrospective report*	Classification: ICD<br>ALL/*789*	Case-control/*1578*<br>*Canada, age 0-14 years*<br>Conditional logistic regression	Matching on sex and age at diagnosis. Adjustment for maternal age and education
Shu, 1995 (24)	Pain relievers/*209*<br>*Maternal retrospective report*	Classification system not stated<br>Malignant germ-cell tumors/*105*	Case-control/*744*<br>*US and Canada, CCG (Children's Cancer Group), ages 0-15 years*	Matching on telephone area code. Adjustment for child age, sex, gestational age, number of siblings, and

Reference<br>First author, year	Exposure<br>Medication/*exposed*<br>*Ascertainment*<br>Exposure window: during pregnancy, unless otherwise stated	Outcome<br>Classification system, type of cancer/*cases*	Materials and methods<br>Design/*sample size*<br>*Study characteristics*<br>Statistical analysis	 Method to account for confounding in the main analysis
			Unconditional logistic regression	maternal education and smoking
Stålberg, 2010 (25)	Any analgesics/*71*, Acetyl salicylic acid or NSAID/*39*, Opioids/*25*, Acetaminophen/*13*<br>*Routinely collected health data*	Classification: ICD<br>Brain tumor (ICD-7 code 193)/*512*	Case-control/*1037*<br>*Sweden, ages 0-15 years*<br>Logistic regression	Matching on sex and birth year. Adjustment for maternal age, parity, country of birth, and level of hospital (primary, secondary, or tertiary)
Wang, 2019 (26)	Antipyretic analgesia intake 6 months before or during pregnancy/*99*<br>*Maternal retrospective report*	Classification system not stated<br>ALL/*345*	Case-control/*690*<br>*China, ages 0-15 years*<br>Unconditional logistic regression	Matching on age, sex, and residence region. Adjustment for age, sex, residence region, birth weight, delivery mode, family history of cancer, income, exposure to pesticides (unclear whether this refers to mother or child), parental age, education, paternal exposure to pesticides, paternal cigarette smoking, and alcohol use
Wen, 2002 (27)	Anti-inflammatory drugs/*18*<br>Pain relievers/*61*<br>Each of the above in the year before pregnancy, through pregnancy and/or during breastfeeding<br>*Maternal retrospective report*	Classification system not stated<br>ALL/*1842*	Case-control/*3828*<br>*US, ages 0-14 years*<br>Conditional logistic regression	Matching on age, ethnicity, and telephone area. Adjustment for parental income, education, ethnicity, smoking and alcohol use
Zanrosso, 2010 (28)	Dipyrone/*102*<br>*Maternal retrospective report*	Classification system not stated<br>IL/*132*	Case-control/*263*<br>*Brazil, ages 0-21 years*<br>Unconditional logistic regression	Matching on age, no adjustment for the association between medication use and IL
ANTACIDS				
Bonaventure, 2015 (1)	Alimentary tract and metabolism (ATC group A)/*789*	Classification: ICD	Case-control/*4122*	Matching on sex, month and year of birth, and region of

Reference	Exposure	Outcome	Materials and methods	
First author, year	Medication/*exposed*<br>*Ascertainment*<br>Exposure window: during pregnancy, unless otherwise stated	Classification system, type of cancer/*cases*	Design/*sample size*<br>*Study characteristics*<br>Statistical analysis	Method to account for confounding in the main analysis
	Routinely collected health data	ALL/*725*, AML/*128*, NHL/*83*, HL/*31*, astrocytoma/*100*, medulloblastoma/*48*, rhabdomyosarcoma/*54,* Ewings sarcoma/*20*, neuroblastoma/*78*, nephroblasoma/*60*	*UK, UKCCS, mean age 5.8 (max age 15 years)*<br>Unconditional logistic regression	residence. Adjustment for year of birth
Gilman, 1989 (6)	Antacids/*150*<br>*Maternal retrospective report and medical records. Children are considered exposed if medication use is present in either source*	Classification system not stated<br>Any cancer/*8059*	Case-control/*16,118*<br>*UK, OSCC, age not specified, only that cases are childhood deaths from cancer*<br>Conditional logistic regression	Matching on date of birth, sex and district. Adjustment for abdominal x-rays in pregnancy, birth order among live born siblings, maternal age, and socioeconomic position
Hartley, 1988 (8)	Antacids/*not reported*<br>*Maternal retrospective report, validated in prescription data*	Classification system not stated<br>Bone or soft tissue sarcomas/*73*	Case-control/*219*<br>*UK, IRESCC, ages 0-15 years*<br>Mantel-Haenszel, 95% CI by Cornfield's formula	Matching. Matching factors not reported, but they refer to a paper where methods are described (age and sex)
McKinney, 1999 (15)	Antacids/*28*<br>*Routinely collected health data*	Classification: ICCC<br>Main outcome: Leukemia/*144*<br>Secondary outcome: ALL/*124*, lymphomas/*45*, CNS tumors/*75*, other solid tumors/*126*	Case-control/*415*<br>*Scotland, ages 0-14 years*<br>Conditional logistic regression	Matching on age, area of residence, and sex
Michalek, 1996 (29)	Antacids/*116*<br>*Maternal retrospective report*	Classification system not stated<br>Neuroblastoma/*183*	Case-control/*555*<br>*US, ages 0-14 years*<br>Unconditional logistic regression	Matching on year of birth
Salonen, 1976 (20)	Antacids/*27*<br>*Routinely collected health data*	Classification: ICD<br>Main outcomes: Leukemias/*373*, brain tumors/*245*, other tumors/*354*<br>Secondary outcomes: Subgroups of the "other tumors"-category; kidney tumors/*96*, eye tumors/*37*,	Case-control/*1944*<br>*Finland, ages 0-14 years*<br>MacNemar's test, descriptive for rarely used medications	Matching on date of birth

Reference	Exposure	Outcome	Materials and methods	
First author, year	Medication/*exposed*<br>*Ascertainment*<br>Exposure window: during pregnancy, unless otherwise stated	Classification system, type of cancer/*cases*	Design/*sample size*<br>*Study characteristics*<br>Statistical analysis	Method to account for confounding in the main analysis
		bone tumors/*56* (only reported for most commonly used medications)		
Shaw, 2004 (23)	Antacids/*61*<br>*Maternal retrospective report*	Classification: ICD<br>ALL/*789*	Case-control/*1578*<br>*Canada, age 0-14 years*<br>Conditional logistic regression	Matching on sex and age at diagnosis. Adjustment for maternal age and education
Stålberg, 2010 (25)	Alimentary tract (ATC group A), including mainly antacid and laxatives/*102*<br>*Routinely collected health data*	Classification: ICD<br>Brain tumor (ICD-7 code 193)/*512*	Case-control/*1037*<br>*Sweden, ages 0-15 years*<br>Logistic regression	Matching on sex and birth year. Adjustment for maternal age, parity, country of birth, and level of hospital (primary, secondary, or tertiary)
ANTIDIABETICS				
Gilman, 1989 (6)	Insulin/*13*<br>*Maternal retrospective report and medical records. Children are considered exposed if medication use is present in either source*	Classification system not stated<br>Any cancer/*8059*	Case-control/*16,118*<br>*UK, OSCC, age not specified, only that cases are childhood deaths from cancer*<br>Conditional logistic regression	Matching on date of birth, sex and district. Adjustment for abdominal x-rays in pregnancy, birth order among live born siblings, maternal age, and socioeconomic position
Ognjanovic, 2009 (30)	Insulin/*8*<br>*Maternal retrospective report*	Classification system not stated<br>Main outcome: Leukemias/*158*<br>Secondary outcomes: ALL/*97*, AML/*61*	Case-control/*331*<br>*US, Children's Oncology Group (US), ages 0-19 years, all cases and controls have Down syndrome*<br>Descriptive statistics	Matching on age and sex
Seppälä, 2020 (31)	Insulin only/*127*<br>Metformin only/*48*<br>Insulin and metformin/*12*<br>*Routinely collected health data*	Classification: ICCC<br>Main outcome: Any cancer/*2029*<br>Secondary outcomes: Leukemias/*649*, lymphomas/*149*, CNS tumors/*484*, other cancers/*743* (not reported for medication exposure)	Case-cohort/*12,132*<br>*Finland, ages 0-19 years*<br>Conditional logistic regression	Adjustment for maternal age, parity, and smoking

Reference	Exposure	Outcome	Materials and methods	
First author, year	Medication/*exposed*<br>*Ascertainment*<br>Exposure window: during pregnancy, unless otherwise stated	Classification system, type of cancer/*cases*	Design/*sample size*<br>*Study characteristics*<br>Statistical analysis	Method to account for confounding in the main analysis
Shaw, 2004 (23)	Diabetes medications/*9*<br>*Maternal retrospective report*	Classification: ICD<br>ALL/*789*	Case-control/*1578*<br>*Canada, age 0-14 years*<br>Conditional logistic regression	Matching on sex and age at diagnosis. Adjustment for maternal age and education
Søegaard, 2018 (32)	Insulin/*3857*<br>*Routinely collected health data*	Classification: ICD<br>ALL/*492*	Cohort study/*1,187,482*<br>*Denmark, age 0-15 years*<br>Cox proportional hazards models	No adjustment for the analysis on medication exposure
ANTIHISTAMINES				
Buckley, 1989 (33)	Antihistamines in the year before pregnancy or during pregnancy/*9*<br>*Maternal retrospective report*	Classification system not stated<br>Hepatoblastoma/*75*	Case-control/*150*<br>*US and Canada, CCSG, upper age limit not stated, but the majority of children were under 2 years of age*<br>Conditional logistic regression	Matching on age
Bunin, 1989 (34)	Antihistamines/*30*<br>*Maternal retrospective report*	Classification system not stated<br>Nonheritable retinoblastoma/*115*<br>Sporadic heritable retinoblastoma (bilateral cases or unilateral cases with 13q chromosomal deletion)/*67*<br>Cases with family history were excluded	Case-control/*383*<br>*US and Canada, CCSG, upper age limit not stated*<br>Conditional logistic regression	Matching on telephone area code, year of birth and ethnicity. Adjustment for paternal education
Bunin, 1994 (2)	Antihistamines/*41*<br>*Maternal retrospective report*	Classification system not stated<br>Astrocytoma/*155*<br>PNET/*166*	Case-control/*332*<br>*US and Canada, Children's Cancer Group, ages 0-5 years*<br>Conditional logistic regression	Matching on telephone area code, year of birth and ethnicity. Adjustment for income (only for astrocytoma)
Carozza, 1995 (3)	Chlorpheniramine/*2*, Chlorpheniramine maleate/*17*, Cimetidine/*3*, Promethazine HCL/*1*<br>*Maternal retrospective report*	Classification: Histological Typing of Tumors of the Central Nervous System. International Histological Classification of Tumors	Case-control/*1444*<br>*US, SEER, upper age limit 18 years*<br>Conditional logistic regression, for main exposure (any n-	Matching on age, sex and maternal ethnicity

Reference	Exposure	Outcome	Materials and methods	
First author, year	Medication/*exposed*<br>*Ascertainment*<br>Exposure window: during pregnancy, unless otherwise stated	Classification system, type of cancer/*cases*	Design/*sample size*<br>*Study characteristics*<br>Statistical analysis	Method to account for confounding in the main analysis
		Any brain tumors/*361*	nitrosatable drugs), descriptive statistics for medications stated here	
Cardy, 2006 (35)	Antihistamines/*204*<br>*Maternal retrospective report*	Classification: ICD-O<br>Main outcome:<br>Any brain tumor/*1218*<br>Secondary outcomes:<br>Astroglial/*623*, PNET/*259*, other glial/*327* by morphological codes	Case-control/*3441*<br>*Europe, North America, Australia, SEARCH, upper age limit 19 years*<br>Unconditional logistic regression	Matching, not further specified. Adjustment for center, age, sex, year of birth, and parental education. Vitamin supplementation (unclear whether in mothers or children) was considered as both a confounder and effect modifier
Cook, 2004 (4)	Chlorpheniramine/*44*, Diphenhydramine/*18*, Doxylamine/*11*,<br>Brompheniramine/*12*, Promethazine/*11*<br>Each during pregnancy or breastfeeding<br>*Maternal retrospective report*	Classification system not stated<br>Neuroblastoma/*504*	Case-control/*1008*<br>*US and Canada, Children's Cancer Group, upper age limit 18 years*<br>Conditional logistic regression	Matching on age. Adjustment for maternal age, education, and ethnicity
Cordier, 1994 (36)	Antihistamines/*7*<br>*Maternal retrospective report*	Classification system not stated<br>Brain tumors/*75*	Case-control/*188*<br>*France, ages 0-15 years*<br>Conditional logistic regression	Matching on birth year. Adjustment for age, sex and maternal age
Kuijten, 1990 (12)	Antihistamines/*not reported*<br>*Maternal retrospective report*	Classification system not stated<br>Astrocytoma/*163*	Case-control/*326*<br>*US, ages 0-14 years*<br>Conditional logistic regression	Matching on telephone area code, ethnicity, and age
McCredie, 1994 (13)	Oral antihistamines in the month before or during pregnancy/*7*<br>*Maternal retrospective report*	Classification: ICD<br>Brain or cranial nerves tumors/*82*	Case-control/*246*<br>*Australia, ages 0-14 years*<br>Conditional logistic regression	Matching on sex and age. Adjustment for parental education
McKinney, 1987 (14)	Antihistamines/*not reported*<br>*Maternal retrospective report and medical records, findings from the two data sources are reported separately*	Classification system not stated<br>Main outcome:<br>Leukemias/lymphomas/*234*<br>Secondary outcomes:<br>Leukemias/*171*<br>Only reported separately for significant associations	Case-control/*702*<br>*UK, IRESCC, ages 0-15 years*<br>Mantel-Haenszel	Matching on age and sex

Reference	Exposure	Outcome	Materials and methods	
First author, year	Medication/*exposed*<br>*Ascertainment*<br>Exposure window: during pregnancy, unless otherwise stated	Classification system, type of cancer/*cases*	Design/*sample size*<br>*Study characteristics*<br>Statistical analysis	Method to account for confounding in the main analysis
Preston-Martin, 1982 (18)	Antihistamines/*31 discordant pairs*<br>*Maternal retrospective report*	Classification system not stated<br>Tumor of the brain or cranial meninges, excluding tumors in infants/*209*	Case-control/*418*<br>*US, ages 0-25 years*<br>Conditional logistic regression	Matching on sex, ethnicity, birth year, and socioeconomic status
Ross, 2003 (19)	Diphenhydramine/*11* , Promethazine/*23*<br>*Routinely collected health data*	Classification system not stated<br>IL/*243*, ALL/*157*, AML/*77*	Case-control/*636*<br>*US, ages 0-18 months*<br>Conditional logistic regression	Matching on birth year and telephone area. Adjustment for maternal age, education and income
Robison, 1989 (37)	Antihistamine in the year before pregnancy, through pregnancy and/or during breastfeeding/*38*<br>Also analyses by duration and frequency<br>*Maternal retrospective report, validated by comparisons to associations in four other independent case-control studies where cases of different cancers did not report more medication use than controls*	Classification system not stated<br>ANLL/*204*	Case-control/*408*<br>*US, ages 0-18 years*<br>Conditional logistic regression	Matching on date of birth, ethnicity, and telephone area code, stated that they adjust for confounders, but these are not specified
Sharpe & Franco, 1996 (22)	Antiallergics/*3*<br>*Maternal retrospective report*	Classification system not stated<br>Nephroblastoma/*109*	Case-control/*327*<br>*Brazil, age range not reported, mean age 3 years and 5 months*<br>Logistic regression	Matching on age, sex, and interviewer, stratified on socioeconomic position
Shaw, 2004 (23)	Antihistamine/*11*<br>*Maternal retrospective report*	Classification: ICD<br>ALL/*789*	Case-control/*1578*<br>*Canada, age 0-14 years*<br>Conditional logistic regression	Matching on sex and age at diagnosis. Adjustment for maternal age and education
Stålberg, 2010 (25)	Antihistamines/*100*<br>*Routinely collected health data*	Classification: ICD<br>Brain tumor (ICD-7 code 193)/*512*	Case-control/*1037*<br>*Sweden, ages 0-15 years*<br>Logistic regression	Matching on sex and birth year. Adjustment for maternal age, parity, country of birth, and level of hospital (primary, secondary, or tertiary)
Wen, 2002 (27)	Antihistamines or allergy remedies in the year before pregnancy, through pregnancy and/or during breastfeeding/*125*<br>*Maternal retrospective report*	Classification system not stated<br>ALL/*1842*	Case-control/*3828*<br>*US, ages 0-14 years*<br>Conditional logistic regression	Matching on age, ethnicity, and telephone area. Adjustment for parental

Reference	Exposure	Outcome	Materials and methods	
First author, year	Medication/*exposed*<br>*Ascertainment*<br>Exposure window: during pregnancy, unless otherwise stated	Classification system, type of cancer/*cases*	Design/*sample size*<br>*Study characteristics*<br>Statistical analysis	Method to account for confounding in the main analysis
				income, education, ethnicity, smoking and alcohol use
ANTIHYPERTENSIVES				
Buckley, 1989 (33)	Antihypertensives/*1*, Diuretics/*10*<br>Each in the year before pregnancy or during pregnancy<br>*Maternal retrospective report*	Classification system not stated<br>Hepatoblastoma/*75*	Case-control/*150*<br>*US and Canada, CCSG, upper age limit not stated, but the majority of children were under 2 years of age*<br>Conditional logistic regression	Matching on age
Bunin, 1987 (38)	Diuretics in the year prior to birth/*10*<br>*Maternal retrospective report*	Classification system not stated<br>Nephroblastoma/*88*	Case-control/*176*<br>*US, ages 0-15 years*<br>Conditional logistic regression	Matching on telephone area code, year of birth and ethnicity. Adjustment for demographic characteristics (not specified further)
Cardy, 2006 (35)	Diuretics/*89*<br>*Maternal retrospective report*	Classification: ICD-O<br>Main outcome:<br>Any brain tumor/*1218*<br>Secondary outcomes:<br>Astroglial/*623*, PNET/*259*, other glial/*327* by morphological codes	Case-control/*3441*<br>*Europe, North America, Australia, SEARCH, upper age limit 19 years*<br>Unconditional logistic regression	Matching, not further specified. Adjustment for center, age, sex, year of birth, and parental education. Vitamin supplementation (unclear whether in mothers or children) was considered as both a confounder and effect modifier
Carozza, 1995 (3)	Furosemide/*1*, Hydrochlorothiazide/*2*<br>*Maternal retrospective report*	Classification: Histological Typing of Tumors of the Central Nervous System. International Histological Classification of Tumors<br>Any brain tumors/*361*	Case-control/*1444*<br>*US, SEER, upper age limit 18 years*<br>Conditional logistic regression, for main exposure (any n-nitrosatable drugs), descriptive statistics for medications stated here	Matching on age, sex and maternal ethnicity

Reference	Exposure	Outcome	Materials and methods	
First author, year	Medication/*exposed*<br>*Ascertainment*<br>Exposure window: during pregnancy, unless otherwise stated	Classification system, type of cancer/*cases*	Design/*sample size*<br>*Study characteristics*<br>Statistical analysis	Method to account for confounding in the main analysis
Gilman, 1989 (6)	Antihypertensives/*88*, Diuretics/*81*<br>*Maternal retrospective report and medical record. Children are considered exposed if medication use is present in either source*	Classification system not stated<br>Any cancer/*8059*	Case-control/*16,118*<br>*UK, OSCC, age not specified, only that cases are childhood deaths from cancer*<br>Conditional logistic regression	Matching on date of birth, sex and district. Adjustment for abdominal x-rays in pregnancy, birth order among live born siblings, maternal age, and socioeconomic position
Hartley, 1988 (8)	Diuretics/*not reported*<br>*Maternal retrospective report, validated in prescription data*	Classification system not stated<br>Bone or soft tissue sarcomas/*73*	Case-control/*219*<br>*UK, IRESCC, ages 0-15 years*<br>Mantel-Haenszel, 95% CI by Cornfield's formula	Matching. Matching factors not reported, but they refer to a paper where methods are described (age and sex)
Kramer, 1987 (11)	Diuretics/*not reported*<br>*Maternal retrospective report, validated in medical records for a subsample. Validity compared between cases and controls*	Classification system not stated<br>Neuroblastoma/*104*	Case-control/*205*<br>*US, median age 1 year*<br>90% CI calculated by Miettinen's technique	Matching on telephone area code, ethnicity, and year of birth
Kuijten, 1990 (12)	Diuretics/*not reported*<br>*Maternal retrospective report*	Classification system not stated<br>Astrocytoma/*163*	Case-control/*326*<br>*US, ages 0-14 years*<br>Conditional logistic regression	Matching on telephone area code, ethnicity, and age
McCredie, 1994 (13)	Diuretics in the month before or during pregnancy/*5*<br>*Maternal retrospective report*	Classification: ICD<br>Brain or cranial nerves tumors/*82*	Case-control/*246*<br>*Australia, ages 0-14 years*<br>Conditional logistic regression	Matching on sex and age. Adjustment for parental education
McKinney, 1987 (14)	Diuretics/*not reported*<br>*Maternal retrospective report and medical records, findings from the two data sources are reported separately*	Classification system not stated<br>Main outcome: Leukemias/lymphomas/*234*<br>Secondary outcomes: Leukemias/*171*<br>Only reported separately for significant associations	Case-control/*702*<br>*UK, IRESCC, ages 0-15 years*<br>Mantel-Haenszel	Matching on age and sex
Michalek, 1996 (29)	Diuretics/*11*<br>*Maternal retrospective report*	Classification system not stated<br>Neuroblastoma/*183*	Case-control/*555*<br>*US, ages 0-14 years*<br>Unconditional logistic regression	Matching on year of birth

Reference	Exposure	Outcome	Materials and methods	
First author, year	Medication/*exposed*<br>*Ascertainment*<br>Exposure window: during pregnancy, unless otherwise stated	Classification system, type of cancer/*cases*	Design/*sample size*<br>*Study characteristics*<br>Statistical analysis	Method to account for confounding in the main analysis
Olshan, 1993 (39)	Diuretics/*13*<br>Blood pressure medication/*4*<br>*Maternal retrospective report*	Classification system not stated<br>Nephroblastoma/*200*	Case-control/*433*<br>*US, ages 0-15 years*<br>Conditional logistic regression	Matching on age and geographic area. Adjustment for income, and paternal education
Preston-Martin, 1982 (18)	Diuretics/*39 discordant pairs*<br>*Maternal retrospective report*	Classification system not stated<br>Tumor of the brain or cranial meninges, excluding tumors in infants/*209*	Case-control/*418*<br>*US, ages 0-25 years*<br>Conditional logistic regression	Matching on sex, ethnicity, birth year, and socioeconomic status
Robison, 1989 (37)	Diuretics/*39*, Blood pressure/*4*<br>Each of the above in the year before pregnancy, through pregnancy and/or during breastfeeding. Also analyses by duration and frequency<br>*Maternal retrospective report, validated by comparisons to associations in four other independent case-control studies where cases of different cancers did not report more medication use than controls*	Classification system not stated<br>ANLL/*204*	Case-control/*408*<br>*US, ages 0-18 years*<br>Conditional logistic regression	Matching on date of birth, ethnicity, and telephone area code, stated that they adjust for confounders, but these are not specified
Salonen, 1976 (20)	Diuretics/*122*, Antihypertensives/*25*<br>*Routinely collected health data*	Classification: ICD<br>Main outcomes: Leukemias/*373*, brain tumors/*245*, other tumors/*354*<br>Secondary outcomes: Subgroups of the "other tumors"-category; kidney tumors/*96*, eye tumors/*37*, bone tumors/*56* (only reported for most commonly used medications)	Case-control/*1944*<br>*Finland, ages 0-14 years*<br>MacNemar's test, descriptive for rarely used medications	Matching on date of birth
Schüz, 2007 (21)	Antihypertensives/diuretics/*40*<br>*Maternal retrospective report from questionnaire data validated through phone interview*	Classification system not stated<br>ALL/*650*, AML/*105*, NHL/*172*, CNS tumors/*399*, neuroblastoma/*157*, nephroblastoma/*147*, bone tumor/*97*, soft tissue sarcoma/*137*	Case-control/*2652*<br>*Germany, ages 0-14 years*<br>Frequency matched conditional logistic regression	Matching on sex, date of birth, and district. Adjustment for age, sex, year of birth, degree of urbanization, and socioeconomic position

Reference	Exposure	Outcome	Materials and methods	
First author, year	Medication/*exposed*<br>*Ascertainment*<br>Exposure window: during pregnancy, unless otherwise stated	Classification system, type of cancer/*cases*	Design/*sample size*<br>*Study characteristics*<br>Statistical analysis	Method to account for confounding in the main analysis
Sharpe & Franco, 1996 (22)	Antihypertensives/*19*<br>*Maternal retrospective report*	Classification system not stated<br>Nephroblastoma/*109*	Case-control/*327*<br>*Brazil, age range not reported, mean age 3 years and 5 months*<br>Logistic regression	Matching on age, sex, and interviewer, stratified on socioeconomic position
Shu, 1995 (24)	Blood pressure/*6*<br>*Maternal retrospective report*	Classification system not stated<br>Malignant germ-cell tumors/*105*	Case-control/*744*<br>*US and Canada, CCG (Children's Cancer Group), ages 0-15 years*<br>Unconditional logistic regression	Matching on telephone area code. Adjustment for child age, sex, gestational age, number of siblings, maternal education, and smoking
Stålberg, 2010 (25)	Antihypertensive/*25*, α-blockers/*16*, β-blockers/*12*, Diuretics/*85*<br>*Routinely collected health data*	Classification: ICD<br>Brain tumor (ICD-7 code 193)/*512*	Case-control/*1037*<br>*Sweden, ages 0-15 years*<br>Logistic regression	Matching on sex and birth year. Adjustment for maternal age, parity, country of birth, and level of hospital (primary, secondary, or tertiary)
Wen, 2002 (27)	Diuretics or Lasix/*27*, Blood pressure tablets/*26*<br>Each of the above in the year before pregnancy, through pregnancy and/or during breastfeeding<br>*Maternal retrospective report*	Classification system not stated<br>ALL/*1842*	Case-control/*3828*<br>*US, ages 0-14 years*<br>Conditional logistic regression	Matching on age, ethnicity, and telephone area. Adjustment for parental income, education, ethnicity, smoking and alcohol use
ANTI-INFECTIVES				
Bonaventure, 2015 (1)	Systemic antibacterials (ATC group J01)/*942*, Penicillins (ATC group J01C)/*846*<br>*Routinely collected health data*	Classification: ICD<br>ALL/*725*, AML/*128*, NHL/*83*, HL/*31*, astrocytoma/*100*, medulloblastoma/*48*, rhabdomyosarcoma/*54*, Ewings sarcoma/*20*, neuroblastoma/*78*, nephroblasoma/*60*	Case-control/*4122*<br>*UK, UKCCS, mean age 5.8 (max age 15 years)*<br>Unconditional logistic regression	Matching on sex, month and year of birth, and region of residence. Adjustment for year of birth
Buckley, 1989 (33)	Antibiotics in the year before pregnancy or during pregnancy/*31*<br>*Maternal retrospective report*	Classification system not stated<br>Hepatoblastoma/*75*	Case-control/*150*<br>*US and Canada, CCSG, upper age limit not stated, but the*	Matching on age

Reference<br>First author, year	Exposure<br>Medication/*exposed*<br>*Ascertainment*<br>Exposure window: during pregnancy, unless otherwise stated	Outcome<br>Classification system, type of cancer/*cases*	Materials and methods<br>Design/*sample size*<br>*Study characteristics*<br>Statistical analysis	 Method to account for confounding in the main analysis
			majority of children were under 2 years of age<br>Conditional logistic regression	
Bunin, 1994 (2)	Medication against vaginal infection/*59*<br>*Maternal retrospective report*	Classification system not stated<br>Astrocytoma/*155*<br>PNET/*166*	Case-control/*332*<br>*US and Canada, Children's Cancer Group, ages 0-5 years*<br>Conditional logistic regression	Matching on telephone area code, year of birth and ethnicity. Adjustment for income (only for astrocytoma)
Carozza, 1995 (3)	Ampicillin/*13*, Ampicillin trihydrate/*1*, Doxycline/*2*, Erythromycin/*4*, Oxytetracycline/*2*, Penicillin V potassium/*7*, Tetracycline HCL/*1*<br>*Maternal retrospective report*	Classification: Histological Typing of Tumors of the Central Nervous System. International Histological Classification of Tumors<br>Any brain tumors/*361*	Case-control/*1444*<br>*US, SEER, upper age limit 18 years*<br>Conditional logistic regression, for main exposure (any n-nitrosatable drugs), descriptive statistics for medications stated here	Matching on age, sex and maternal ethnicity
Cook, 2004 (4)	Amoxicillin/*102*, Ampicillin/*21*, Cephalexin/*17*, Erythromycin/*27*, Nitrofurantoin/*5*, Penicillin/*23*, Sulfamethoxazole/*14*, Trimethoprim/*15*<br>Each during pregnancy or breastfeeding<br>*Maternal retrospective report*	Classification system not stated<br>Neuroblastoma/*504*	Case-control/*1008*<br>*US and Canada, Children's Cancer Group, upper age limit 18 years*<br>Conditional logistic regression	Matching on age. Adjustment for maternal age, education, and ethnicity
Gilman, 1989 (6)	Antibiotics/*631*, Antimalarials/*16*<br>Antituberculosis drugs/*13*, Sulphonamides/*116*, Vaccines/*235*<br>*Maternal retrospective report and medical records. Children are considered exposed if medication use is present in either source*	Classification system not stated<br>Any cancer/*8059*	Case-control/*16,118*<br>*UK, OSCC, age not specified, only that cases are childhood deaths from cancer*<br>Conditional logistic regression	Matching on date of birth, sex and district. Adjustment for abdominal x-rays in pregnancy, birth order among live born siblings, maternal age, and socioeconomic position
Gradel & Kaerlev, 2015 (40)	Antibiotics/*1201*<br>Secondary exposures:<br>Narrow spectrum β-lactams/*714*,<br>Broad spectrum β-lactams/*715*,	Classification: ICD<br>Main outcome:<br>Any leukemias/*360*<br>Secondary outcome:	Case-control/*3869*<br>*Denmark, ages 0-15 years*<br>Conditional logistic regression	Matching on age, sex, and municipality. Adjustment for birth weight, birth order, maternal age, migration,

Reference	Exposure	Outcome	Materials and methods	
First author, year	Medication/*exposed*<br>*Ascertainment*<br>Exposure window: during pregnancy, unless otherwise stated	Classification system, type of cancer/*cases*	Design/*sample size*<br>*Study characteristics*<br>Statistical analysis	Method to account for confounding in the main analysis
	Sulphonamides or trimethoprim/*317*, Macrolides/*147*<br>Also analysis by number of treatments<br>*Routinely collected health data*	ALL/*284*		education, labor market affiliation, and income
Grufferman, 1982 (7)	Antibiotics 1 year prior to or during pregnancy/*32*<br>*Maternal retrospective report*	Classification system not stated<br>Rhabdomyosarcoma/*33*	Case-control/*132*<br>*US, ages 0-14 years*<br>"Unmatched methods", not specified further	Matching on age, sex, and ethnicity
Hartley, 1988 (8)	Urinary anti-infectives/*not reported*<br>*Maternal retrospective report, validated in prescription data*	Classification system not stated<br>Bone or soft tissue sarcomas/*73*	Case-control/*219*<br>*UK, IRESCC, ages 0-15 years*<br>Mantel-Haenszel, 95% CI by Cornfield's formula	Matching. Matching factors not reported, but they refer to a paper where methods are described (age and sex)
Heck, 2015 (9)	Antibiotics/*53*<br>*Maternal retrospective report*	Classification system not stated<br>Sporadic retinoblastoma, unilateral/*187*, Sporadic retinoblastoma, bilateral/*95*	Case-control/*426*<br>*US/Canada, ages 0-15 years*<br>Unconditional logistic regression	Matching on year of birth. Adjustment for maternal ethnicity, education, income, age, and smoking in pregnancy
Infante-Rivard, 2000 (41)	Antibiotics/*118*<br>*Maternal retrospective report*	Classification: ICD<br>ALL/*491*	Case-control/*982*<br>*Canada, upper age limit 10 years*<br>Conditional logistic regression	Matching on sex, age, and area of residence. Adjustment for maternal age and education
Kaatsch, 2010 (42)	Antibiotics/*224*<br>*Maternal retrospective report*	Classification: ICCC<br>Main outcome: Any cancer/*1867*<br>Secondary outcomes: ALL/*650*, AML/*105*, Burkitt lymphoma/*12*, NHL/*141*, medulloblastoma/*94*, astrocytoma/*92*, ependymoma/*44*, neuroblastoma/*160*, nephroblastoma/*147*, bone tumor/*97*, soft tissue sarcoma/*137*	Case-control/*3924*<br>*Germany, 0-14 years*<br>Conditional logistic regression	Matching on community, sex, and year of birth. Adjustment for sex, age, degree of urbanization, and socioeconomic position
Kumar, 2014 (43)	Chloramphenicol/*not reported*<br>*Maternal retrospective report*	Classification system not stated<br>ALL or AML/*132*	Case-control/*264*<br>*India, ages 0-17 years*<br>z-test for two proportions	Matching on age, sex, and residency

Reference	Exposure	Outcome	Materials and methods	
First author, year	Medication/*exposed*<br>*Ascertainment*<br>Exposure window: during pregnancy, unless otherwise stated	Classification system, type of cancer/*cases*	Design/*sample size*<br>*Study characteristics*<br>Statistical analysis	Method to account for confounding in the main analysis
Kwan, 2007 (44)	Antibiotics 3 months before and/or during pregnancy/*128*<br>*Maternal retrospective report*	Classification: ICD-O<br>Main outcome: Leukemia/*365*<br>Secondary outcome: ALL/*311*	Case-control/*825*<br>*US, NCCLS, ages 0-14 years*<br>Conditional logistic regression	Matching on date of birth, sex, ethnicity (of child and mother), and for part of the recruitment also county of residence at birth. Adjustment for maternal age, income, and education
McKinney, 1987 (14)	Antibiotics, Antifungals/*not reported*<br>*Maternal retrospective report and medical records, findings from the two data sources are reported separately*	Classification system not stated<br>Main outcome: Leukemias or lymphomas/*234*<br>Secondary outcomes: Leukemias/*171*<br>Only reported separately for significant associations	Case-control/*702*<br>*UK, IRESCC, ages 0-15 years*<br>Mantel-Haenszel	Matching on age and sex
McKinney, 1999 (15)	Antibiotics/*60*, Topical antifungals/*29*<br>*Routinely collected health data*	Classification: ICCC<br>Main outcome: Leukemia/*144*<br>Secondary outcome: ALL/*124*, lymphomas/*45*, CNS tumors/*75*, other solid tumors/*126*	Case-control/*415*<br>*Scotland, ages 0-14 years*<br>Conditional logistic regression	Matching on age, area of residence, and sex
Michalek, 1996 (29)	Medications for urinary tract infection/*26*, Medications for bladder infection/*12*<br>*Maternal retrospective report*	Classification system not stated<br>Neuroblastoma/*183*	Case-control/*555*<br>*US, ages 0-14 years*<br>Unconditional logistic regression	Matching on year of birth
Momen, 2015 (45)	Main exposure: Antibiotics/*506,194*<br>Secondary exposures: Antibiotics on 3rd ATC-level, selected antibiotics on 5th ATC-level/*not reported*<br>Also analysis by dose<br>*Routinely collected health data*	Classification: ICD<br>Main outcome: Any cancer/*1479*<br>Secondary outcomes: Leukemias/*591*, ALL/*444*, AML/*74*, HL/*23*, NHL/*20*, CNS or SNS tumors/*249*, renal tumors/*121*, hepatic tumors/*29*, endocrine tumors/*93*, testis cancer/*21*, eye cancers/*77*, bone cancers/*44*	Cohort/*1,442,114*<br>*Denmark and Sweden, ages 0-14 years*<br>Cox regression	Adjustment for maternal age, parity, education, smoking during pregnancy, and child country of birth

Reference	Exposure	Outcome	Materials and methods	
First author, year	Medication/*exposed*<br>*Ascertainment*<br>Exposure window: during pregnancy, unless otherwise stated	Classification system, type of cancer/*cases*	Design/*sample size*<br>*Study characteristics*<br>Statistical analysis	Method to account for confounding in the main analysis
Naumburg, 2002 (46)	Local treatment for vaginal infections/*88*, Oral antibiotics/*76*<br>*Routinely collected health data*	Classification: ICD<br>Main outcome: Leukemia/*652*<br>Secondary outcomes: lymphoid leukemias/*578*, myeloid leukemias/*74* (not reported for medication exposures)	Case-cohort/*1304*<br>*Sweden, age 0-16 years*<br>Conditional logistic regression	Matching on sex, birth year and month. Adjustment for maternal age, parity, mode of delivery, smoking, time from membrane rupture until delivery, gestational age, birth weight, and plurality
Olshan, 1993 (39)	Antibiotics/*58*<br>*Maternal retrospective report*	Classification system not stated<br>Nephroblastoma/*200*	Case-control/*433*<br>*US, ages 0-15 years*<br>Conditional logistic regression	Matching on age and geographic area. Adjustment for income, and paternal education
Pombo de Oliveira, 2006 (17)	Amoxicillin/*87*, Ciprofloxacin/*16*<br>Antifungal (metronidazole)/*16*<br>Each of the above 3 months before pregnancy, during pregnancy, or during breastfeeding<br>*Maternal retrospective report*	Classification system not stated<br>Infant acute leukemias/*202*	Case-control/*642*<br>*Brazil, ages 0-21 months*<br>Unconditional logistic regression	Matching on age and region. Adjustment for region, sex, income, maternal age, and birth weight
Robison, 1989 (37)	Antibiotics in the year before pregnancy, through pregnancy and/or during breastfeeding/*97*<br>Also analyses by duration and frequency<br>*Maternal retrospective report, validated by comparisons to associations in four other independent case-control studies where cases of different cancers did not report more medication use than controls*	Classification system not stated<br>ANLL/*204*	Case-control/*408*<br>*US, ages 0-18 years*<br>Conditional logistic regression	Matching on date of birth, ethnicity, and telephone area code, stated that they adjust for confounders, but these are not specified
Rodvall, 1990 (47)	Drugs for infections (unspecified)/*11*<br>*Routinely collected health data*	Classification system not stated<br>Any cancer/*95*	Case-control/*285*<br>*Sweden, ages 0-16 years*<br>Multiple conditional logistic regression	Matching on sex, birth year and month. Adjustment for maternal age, drug use, obstetric complications, previous miscarriage, socioeconomic position and child gestational age.

Reference	Exposure	Outcome	Materials and methods	
First author, year	Medication/*exposed*<br>*Ascertainment*<br>Exposure window: during pregnancy, unless otherwise stated	Classification system, type of cancer/*cases*	Design/*sample size*<br>*Study characteristics*<br>Statistical analysis	Method to account for confounding in the main analysis
Roman, 1997 (48)	Antibiotics/*27*<br>*Routinely collected health data*	Classification system not stated<br>Main outcomes: Leukemias/*143*, NHL/*34*<br>Secondary outcomes: ALL/*113*, AML/*15*	Case-control/*429*<br>*England, ages 3 months to 29 years*<br>Conditional logistic regression	Matching on hospital catchment area of birth, sex, year and month of birth
Ross, 2003 (19)	Amoxicillin/*34*, Ampicillin/*49*, Cefaclor/*7*, Cephalexin/*13*, Clotrimazole/*24*, Cotrimoxazole/*11*, Erythromycin/*43*, Metronidazole/*8*, Miconazole/*61* Nitrofurantoin/*12*, Nystatin/*11*, Penicillin V potassium/*11*, Triple sulfa/*10*, Rh(O) O immunoglobulin/*57*<br>*Routinely collected health data*	Classification system not stated<br>IL/*243*, ALL/*157*, AML/*77*	Case-control/*636*<br>*US, ages 0-18 months*<br>Conditional logistic regression	Matching on birth year and telephone area. Adjustment for maternal age, education and income
Salonen, 1976 (20)	Vaccination/*64*, Sulphonamides/*24*, Penicillins/*28*, Tetracyclines/*9*<br>*Routinely collected health data*	Classification: ICD<br>Main outcomes: Leukemias/*373*, brain tumors/*245*, other tumors/*354*<br>Secondary outcomes: Subgroups of the “other tumors”-category; kidney tumors/*96*, eye tumors/*37*, bone tumors/*56* (only reported for most commonly used medications)	Case-control/*1944*<br>*Finland, ages 0-14 years*<br>MacNemar’s test, descriptive for rarely used medications	Matching on date of birth
Sanders and Draper, 1979 (49)	Isoniazid/*12*, Streptomycin/*9*<br>*Maternal retrospective report and medical records, findings from the two data sources are reported separately*	Classification system not stated<br>Any cancer/*27*	Case-controls/*40*<br>*UK, Oxford Survey of Childhood Cancers, ages 0-15. All children were born to mothers with pulmonary tuberculosis*<br>Descriptive statistics	It would appear that matching was done, but it is unclear
Shaw, 2004 (23)	Antibiotics/*87*<br>*Maternal retrospective report*	Classification: ICD<br>ALL/*789*	Case-control/*1578*<br>*Canada, age 0-14 years*<br>Conditional logistic regression	Matching on sex and age at diagnosis. Adjustment for maternal age and education

Reference	Exposure	Outcome	Materials and methods	
First author, year	Medication/*exposed*<br>*Ascertainment*<br>Exposure window: during pregnancy, unless otherwise stated	Classification system, type of cancer/*cases*	Design/*sample size*<br>*Study characteristics*<br>Statistical analysis	Method to account for confounding in the main analysis
Shaw, 2006 (50)	Antibiotics/*30*<br>*Maternal retrospective report*	Classification: ICD-O<br>Main outcome:<br>CNS tumor/*272*<br>Secondary outcomes:<br>Ependymoma/*27*, astrocytoma/*120*, PNET/*80*	Case-control/*544*<br>*Canada, ages 0-15 years*<br>Conditional logistic regression	Matching on sex and age at diagnosis. Adjustment for maternal education
Shu, 1995 (24)	Antibiotics/*78*<br>*Maternal retrospective report*	Classification system not stated<br>Malignant germ-cell tumors/*105*	Case-control/*744*<br>*US and Canada, CCG (Children's Cancer Group), ages 0-15 years*<br>Unconditional logistic regression	Matching on telephone area code. Adjustment for child age, sex, gestational age, number of siblings, maternal education, and smoking
Stålberg, 2010 (25)	Anti-infectives/*189*, antifungals/*105*, penicillins/*55*, antibiotics, others/*60*<br>*Routinely collected health data*	Classification: ICD<br>Brain tumor (ICD-7 code 193)/*512*	Case-control/*1037*<br>*Sweden, ages 0-15 years*<br>Logistic regression	Matching on sex and birth year. Adjustment for maternal age, parity, country of birth, and level of hospital (primary, secondary, or tertiary)
Thapa, 1998 (51)	Metronidazole/*79,716 person-years*<br>*Routinely collected health data, validated by using two different definitions of no exposure*	Classification: ICD<br>Main outcome: All cancer/*175*<br>Secondary outcomes: leukemias/*42*, CNS tumors/*30*, neuroblastoma/*28*, other cancers/*75*	Cohort/*1,172,696 person-years*<br>*US, ages 0-5 years*<br>Poisson regression	Adjustment for maternal age, residence area, ethnicity, marital status, education, and parity
Van Duijn, 1994 (52)	Antibiotics in the year before or during pregnancy/*8*<br>*Maternal retrospective report, validated by comparison to associations in another case-control study where cases of a different type of cancer did not report more medication use than controls*	Classification system not stated<br>ANLL/*80*	Case-control/*320*<br>*The Netherlands, ages 0-14 years, study reported results for both population- and cancer controls*<br>Conditional logistic regression	Matching on sex and age. Adjustment for sex, year of birth, socioeconomic position, in pregnancy: alcohol use, smoking, occupational hydrocarbon exposure, other medication use, ultrasound, x-rays, viral infections

Reference	Exposure	Outcome	Materials and methods	
First author, year	Medication/*exposed*<br>*Ascertainment*<br>Exposure window: during pregnancy, unless otherwise stated	Classification system, type of cancer/*cases*	Design/*sample size*<br>*Study characteristics*<br>Statistical analysis	Method to account for confounding in the main analysis
Van Steensel-Moll, 1985 (53)	Antibiotics/*27*, Vaccination/*31*<br>*Maternal retrospective report*	Classification: ICD<br>Leukemias/*519*	Case-control/*1026*<br>*The Netherlands, ages 0-15 years*<br>Logistic regression	Matching on date of birth, sex and municipality. Adjustment for age and sex
Walsh, 2019 (54)	H1N1 influenza vaccination/*31,295*<br>*Routinely collected health data*	Classification: ICD<br>Neoplasm/*145*	Cohort/*104,249*<br>*Canada, ages 0-5 years*<br>Cox regression	Inverse probability of treatment weighting. The variables in the propensity score were maternal age, parity, smoking, season of conception, antenatal care provider, pre-existing comorbidity, obstetric complications, antenatal steroid use, income, residence area, public health unit region and multifetal gestation
Wen, 2002 (27)	Antibiotics in the year before pregnancy, through pregnancy and/or during breastfeeding/*400*<br>*Maternal retrospective report*	Classification system not stated<br>ALL/*1842*	Case-control/*3828*<br>*US, ages 0-14 years*<br>Conditional logistic regression	Matching on age, ethnicity, and telephone area. Adjustment for parental income, education, ethnicity, smoking and alcohol use
Ye, 2019 (55)	Antibiotics (ATC class J01)/*98,997*<br>*Routinely collected health data*	Classification: ICCC<br>Main outcome: Any cancer (excluding nonmelanoma skin cancer)/*361*<br>Secondary outcomes: Leukemias/*141*, ALL/*114*	Cohort/*262,116*<br>*Canada, ages 0-19 years*<br>Cox regression	Adjustment for maternal age, income, prenatal use of tobacco, alcohol or illicit drugs
ANTINAUSEANTS				
Buckley, 1989 (33)	Antinauseants in the year before pregnancy or during pregnancy/*19*<br>*Maternal retrospective report*	Classification system not stated<br>Hepatoblastoma/*75*	Case-control/*150*<br>*US and Canada, CCSG, upper age limit not stated, but the*	Matching on age

Reference<br>First author, year	Exposure<br>Medication/*exposed*<br>*Ascertainment*<br>Exposure window: during pregnancy, unless otherwise stated	Outcome<br>Classification system, type of cancer/*cases*	Materials and methods<br>Design/*sample size*<br>*Study characteristics*<br>Statistical analysis	 Method to account for confounding in the main analysis
			majority of children were under 2 years of age<br>Conditional logistic regression	
Bunin, 1989 (34)	Antinauseants/*45*<br>*Maternal retrospective report*	Classification system not stated<br>Nonheritable retinoblastoma/*115*<br>Sporadic heritable retinoblastoma (bilateral cases or unilateral cases with 13q chromosomal deletion)/*67*<br>Cases with family history were excluded	Case-control/*383*<br>*US and Canada, CCSG, upper age limit not stated*<br>Conditional logistic regression	Matching on telephone area code, year of birth and ethnicity. Adjustment for paternal education
Bunin, 1994 (2)	Antinauseants (specific to morning sickness)/*37*, Antinauseants (other)/*11*<br>*Maternal retrospective report*	Classification system not stated<br>Astrocytoma/*155*<br>PNET/*166*	Case-control/*332*<br>*US and Canada, Children's Cancer Group, ages 0-5 years*<br>Conditional logistic regression	Matching on telephone area code, year of birth and ethnicity. Adjustment for income (only for astrocytoma)
Cardy, 2006 (35)	Antiemetics/*33*<br>*Maternal retrospective report*	Classification: ICD-O<br>Main outcome:<br>Any brain tumor/*1218*<br>Secondary outcomes:<br>Astroglial/*623*, PNET/*259*, other glial/*327* by morphological codes	Case-control/*3441*<br>*Europe, North America, Australia, SEARCH, upper age limit 19 years*<br>Unconditional logistic regression	Matching, not further specified. Adjustment for center, age, sex, year of birth, and parental education. Vitamin supplementation (unclear whether in mothers or children) was considered as both a confounder and effect modifier
Gilman, 1989 (6)	Antinauseants/*996*<br>*Maternal retrospective report and medical records. Children are considered exposed if medication use is present in either source*	Classification system not stated<br>Any cancer/*8059*	Case-control/*16,118*<br>*UK, OSCC, age not specified, only that cases are childhood deaths from cancer*<br>Conditional logistic regression	Matching on date of birth, sex and district. Adjustment for abdominal x-rays in pregnancy, birth order among live born siblings, maternal age, and socioeconomic position

Reference	Exposure	Outcome	Materials and methods	
First author, year	Medication/*exposed*<br>*Ascertainment*<br>Exposure window: during pregnancy, unless otherwise stated	Classification system, type of cancer/*cases*	Design/*sample size*<br>*Study characteristics*<br>Statistical analysis	Method to account for confounding in the main analysis
Hartley, 1988 (8)	Antiemetics/*not reported*<br>*Maternal retrospective report, validated in prescription data*	Classification system not stated<br>Bone or soft tissue sarcomas/*73*	Case-control/*219*<br>*UK, IRESCC, ages 0-15 years*<br>Mantel-Haenszel, 95% CI by Cornfield's formula	Matching. Matching factors not reported, but they refer to a paper where methods are described (age and sex)
Kramer, 1987 (11)	Antinauseants/*not reported*<br>*Maternal retrospective report, validated in medical records for a subsample. Validity compared between cases and controls*	Classification system not stated<br>Neuroblastoma/*104*	Case-control/*205*<br>*US, median age 1 year*<br>90% CI calculated by Miettinen's technique	Matching on telephone area code, ethnicity, and year of birth
Kuijten, 1990 (12)	Antinauseants/*54*<br>*Maternal retrospective report*	Classification system not stated<br>Astrocytoma/*163*	Case-control/*326*<br>*US, ages 0-14 years*<br>Conditional logistic regression	Matching on telephone area code, ethnicity, and age
Kwan, 2007 (44)	Antinauseants 3 months before and/or during pregnancy/*44*<br>*Maternal retrospective report*	Classification: ICD-O<br>Main outcome: Leukemia/*365*<br>Secondary outcome: ALL/*311*	Case-control/*825*<br>*US, NCCLS, ages 0-14 years*<br>Conditional logistic regression	Matching on date of birth, sex, ethnicity (of child and mother), and for part of the recruitment also county of residence at birth. Adjustment for maternal age, income, and education
McKinney, 1985 (56)	Debendox/*92*, Other antiemetics/*22*<br>Also analysis by duration<br>*Maternal retrospective report and medical records, findings from the two data sources are reported separately*	Classification system not stated<br>Main outcome: Any cancer/*555*<br>Secondary outcomes: Leukemias or lymphomas/*245*<br>Other cancers/*310*	Case-control/*1665*<br>*UK, IRESCC, ages 0-15 years*<br>Maximum likelihood, not specified further	Matching on age and sex
McKinney, 1999 (15)	Antinauseants/*14*<br>*Routinely collected health data*	Classification: ICCC<br>Main outcome: Leukemia/*144*<br>Secondary outcome: ALL/*124*, lymphomas/*45*, CNS tumors/*75*, other solid tumors/*126*	Case-control/*415*<br>*Scotland, ages 0-14 years*<br>Conditional logistic regression	Matching on age, area of residence, and sex
Michalek, 1996 (29)	Antinauseants/*46*<br>*Maternal retrospective report*	Classification system not stated<br>Neuroblastoma/*183*	Case-control/*555*<br>*US, ages 0-14 years*<br>Unconditional logistic regression	Matching on year of birth

Reference	Exposure	Outcome	Materials and methods	
First author, year	Medication/*exposed*<br>*Ascertainment*<br>Exposure window: during pregnancy, unless otherwise stated	Classification system, type of cancer/*cases*	Design/*sample size*<br>*Study characteristics*<br>Statistical analysis	Method to account for confounding in the main analysis
Pombo de Oliveira, 2006 (17)	Antiemetic 3 months before pregnancy, during pregnancy, or during breastfeeding/*43*<br>*Maternal retrospective report*	Classification system not stated<br>Infant acute leukemias/*202*	Case-control/*642*<br>*Brazil, ages 0-21 months*<br>Unconditional logistic regression	Matching on age and region. Adjustment for region, sex, birth weight, maternal age, and income
Ross, 2003 (19)	Bendectin (Dicyclomine, Doxylamine, Pyridoxine)/*12*<br>Prochlorperazine/*9*<br>Trimethobenzamide HCL/*7*<br>*Routinely collected health data*	Classification system not stated<br>IL/*243*, ALL/*157*, AML/*77*	Case-control/*636*<br>*US, ages 0-18 months*<br>Conditional logistic regression	Matching on birth year and telephone area. Adjustment for maternal age, education and income
Robison, 1989 (37)	Bendectin or other morning sickness tablets in the year before pregnancy, through pregnancy and/or during breastfeeding/*71*<br>Also analyses by duration and frequency<br>*Maternal retrospective report, validated by comparisons to associations in four other independent case-control studies where cases of different cancers did not report more medication use than controls*	Classification system not stated<br>ANLL/*204*	Case-control/*408*<br>*US, ages 0-18 years*<br>Conditional logistic regression	Matching on date of birth, ethnicity, and telephone area code, stated that they adjust for confounders, but these are not specified
Schüz, 2007 (21)	Antinauseants/antiemetics/*113*<br>*Maternal retrospective report from questionnaire data validated through phone interview*	Classification system not stated<br>ALL/*650*, AML/*105*, NHL/*172*, CNS tumors/*399*, neuroblastoma/*157*, nephroblastoma/*147*, bone tumor/*97*, soft tissue sarcoma/*137*	Case-control/*2652*<br>*Germany, ages 0-14 years*<br>Frequency matched conditional logistic regression	Matching on sex, date of birth, and district. Adjustment for age, sex, year of birth, degree of urbanization, and socioeconomic position
Sharpe & Franco, 1996 (22)	Metoclopramide/*50*, Antiemetics/*86*<br>*Maternal retrospective report*	Classification system not stated<br>Nephroblastoma/*109*	Case-control/*327*<br>*Brazil, age range not reported, mean age 3 years and 5 months*<br>Logistic regression	Matching on age, sex, and interviewer, stratified on socioeconomic position
Shaw, 2004 (23)	Anti-nausea medication/*115*<br>*Maternal retrospective report*	Classification: ICD<br>ALL/*789*	Case-control/*1578*<br>*Canada, age 0-14 years*<br>Conditional logistic regression	Matching on sex and age at diagnosis. Adjustment for maternal age and education

Reference	Exposure	Outcome	Materials and methods	
First author, year	Medication/*exposed*<br>*Ascertainment*<br>Exposure window: during pregnancy, unless otherwise stated	Classification system, type of cancer/*cases*	Design/*sample size*<br>*Study characteristics*<br>Statistical analysis	Method to account for confounding in the main analysis
Shu, 1995 (24)	Medicine for nausea or vomiting/*151*<br>*Maternal retrospective report*	Classification system not stated<br>Malignant germ-cell tumors/*105*	Case-control/*744*<br>*US and Canada, CCG (Children's Cancer Group), ages 0-15 years*<br>Unconditional logistic regression	Matching on telephone area code. Adjustment for child age, sex, gestational age, number of siblings, maternal education, and smoking
Stålberg, 2010 (25)	Antiemetics/*106*<br>*Routinely collected health data*	Classification: ICD<br>Brain tumor (ICD-7 code 193)/*512*	Case-control/*1037*<br>*Sweden, ages 0-15 years*<br>Logistic regression	Matching on sex and birth year. Adjustment for maternal age, parity, country of birth, and level of hospital (primary, secondary, or tertiary)
Wen, 2002 (27)	Bendectin or other morning sickness tablets in the year before pregnancy, through pregnancy and/or during breastfeeding/*231*<br>*Maternal retrospective report*	Classification system not stated<br>ALL/*1842*	Case-control/*3828*<br>*US, ages 0-14 years*<br>Conditional logistic regression	Matching on age, ethnicity, and telephone area. Adjustment for parental income, education, ethnicity, smoking and alcohol use
ANTITUSSIVES/ANTI-ASTHMATICS				
Bonaventure, 2015 (1)	Respiratory system (ATC group R)/*409*<br>*Routinely collected health data*	Classification: ICD<br>ALL/*725*, AML/*128*, NHL/*83*, HL/*31*, astrocytoma/*100*, medulloblastoma/*48*, rhabdomyosarcoma/*54*, Ewings sarcoma/*20*, neuroblastoma/*78*, nephroblasoma/*60*	Case-control/*4122*<br>*UK, UKCCS, mean age 5.8 (max age 15 years)*<br>Unconditional logistic regression	Matching on sex, month and year of birth, and region of residence. Adjustment for year of birth
Buckley, 1989 (33)	Cold or cough medicines/*12*<br>*Maternal retrospective report*	Classification system not stated<br>Hepatoblastoma/*75*	Case-control/*150*<br>*US and Canada, CCSG, upper age limit not stated, but the majority of children were under 2 years of age*<br>Conditional logistic regression	Matching on age
Carozza, 1995 (3)	Epinephrine/*1*<br>*Maternal retrospective report*	Classification: Histological Typing of Tumors of the Central	Case-control/*1444*	Matching on age, sex and maternal ethnicity

Reference	Exposure	Outcome	Materials and methods	
First author, year	Medication/*exposed*<br>*Ascertainment*<br>Exposure window: during pregnancy, unless otherwise stated	Classification system, type of cancer/*cases*	Design/*sample size*<br>*Study characteristics*<br>Statistical analysis	Method to account for confounding in the main analysis
		Nervous System. International Histological Classification of Tumors<br>Any brain tumors/*361*	*US, SEER, upper age limit 18 years*<br>Conditional logistic regression, for main exposure (any n-nitrosatable drugs), descriptive statistics for medications stated here	
Cook, 2004 (4)	Albuterol/*8*, Phenylephrine/*14*, Phenylpropanolamine/*25*, Pseudoephedrine/*116*, Terbutaline/*12*, Dextromethorphan/*14*, Guaifenesin/*68*<br>Each of the above during pregnancy or breastfeeding<br>*Maternal retrospective report*	Classification system not stated<br>Neuroblastoma/*504*	Case-control/*1008*<br>*US and Canada, Children's Cancer Group, upper age limit 18 years*<br>Conditional logistic regression	Matching on age. Adjustment for maternal age, education, and ethnicity
Gilman, 1989 (6)	Expectorants/*54*, Bronchospasm relaxants/*78*<br>*Maternal retrospective report and medical records. Children are considered exposed if medication use is present in either source*	Classification system not stated<br>Any cancer/*8059*	Case-control/*16,118*<br>*UK, OSCC, age not specified, only that cases are childhood deaths from cancer*<br>Conditional logistic regression	Matching on date of birth, sex and district. Adjustment for abdominal x-rays in pregnancy, birth order among live born siblings, maternal age, and socioeconomic position
McCredie, 1994 (13)	Cold or cough remedies in the month before or during pregnancy/*7*<br>*Maternal retrospective report*	Classification: ICD<br>Brain or cranial nerves tumors/*82*	Case-control/*246*<br>*Australia, ages 0-14 years*<br>Conditional logistic regression	Matching on sex and age. Adjustment for parental education
Ross, 1996 (57)	Cold medications/*77*<br>*Maternal retrospective report*	Classification system not stated<br>IL/*84*	Case-control/*181*<br>*US, ages 0-1 year*<br>Conditional logistic regression	Matching on year of birth, geography and ethnicity. Adjustment for maternal education
Ross, 2003 (19)	Terbutaline/*15*, Theophylline salts/*6*<br>*Routinely collected health data*	Classification system not stated<br>IL/*243*, ALL/*157*, AML/*77*	Case-control/*636*<br>*US, ages 0-18 months*<br>Conditional logistic regression	Matching on birth year and telephone area. Adjustment for maternal age, education and income

Reference	Exposure	Outcome	Materials and methods	
First author, year	Medication/*exposed*<br>*Ascertainment*<br>Exposure window: during pregnancy, unless otherwise stated	Classification system, type of cancer/*cases*	Design/*sample size*<br>*Study characteristics*<br>Statistical analysis	Method to account for confounding in the main analysis
Robison, 1989 (37)	Cold/cough medication in the year before pregnancy, through pregnancy and/or during breastfeeding/*61*<br>Also analyses by duration and frequency<br>*Maternal retrospective report, validated by comparisons to associations in four other independent case-control studies where cases of different cancers did not report more medication use than controls*	Classification system not stated<br>ANLL/*204*	Case-control/*408*<br>*US, ages 0-18 years*<br>Conditional logistic regression	Matching on date of birth, ethnicity, and telephone area code, stated that they adjust for confounders, but these are not specified
Salonen, 1976 (20)	Parasympatholytics/*20*<br>*Routinely collected health data*	Classification: ICD<br>Main outcomes: Leukemias/*373*, brain tumors/*245*, other tumors/*354*<br>Secondary outcomes: Subgroups of the "other tumors"-category; kidney tumors/*96*, eye tumors/*37*, bone tumors/*56* (only reported for most commonly used medications)	Case-control/*1944*<br>*Finland, ages 0-14 years*<br>MacNemar's test, descriptive for rarely used medications	Matching on date of birth
Schüz, 2007 (21)	Cold medications/*282*<br>*Maternal retrospective report from questionnaire data validated through phone interview*	Classification system not stated<br>ALL/*650*, AML/*105*, NHL/*172*, CNS tumors/*399*, neuroblastoma/*157*, nephroblastoma/*147*, bone tumor/*97*, soft tissue sarcoma/*137*	Case-control/*2652*<br>*Germany, ages 0-14 years*<br>Frequency matched conditional logistic regression	Matching on sex, date of birth, and district. Adjustment for age, sex, year of birth, degree of urbanization, and socioeconomic position
Shaw, 2004 (23)	Anti-asthmatics/*16*<br>*Maternal retrospective report*	Classification: ICD<br>ALL/*789*	Case-control/*1578*<br>*Canada, age 0-14 years*<br>Conditional logistic regression	Matching on sex and age at diagnosis. Adjustment for maternal age and education
Stålberg, 2010 (25)	Anti-asthmatics/*68*<br>*Routinely collected health data*	Classification: ICD<br>Brain tumor (ICD-7 code 193)/*512*	Case-control/*1037*<br>*Sweden, ages 0-15 years*<br>Logistic regression	Matching on sex and birth year. Adjustment for maternal age, parity, country of birth, and level of hospital (primary, secondary, or tertiary)

Reference	Exposure	Outcome	Materials and methods	
First author, year	Medication/*exposed*<br>*Ascertainment*<br>Exposure window: during pregnancy, unless otherwise stated	Classification system, type of cancer/*cases*	Design/*sample size*<br>*Study characteristics*<br>Statistical analysis	Method to account for confounding in the main analysis
Tegethoff, 2013 (58)	Asthma medications/*2510*<br>*Maternal prospective report*	Classification: ICD<br>Neoplasms/*721*	Cohort/*66,712*<br>*Denmark, ages 3-9 years*<br>Cox regression	Adjustment for maternal age, socioeconomic position, parity, general health, smoking, and child sex
Wen, 2002 (27)	Prescription cold or cough remedies in the year before pregnancy, through pregnancy and/or during breastfeeding/*95*<br>*Maternal retrospective report*	Classification system not stated<br>ALL/*1842*	Case-control/*3828*<br>*US, ages 0-14 years*<br>Conditional logistic regression	Matching on age, ethnicity, and telephone area.<br>Adjustment for parental income, education, ethnicity, smoking and alcohol use
FERTILITY MEDICATIONS				
Ajrouche, 2014 (59)	Any fertility treatment/*139,* Clomiphene citrate/*36,* Gonadotropins/*63,* Dopamine agonists/*6*<br>*Maternal retrospective report, validated in a sensitivity analysis assuming worst case differential misclassification*	Classification system not stated<br>Main outcome: Acute leukemias/*747*<br>Secondary outcomes: ALL/*636,* AML/*100*	Case-control/*2168*<br>*France, ESCALE, ages 0-15 years*<br>Unconditional logistic regression	Matching on age and sex.<br>Adjustment for age, sex, maternal age, birth order, maternal education or occupation
Bradbury & Jick, 2004 (60)	IVF/*176*<br>*Routinely collected health data*	Classification system not stated<br>Retinoblastoma/*24*	Cohort/*358,270*<br>*UK, age 0-5 years*<br>Descriptive statistics	No adjustment
Brinton, 2004 (61)	Any ovarian stimulation/*349,*<br>Clomiphene citrate/*181,* hCG/*184,* hMG/*49*<br>Each of the above ever before birth of index child. Also analysis by number of treatments.<br>Comparators are women with infertility. Some may be exposed to other fertility drugs<br>*Routinely collected health data*	Classification system not stated<br>Main outcome: Any cancer/*47*<br>Secondary outcomes:<br>Hematopoietic tumors (ALL, AML, HL, NHL)/*19*<br>Neural tumors (neuroblastomas, astrocytomas, medulloblastomas and other brain tumors)/*19*<br>Other/*9*	Case-cohort/915<br>*Denmark, ages 0-20 years*<br>Cox regression	Matching on maternal age and calendar year at entry into infertility cohort. Adjustment for maternal age at birth
Foix-L'Hélias, 2012 (62)	Any infertility treatment/*1575*<br>Ovarian stimulation/*837*<br>*Maternal retrospective report, validated in a sensitivity analysis assuming worst case differential misclassification*	Classification system not stated<br>Non-familial retinoblastoma/*244,* for the exposure "any fertility treatment" also by mutation	Case-cohort/*28,414*<br>*France, ages 0-5 years*<br>Logistic regression for main exposure, descriptive statistics for the exposure reported here	No adjustment in the analysis on medication use

Reference	Exposure	Outcome	Materials and methods	
First author, year	Medication/*exposed*<br>*Ascertainment*<br>Exposure window: during pregnancy, unless otherwise stated	Classification system, type of cancer/*cases*	Design/*sample size*<br>*Study characteristics*<br>Statistical analysis	Method to account for confounding in the main analysis
		status and by multifocality (yes/no)		
Hargreave, 2015 (63)	Any fertility medication/*794*, Clomiphene citrate/*443*, Gonadotropins/*375*, GnRH/*272*, hCG/*604*, Progesterone/*260*<br>Each of the above ever before birth of index child<br>Also analysis by number of treatments<br>Comparators were diagnosed with infertility<br>*Routinely collected health data*	Classification: ICCC<br>Main outcome:<br>Any cancer/*129*<br>Secondary outcomes:<br>Leukemias/*48*, lymphomas/*10*, SNS tumors/*13*, CNS tumors/*33*, other cancers/*25*	Case-cohort/*1418*<br>*Denmark, ages 0-19 years*<br>Cox regression	Matching on maternal age and calendar year at entry into infertility cohort
Hargreave, 2019 (64)	Any fertility medication/*89,334*, Clomiphene citrate/*33,835*, Gonadotropins/*57,136*, hCG/*68,181*, GnRH analogues/*38,653*, Progesterone/*41,628*, Estrogen/*16,948*<br>Uses disease comparators in a sensitivity analysis.<br>*Routinely collected health data*	Classification system not stated<br>Main outcome:<br>Any cancer/*2217*<br>Secondary outcomes:<br>Leukemias/*648*, lymphomas/*242*, SNS tumors/*130*, CNS tumors/*541*, other cancers/*661*	Cohort/*1,085,172*<br>*Denmark, ages 0-19, mean 11.3*<br>Cox regression	Adjustment for year of birth
Heck, 2012 (65)	Fertility enhancing drugs/*not reported*<br>*Routinely collected health data*	Classification system not stated<br>Retinoblastoma/*609*	Case-control/*209,660*<br>*US, APCC, ages 0-5 years*<br>Logistic regression	Matching on year of birth.<br>Adjustment for year of birth, paternal age, county of residence, and maternal place of birth
Heck, 2013 (66)	Fertility treatment/*not reported*<br>*Routinely collected health data*	Classification system not stated<br>Hepatoblastoma/*261*	Case-control/*218,538*<br>*California, ages 0-5 years*<br>Unconditional logistic regression	Matching on year of birth.<br>Adjustment for year of birth, maternal age, and maternal ethnicity
Heck, 2015 (9)	Fertility medication/*28*<br>*Maternal retrospective report*	Classification system not stated<br>Sporadic retinoblastoma, unilateral/*187*<br>Sporadic retinoblastoma, bilateral/*95*	Case-control/*426*<br>*US/Canada, ages 0-15 years*<br>Unconditional logistic regression	Matching on year of birth.<br>Adjustment for maternal ethnicity, education, income, maternal age, and smoking in pregnancy
Källen, 2009 (67)	IVF/*26692*<br>*Routinely collected health data*	Classification system not stated<br>Any cancer/*6458*	Cohort/*2,425,733*<br>*Sweden, ages 0-23 years*	Adjustment for year of birth

Reference	Exposure	Outcome	Materials and methods	
First author, year	Medication/*exposed*<br>*Ascertainment*<br>Exposure window: during pregnancy, unless otherwise stated	Classification system, type of cancer/*cases*	Design/*sample size*<br>*Study characteristics*<br>Statistical analysis	Method to account for confounding in the main analysis
		Lists the specific types of cancer observed in exposed children	Mantel-Haenszel, CI calculated by Miettinen's technique	
Klip, 2001 (68)	Any assisted reproduction or fertility drugs/*9479*<br>Comparators were women with subfertility diagnosis who conceived spontaneously<br>*Routinely collected health data*	Classification system not stated<br>Any cancer/*16*	Cohort/*17,000*<br>*The Netherlands (OMEGA), ages 0-15 years*<br>Cox regression	Adjustment for sex
Lerner-Geva, 2017 (69)	IVF/ICSI/*9042*<br>*Routinely collected health data*	Classification: ICCC<br>Main outcome: Any cancer/*382*<br>Secondary outcomes: Cancers by ICCC-3 groups: Leukemias, myeloproliferative diseases, and myelodysplastic diseases/*94*, lymphomas and reticuloendothelial neoplasms/*67*, CNS and miscellaneous intracranial and intraspinal neoplasms/*72*, neuroblastoma and other peripheral nervous cell tumors/*58*, retinoblastoma/*11*, renal tumors/*27*, hepatic tumors/*2*, malignant bone tumors/*10*,<br>soft tissue and other extraosseous sarcomas/*27*, germ cell tumors, trophoblastic tumors, and neoplasms of gonads/*2*, other malignant epithelial neoplasms and malignant melanomas/*9*, other and unspecified malignant neoplasms/*3*	Cohort/*220,805*<br>*Israel, ages 0-12 years*<br>Poisson regression	Adjustment for maternal age, education, ethnicity, child plurality, sex, birth weight, and congenital malformations
Mallol-Mesnard, 2008 (70)	Assisted reproduction/*93*<br>*Maternal retrospective report*	Classification: ICCC<br>Main outcome: Any CNS tumor/*209*	Case-control/*1890*<br>*France, ESCALE, ages 0-14 years*	Matching on age, sex, and number of siblings.<br>Adjustment for age and sex

Reference	Exposure	Outcome	Materials and methods	
First author, year	Medication/*exposed*<br>*Ascertainment*<br>Exposure window: during pregnancy, unless otherwise stated	Classification system, type of cancer/*cases*	Design/*sample size*<br>*Study characteristics*<br>Statistical analysis	Method to account for confounding in the main analysis
		Secondary outcomes: Ependymomas/*33*, embryonal tumors/*100*, astrocytomas/*26*, other gliomas/*45*	Unconditional logistic regression	
McCredie, 1994 (13)	Fertility drugs in the month before or during pregnancy/*5*<br>*Maternal retrospective report*	Classification: ICD<br>Brain or cranial nerves tumors/*82*	Case-control/*246*<br>*Australia, ages 0-14 years*<br>Conditional logistic regression	Matching on sex and age. Adjustment for parental education
McLaughlin, 2006 (71)	Presumptive fertility treatment (IVF, other fertility treatment, or triplet pregnancy)/*28*<br>*Routinely collected health data*	Classification system not stated<br>Hepatoblastoma/*58*	Case-control/*6114*<br>*US ages 0-5 years*<br>Not specified	Matching on birth year. Adjustment for birth year and birth weight
Munzer, 2008 (72)	Fertility treatment/*91*, Ovarian stimulation only/*46*<br>*Maternal retrospective report, validated in a sensitivity analysis restricted to women who had access to their children's health card during interview*	Classification: ICCC<br>Neuroblastoma/*191*, also neuroblastoma subgroups by MYCN oncogene amplification status	Case-control/*1872*<br>*France, ESCALE, 0-14 years*<br>Unconditional logistic regression	Matching on age and sex. Adjustment for matching factors
Olshan, 1999 (73)	Any fertility drug/*54*, Any ovulation-inducing drug/*49*, Clomiphene/*46*<br>Each of the above ever, in the 2-12 months prior to pregnancy, or 1 month prior to pregnancy<br>*Maternal retrospective report*	Classification system not stated<br>Neuroblastoma/*504*, also neuroblastoma subgroups by MYCN oncogene amplification status in an exploratory analysis	Case-control/*1008*<br>*US and Canada, ages 0-19 years*<br>Conditional logistic regression	Matching on date of birth. Adjustment for maternal ethnicity, education, and income
Petridou, 2012 (74)	IVF/*137*<br>*Maternal retrospective report, validated from medical record in Greece, routinely collected health data in Sweden*	Classification: ICD<br>Main outcome: Leukemias/*1334*<br>Secondary outcomes: ALL/*1095*, lymphoma/*348*	Case-control *in Greece, ages 0-14, pooled with* case-cohort *in Sweden, ages 0-12 years/8683*<br>Not specified how OR with 95% CI were estimated, but results from the two countries were combined using a fixed-effects model	Matching on age and sex. Adjustment for birth weight, birth order, maternal age, education, smoking during pregnancy, and plurality
Puumala, 2007 (75)	Infertility medication, ever/*32*<br>*Maternal retrospective report*	Classification system not stated<br>Main outcome: Leukemias/*158*<br>Secondary outcomes: ALL/*97*, AML/*61*	Case-control/*331*<br>*US, Children's Oncology Group, ages 0-19 years, all*	Matching on age. Adjustment for maternal age, ethnicity, education, and child sex

Reference	Exposure	Outcome	Materials and methods	
First author, year	Medication/*exposed*<br>*Ascertainment*<br>Exposure window: during pregnancy, unless otherwise stated	Classification system, type of cancer/*cases*	Design/*sample size*<br>*Study characteristics*<br>Statistical analysis	Method to account for confounding in the main analysis
			cases and controls have Down syndrome.<br>Unconditional logistic regression	
Puumala, 2010 (76)	Ovarian stimulating drugs, unclear whether ever or for index pregnancy/*34*<br>*Maternal retrospective report*	Classification system not stated<br>Main outcome: Infant leukemias/*443*<br>Secondary outcomes: ALL/*264*, AML/*172*, also analyzed by MLL gene translocation status	Case-control/*767*<br>*US, Children's Oncology Group, ages 0-1 years*<br>Logistic regression	Matching on year of birth and region of residence.<br>Adjustment for matching factors, maternal age, education, ethnicity, smoking during pregnancy, income, gestational age and birth weight
Puumala, 2011 (77)	Ovarian stimulating drugs in the year prior to or during index pregnancy/*16*<br>*Maternal retrospective report*	Classification system not stated<br>Main outcome: Germ cell tumors/*278*<br>Secondary outcomes: Gonadal germ cell tumors/*144*, non-gonadal germ cell tumors/*120*	Case-control/*700*<br>*US, Children's Oncology Group, ages 0-14 years*<br>Unconditional logistic regression	Matching on sex, age and telephone area code.<br>Adjustment for age, sex, gestational age, maternal age, ethnicity, education, and income
Puumala, 2012 (78)	Infertility medication, unclear whether ever or for index pregnancy/*79*<br>*Maternal retrospective report*	Classification system not stated<br>Hepatoblastoma/*383*	Case-control/*770*<br>*US, Children's Oncology Group, ages 0-5 years*<br>Logistic regression	Matching on birth weight, sex, year of birth, and region.<br>Adjustment for matching factors, maternal age, education, ethnicity, birth plurality, and gestational age
Reigstad, 2016 (79)	Assisted reproduction/*25,782*<br>*Routinely collected health data*	Classification: ICD<br>Main outcome: Any cancer/*4554*<br>Secondary outcomes: Main ICCC-3 diagnostic groups, and subgroups for leukemias, lymphomas and CNS tumors: ALL/*777*, AML/*178*, other leukemias/*74*, HL/*261*, NHL/*148*, astrocytoma/*368*, embryonal	Cohort/*1,628,658*<br>*Norway, ages 0-28 years, median follow-up 6.9 years in exposed, 13.7 years in unexposed*<br>Cox regression	Adjusted for calendar year at follow-up, region, maternal age, and birth order

Reference	Exposure	Outcome	Materials and methods	
First author, year	Medication/*exposed*<br>*Ascertainment*<br>Exposure window: during pregnancy, unless otherwise stated	Classification system, type of cancer/*cases*	Design/*sample size*<br>*Study characteristics*<br>Statistical analysis	Method to account for confounding in the main analysis
		CNS tumors/*179*, other gliomas/*105*, other CNS tumors/*133*, neuroblastoma/*188*, retinoblastoma/*91*, renal/*254*, hepatic/*110*, soft tissue/*312*, other/*491*		
Ross, 2003 (19)	Clomiphene/*10*<br>*Routinely collected health data*	Classification system not stated<br>IL/*243*, ALL/*157*, AML/*77*	Case-control/*636*<br>*US, ages 0-18 months*<br>Conditional logistic regression	Matching on birth year and telephone area. Adjustment for maternal age, education and income
Rudant, 2013 (80)	Ovulation induction drugs (without in vitro fertilization or artificial insemination)/*79*<br>*Maternal retrospective report*	Classification system not stated<br>Main outcome: Acute leukemias/*764*<br>Secondary outcomes: ALL/*648*, AML/*101*	Case-control /*2445*<br>*France, ages 0-15 years*<br>Unconditional logistic regression	Matching on sex and age. Adjustment for sex, age, parental occupation, and maternal age
Schüz, 1999 (81)	Hormonal treatment for infertility, unclear whether ever or for index pregnancy/*225*<br>*Maternal retrospective report from questionnaire data validated through phone interview*	Classification system not stated<br>Main outcomes: Acute leukemias/*1184*, NHL/*234*, CNS tumors/*399*, neuroblastomas/*160*, nephroblastomas/*147*, bone tumors/*97*, soft tissue sarcomas/*137*<br>Secondary outcomes: ALL/*1037*, common-ALL/*686*, pre-B-ALL/*121*, T-ALL/*99*, ANLL/*147*	Case-control/*4946*<br>*Germany, ages 0-14 years*<br>Frequency matched conditional logistic regression	Matching on sex, date of birth, and district. Adjustment for age, sex, year of birth, vicinity to nuclear installation, degree of urbanization, and socioeconomic position
Schüz, 2001 (82)	Hormonal treatment for infertility, unclear whether ever or for index pregnancy/*90*<br>*Maternal retrospective report from questionnaire data validated through phone interview*	Classification: International Neuroblastoma Staging System<br>Neuroblastoma/*166*	Case-control/*1866*<br>*Germany, ages 0-7 years.*<br>Frequency matched conditional logistic regression	Matching on sex, date of birth, and district. Adjustment for age, sex, year of birth, degree of urbanization, and socioeconomic position
Spector, 2019 (83)	IVF/*275,686*<br>*Routinely collected health data*	Classification: ICCC<br>Main outcome: Any cancer/*2363*	Cohort/*2,542,533*<br>*US, ages 0-10 years*	Adjustment for sex, plurality, maternal education, maternal

Reference	Exposure	Outcome	Materials and methods	
First author, year	Medication/*exposed*<br>*Ascertainment*<br>Exposure window: during pregnancy, unless otherwise stated	Classification system, type of cancer/*cases*	Design/*sample size*<br>*Study characteristics*<br>Statistical analysis	Method to account for confounding in the main analysis
		Secondary outcomes: ICCC3 subgroup if more than 10 exposed cases, otherwise ICCC3 group: ALL/*606*, AML/*121*, lymphomas/*161*, astrocytomas/*231*, ependymomas/*53*, intracranial embryonal tumors/*96*, neuroblastomas/*307*, retinoblastomas/*141*, renal/*214*, hepatic/*83*, soft tissue sarcomas/*115*, germ cell/*54*, embryonal/*877*	Cox regression	ethnicity, maternal age, state of birth
Sundh, 2014 (84)	Assisted reproductive technology including IVF, ICSI and frozen embryo transfer/*91,796*<br>*Routinely collected health data*	Classification: ICCC, based on ICD-codes.<br>Main outcome: Any cancer/*819*<br>Secondary outcomes: ICCC-3 groups: Leukemias/*278*, lymphomas/*52*, CNS tumors/*156*, neuroblastomas and peripheral nerve cell tumors/*48*, retinoblastomas/*35*, renal tumors/*51*, hepatic tumors/*10*, malignant bone tumors/*35*, soft tissue/extraosseous sarcomas/*37*, germ cell tumors/*21*, other malignant epithelial neoplasms/*40*, other/not specified malignant neoplasms/*56*	Cohort/*450,215*<br>*Nordic countries (Sweden, Denmark, Finland and Norway), mean length of follow up 9.5 years for all children*<br>Cox regression	Adjustment for country, maternal age, parity, sex, gestational age, birth defects and chromosomal aberrations
Wainstock, 2017 (85)	Fertility treatments: IVF/*2603*, ovulation induction/*1172*<br>*Maternal prospective report, validated in medical records*	Classification: ICD<br>Any malignant neoplasm/*1498*	Cohort/*242,187*<br>*Israel, children born between 1991 and 2013*	Adjusted for maternal age, birth weight, preterm birth, pregnancy-related hypertensive disorders, pre-

Reference	Exposure	Outcome	Materials and methods	
First author, year	Medication/*exposed*<br>*Ascertainment*<br>Exposure window: during pregnancy, unless otherwise stated	Classification system, type of cancer/*cases*	Design/*sample size*<br>*Study characteristics*<br>Statistical analysis	Method to account for confounding in the main analysis
			Kaplan-Meier survival curves and Cox-Mantel log rang test.	gestational diabetes, gestational diabetes
HIV-MEDICATIONS				
Benhammou, 2008 (86)	Zidovudine and lamivudine/*4752*<br>Didanosine and other non-lamivudine molecules/*874*, Didanosine and lamivudine/*365*, Other combinations/*715*<br>Each of the above compared to zidovudine monotherapy<br>*Routinely collected health data*	Classification system not stated<br>Any cancer/*10*	Cohort/*8853*<br>*France, Enquête Périnatale Française, ages 0-5 years*<br>Cox regression	Adjustment for prematurity
Hleyhel, 2016 (87)	Zidovudine and lamivudine/*7533*<br>Abacavir/tenofovir and emtricitabine/lamivudine/*3239*,Stavudine/zalcitabine alone or in combination with other molecules except didanosine/*649*, Didanosine alone or in combination with other molecules/*1461*<br>Each of the above compared to zidovudine monotherapy<br>Also analysis by duration<br>*Routinely collected health data*	Classification system not stated<br>Any cancer/*21*	Cohort/*15,163*<br>*France, Enquête Périnatale Française, 0-15 years*<br>Cox regression	Adjustment for prematurity, peripartum zidovudine infusion and postnatal HIV medication to the child
IMMUNOSUPPRESSANTS/ANTINEOPLASTIC AGENTS				
Chambers, 2019 (88)	Adalimumab /*257*<br>Both disease and population comparators<br>*Maternal prospective report*	Classification system not stated<br>Any cancer/*0*	Cohort/*602*<br>*US and Canada, MotherToBaby/OTIS, age 0-1 year*<br>Descriptive statistics for the cancer outcome	No adjustment for this outcome
Chaparro, 2018 (89)	Anti-TNFα/*388*<br>Disease comparator<br>*Routinely collected health data*	Classification system not stated<br>Any cancer/*0*	Cohort/*841*<br>*Europe, TEDDY, ages 0-15 years*<br>Descriptive statistics for the cancer outcome	No adjustment for this outcome

Reference	Exposure	Outcome	Materials and methods	
First author, year	Medication/*exposed*<br>*Ascertainment*<br>Exposure window: during pregnancy, unless otherwise stated	Classification system, type of cancer/*cases*	Design/*sample size*<br>*Study characteristics*<br>Statistical analysis	Method to account for confounding in the main analysis
Green, 1997 (90)	Cancer treatment any time before pregnancy/*92*<br>Comparators are children born to untreated wives of treated men.<br>*Routinely collected health data*	Classification system not stated<br>Any cancer/*0*	Cohort/*153*<br>*US, ages not reported*<br>Descriptive statistics for the comparison we report here	No adjustment for this outcome
Li, 1979 (91)	Cancer treatment any time before pregnancy/*159*<br>Comparators are cousins of exposed<br>*Routinely collected health data*	Classification system not stated<br>Any cancer/*2*	Cohort/*571*<br>*US, ages 0-10 years*<br>Descriptive statistics	No adjustment for this outcome
Ognjanovic, 2009 (30)	Steroids or immunosuppressants/*15*<br>*Maternal retrospective report*	Classification system not stated<br>Main outcome: Leukemias/*158*<br>Secondary outcomes: ALL/*97*, AML/*61*	Case-control/*331*<br>*US, Children's Oncology Group, ages 0-19 years, all cases and controls have Down syndrome*<br>Descriptive statistics	Matching on age and sex
Ross, 2003 (19)	Hydrocortisone/*21*<br>*Routinely collected health data*	Classification system not stated<br>IL/*243*, ALL/*157*, AML/*77*	Case-control/*636*<br>*US, ages 0-18 months*<br>Conditional logistic regression	Matching on birth year and telephone area, adjustment for maternal age, education and income
Salonen, 1976 (20)	Glucocorticoids/*2*<br>*Routinely collected health data*	Classification: ICD<br>Main outcomes: Leukemias/*373*, brain tumors/*245*, other tumors/*354*<br>Secondary outcomes: Subgroups of the "other tumors"-category; kidney tumors/*96*, eye tumors/*37*, bone tumors/*56* (only reported for most commonly used medications)	Case-control/*1944*<br>*Finland, ages 0-14 years*<br>MacNemar's test, descriptive for rarely used medications	Matching on date of birth
Shaw, 2004 (23)	Immunosuppressants/*19*<br>*Maternal retrospective report*	Classification: ICD<br>ALL/*789*	Case-control/*1578*<br>*Canada, age 0-14 years*<br>Conditional logistic regression	Matching on sex and age at diagnosis, adjustment for maternal age and education

Reference	Exposure	Outcome	Materials and methods	
First author, year	Medication/*exposed*<br>*Ascertainment*<br>Exposure window: during pregnancy, unless otherwise stated	Classification system, type of cancer/*cases*	Design/*sample size*<br>*Study characteristics*<br>Statistical analysis	Method to account for confounding in the main analysis
Wen, 2002 (27)	Immunosuppressants or steroids in the year before pregnancy, through pregnancy and/or during breastfeeding/*28*<br>*Maternal retrospective report*	Classification system not stated<br>ALL/*1842*	Case-control/*3828*<br>*US, ages 0-14 years*<br>Conditional logistic regression	Matching on age, ethnicity, and telephone area, adjustment for parental income, education, ethnicity, smoking and alcohol use
LAXATIVES/ANTIDIARRHEALS				
Birch, 1990 (92)	Laxatives/*not reported*<br>*Maternal retrospective report, validated in prescription records*	Classification: ICD<br>CNS tumors/*78*	Case-control/*234*<br>*UK, IRESCC, ages 0-15 years*<br>Mantel-Haenszel	Matching on age and sex
Gilman, 1989 (6)	Laxatives/*47*<br>*Maternal retrospective report and medical records. Children are considered exposed if medication use is present in either source*	Classification system not stated<br>Any cancer/*8059*	Case-control/*16,118*<br>*UK, OSCC, age not specified, only that cases are childhood deaths from cancer*<br>Conditional logistic regression	Matching on date of birth, sex and district, adjustment for abdominal x-rays in pregnancy, birth order among live born siblings, maternal age, and socioeconomic position
Hartley, 1988 (8)	Laxatives/*not reported*<br>*Maternal retrospective report, validated in prescription data*	Classification system not stated<br>Bone or soft tissue sarcomas/*73*	Case-control/*219*<br>*UK, IRESCC, ages 0-15 years*<br>Mantel-Haenszel, 95% CI by Cornfield's formula	Matching. Matching factors not reported, but they refer to a paper where methods are described (age and sex)
Ross, 1996 (57)	Laxatives/*13*<br>Antidiarrheals/*10*<br>*Maternal retrospective report*	Classification system not stated<br>IL/*84*	Case-control/*181*<br>*US, ages 0-1 year*<br>Conditional logistic regression	Matching on year of birth, geography and ethnicity, adjustment for maternal education
Ross, 2003 (19)	Loperamide/*7*<br>*Routinely collected health data*	Classification system not stated<br>IL/*243*, ALL/*157*, AML/*77*	Case-control/*636*<br>*US, ages 0-18 months*<br>Conditional logistic regression	Matching on birth year and telephone area, adjustment for maternal age, education and income
Wen, 2002 (27)	Laxatives or antidiarrheal agents in the year before pregnancy, through pregnancy and/or during breastfeeding/*75*<br>*Maternal retrospective report*	Classification system not stated<br>ALL/*1842*	Case-control/*3828*<br>*US, ages 0-14 years*<br>Conditional logistic regression	Matching on age, ethnicity, and telephone area, adjustment for parental

Reference	Exposure	Outcome	Materials and methods	
First author, year	Medication/*exposed*<br>*Ascertainment*<br>Exposure window: during pregnancy, unless otherwise stated	Classification system, type of cancer/*cases*	Design/*sample size*<br>*Study characteristics*<br>Statistical analysis	Method to account for confounding in the main analysis
				income, education, ethnicity, smoking and alcohol use
ORAL CONTRACEPTIVES/SEX HORMONES				
Ajrouche, 2014 (59)	Oral contraceptives/*116*, 1st generation/*1*, 2nd generation/*66*, 3rd generation*/12*, 4th generation*/1*, Other oral contraceptive*/10*<br>Also analysis by dose<br>*Maternal retrospective report, validated in a sensitivity analysis assuming worst case differential misclassification*	Classification system not stated<br>Main outcome: Acute leukemias/*747*<br>Secondary outcomes: ALL/*636*, AML/*100*	Case-control/*2168*<br>*France, ESCALE, ages 0-15 years*<br>Unconditional logistic regression	Matching on age and sex. Adjustment for age, sex, birth order, maternal age, education or occupation
Birch, 1990 (92)	Oral contraceptives any time before pregnancy/*not reported*<br>*Maternal retrospective report, validated in prescription records*	Classification: ICD<br>CNS tumors/*78*	Case-control/*234*<br>*UK, IRESCC, ages 0-15 years*<br>Mantel-Haenszel	Matching on age and sex
Bonaventure, 2015 (1)	Any sex hormones (ATC group G)/*426*<br>*Routinely collected health data*	Classification: ICD<br>ALL/*725*, AML/*128*, NHL/*83*, HL/*31*, astrocytoma/*100*, medulloblastoma/*48*, rhabdomyosarcoma/*54*, Ewings sarcoma/*20*, neuroblastoma/*78*, nephroblasoma/*60*	Case-control/*4122*<br>*UK, UKCCS, mean age 5.8 (max age 15 years)*<br>Unconditional logistic regression	Matching on sex, month and year of birth, and region of residence. Adjustment for year of birth
Buckley, 1989 (33)	Oral contraceptives in the year before pregnancy or during pregnancy/*39*<br>*Maternal retrospective report*	Classification system not stated<br>Hepatoblastoma/*75*	Case-control/*150*<br>*US and Canada, CCSG, upper age limit not stated, but the majority of children were under 2 years of age*<br>Conditional logistic regression	Matching on age
Bunin, 1987 (38)	Sex hormones in the year prior to birth/*15*<br>*Maternal retrospective report*	Classification system not stated<br>Nephroblastoma/*88*, also stratified by whether the case was genetic	Case-control/*176*<br>*US, ages 0-15 years*<br>Conditional logistic regression	Matching on telephone area code, year of birth and ethnicity. Adjustment for demographic characteristics (not specified further)

Reference	Exposure	Outcome	Materials and methods	
First author, year	Medication/*exposed*<br>*Ascertainment*<br>Exposure window: during pregnancy, unless otherwise stated	Classification system, type of cancer/*cases*	Design/*sample size*<br>*Study characteristics*<br>Statistical analysis	Method to account for confounding in the main analysis
Bunin, 1989 (34)	Oral contraceptives at conception/*5*<br>*Maternal retrospective report*	Classification system not stated<br>Nonheritable retinoblastoma/*115*, sporadic heritable retinoblastoma (bilateral cases or unilateral cases with 13q chromosomal deletion)/*67*<br>Cases with family history were excluded	Case-control/*383*<br>*US and Canada, CCSG, upper age limit not stated*<br>Conditional logistic regression	Matching on telephone area code, year of birth and ethnicity. Adjustment for paternal education
Bunin, 1994 (2)	Oral contraceptives /*20*<br>*Maternal retrospective report*	Classification system not stated<br>Astrocytoma/*155*, PNET/*166*	Case-control/*332*<br>*US and Canada, Children's Cancer Group, ages 0-5 years*<br>Conditional logistic regression	Matching on telephone area code, year of birth and ethnicity. Adjustment for income (only for astrocytoma)
Cardy, 2006 (35)	Sex hormones/*179*<br>*Maternal retrospective report*	Classification: ICD-O<br>Main outcome:<br>Any brain tumor/*1218*<br>Secondary outcomes:<br>Astroglial/*623*, PNET/*259*, other glial/*327* by morphological codes	Case-control/*3441*<br>*Europe, North America, Australia, SEARCH, upper age limit 19 years*<br>Unconditional logistic regression	Matching, not further specified. Adjustment for center, age, sex, year of birth, and parental education. Vitamin supplementation (unclear whether in mothers or children) was considered as both a confounder and effect modifier
Cook, 2004 (4)	Ethinyl estradiol/*27*, Mestranol/*17*, Norethindrone/*31*<br>Each of the above during pregnancy or breastfeeding<br>*Maternal retrospective report*	Classification system not stated<br>Neuroblastoma/*504*	Case-control/*1008*<br>*US and Canada, Children's Cancer Group, upper age limit 18 years*<br>Conditional logistic regression	Matching on age. Adjustment for maternal age, education, and ethnicity
Erjaee, 2017 (93)	Oral contraceptives in the first 2 months of pregnancy/*20*<br>*Maternal retrospective report, validated in medical records*	Classification system not stated<br>Any cancer/*300*	Case-control/*900*<br>*Iran, ages 0-17 years*<br>Logistic regression	Matching on age and sex. Adjustment for paternal education, family history of cancer, infection during pregnancy, hyperemesis gravidarum, parental

Reference	Exposure	Outcome	Materials and methods	
First author, year	Medication/*exposed*<br>*Ascertainment*<br>Exposure window: during pregnancy, unless otherwise stated	Classification system, type of cancer/*cases*	Design/*sample size*<br>*Study characteristics*<br>Statistical analysis	Method to account for confounding in the main analysis
				smoking, radiation during pregnancy, child allergy, and environmental exposures for which it is unclear whether they take place before or after birth, including: use of wood burners at home, residence near power line, contact with insecticides, and contact with domestic animals
Gholami, 2011 (94)	Oral contraceptives any time before pregnancy/*108*<br>*Maternal retrospective report*	Classification system not stated<br>Acute leukemia/*130*	Case-control/*260*<br>*Iran, ages 0-15 years, study reported results for both population- and hospital controls, here we report the population controls*<br>Logistic regression	Matching on age and sex. Adjustment for maternal education and parental relationship status
Gilman, 1989 (6)	Sex hormones/*357*<br>*Maternal retrospective report and medical records. Children are considered exposed if medication use is present in either source*	Classification system not stated<br>Any cancer/*8059*	Case-control/*16,118*<br>*UK, OSCC, age not specified, only that cases are childhood deaths from cancer*<br>Conditional logistic regression	Matching on date of birth, sex and district. Adjustment for abdominal x-rays in pregnancy, birth order among live born siblings, maternal age, and socioeconomic position
Gold, 1979 (95)	Oral contraceptives any time before birth/*not reported*<br>Sex hormones/*not reported*<br>*Maternal retrospective report, uses cancer controls in addition to population controls, where the cancer controls are expected to be influenced by the same recall factors as cases*	Classification system not stated<br>Brain tumors/*73*	Case-control/*134*<br>*US, two control groups: healthy and cancer controls, ages 0-19 years*<br>Matched pairs OR, binomial test, exact 95% CI calculated based on the binomial distribution	Matching on ethnicity, sex, and year of birth

Reference	Exposure	Outcome	Materials and methods	
First author, year	Medication/*exposed*<br>*Ascertainment*<br>Exposure window: during pregnancy, unless otherwise stated	Classification system, type of cancer/*cases*	Design/*sample size*<br>*Study characteristics*<br>Statistical analysis	Method to account for confounding in the main analysis
Golding, 1990 (96)	Oral contraceptives in the 18 months before conception/*37*<br>*Maternal prospective report*	Classification system not stated<br>Any cancer/*33*	Case-cohort/*132*<br>*UK, ages 0-10 years*<br>Analysis after Pike and Morrow	Matching on maternal age, parity, marital status, paternal occupation, and plurality, each determined at delivery
Grufferman, 1982 (7)	Oral contraceptives or other birth control methods/*31*, Drugs for menstrual disorders or infertility/*7*<br>Each of the above 1 year prior to pregnancy or during pregnancy<br>*Maternal retrospective report*	Classification system not stated<br>Rhabdomyosarcoma/*33*	Case-control/*132*<br>*US, ages 0-14 years*<br>"Unmatched methods", not specified further	Matching on age, sex, and ethnicity
Hargreave, 2018 (97)	Any hormonal contraception/*914866*, Combined oral contraceptives/*781,833*, Combined non-oral contraceptives/*29,996*,<br>Progestin-only oral contraceptives/*77,152*,<br>Progestin-only non-oral contraception/*17,129*,<br>Emergency contraception/*131,503*<br>Each of the above >3 months prior to pregnancy, or ≤3 months prior or during pregnancy<br>*Routinely collected health data*	Classification: ICCC<br>Main outcome: Any leukemias/*606*<br>Secondary outcomes: Lymphoid leukemias/*465*, non-lymphoid leukemias/*141*, and genetic subtypes of leukemia	Cohort/*1,185,157*<br>*Denmark, ages 0-18 years*<br>Cox regression	Adjustment for year of birth, maternal age and infertility.
Hartley, 1988 (8)	Any sex hormones, including oral contraceptives/*not reported*<br>*Maternal retrospective report, validated in prescription data*	Classification system not stated<br>Bone or soft tissue sarcomas/*73*	Case-control/*219*<br>*UK, IRESCC, ages 0-15 years*<br>Mantel-Haenszel, 95% CI by Cornfield's formula	Matching. Matching factors not reported, but they refer to a paper where methods are described (age and sex)
Heck, 2015 (9)	Oral contraceptives/*131*, Non-oral hormonal contraceptives/*29*,<br>Any hormonal contraceptive/*154*<br>Each of the above in the year before pregnancy<br>*Maternal retrospective report*	Classification system not stated<br>Sporadic retinoblastoma, unilateral/*187*, sporadic retinoblastoma, bilateral/*95*	Case-control/*426*<br>*US/Canada, ages 0-15 years*<br>Unconditional logistic regression	Matching on year of birth. Adjustment for maternal ethnicity, education, income, age, and smoking in pregnancy
Herbst, 1971 (98)	Diethylstilbestrol/*7*<br>*Maternal retrospective report*	Classification system not stated<br>Vaginal adenocarcinoma/*8*	Case-control/*40*<br>*Boston, ages 14-22, two out of eight cases were >19 years*<br>Matched control method from Pike & Morrow	Matching on date of birth and type of delivery service (ward or private)

Reference	Exposure	Outcome	Materials and methods	
First author, year	Medication/*exposed*<br>*Ascertainment*<br>Exposure window: during pregnancy, unless otherwise stated	Classification system, type of cancer/*cases*	Design/*sample size*<br>*Study characteristics*<br>Statistical analysis	Method to account for confounding in the main analysis
Johnston, 1986 (99)	Oral contraceptives any time before pregnancy/*68,*<br>Oral contraceptives in the year prior to pregnancy/*54*<br>*Maternal retrospective report, validated in medical records*	Classification: According to a 1981 paper by Marsden et al. *Germ cell tumors of childhood* (higher level of detail than the ICCC)<br>Germ cell tumors/*41*	Case-control/*123*<br>*UK, IRESCC, ages 0-15 years*<br>Descriptive statistics	Matching. Matching factors not reported, but they refer to a paper where methods are described (age and sex)
Kinnier-Wilson, 1981 (100)	Any hormones/*444*<br>*Maternal retrospective report, asks antenatal clinics to verify exposure*	Classification system not stated<br>Any cancers/*2287*	Case-control/*4317*<br>*UK, age not specified, only that cases are childhood deaths from cancer, results are stratified by x-ray exposure, we report results for x-ray unexposed*<br>Mantel-Haenszel	Matching, unclear on which factors, stratified on prenatal x-ray exposure
Kramer, 1987 (11)	Sex hormones 3 months prior to pregnancy/*not reported*<br>Sex hormones/*not reported*<br>*Maternal retrospective report, validated in medical records for a subsample. Validity compared between cases and controls*	Classification system not stated<br>Neuroblastoma/*104*	Case-control/*205*<br>*US, median age 1 year*<br>90% CI calculated by Miettinen's technique	Matching on telephone area code, ethnicity, and year of birth
Kwan, 2007 (44)	Oral contraceptives 3 months before and/or during pregnancy/*136*<br>*Maternal retrospective report*	Classification: ICD-O<br>Main outcome: Leukemia/*365*<br>Secondary outcome: ALL/*311*	Case-control/*825*<br>*US, NCCLS, ages 0-14 years*<br>Conditional logistic regression	Matching on date of birth, sex, ethnicity (of child and mother), and for part of the recruitment also county of residence at birth. Adjustment for maternal age, income, education
Linet, 1996 (101)	Oral contraceptives >3 months prior to pregnancy/*105*<br>Oral contraceptives ≤3 months prior to pregnancy/*98*<br>*Routinely collected health data*	Classification: The American Cancer Society Revision of the World Health Organization Classification of Brain Tumors for childhood brain tumors	Case-control/*624*<br>*Sweden, ages 0-17 years*<br>Conditional logistic regression	Matching on sex, birth year and month. Adjustment for matching factors, gestational age and maternal narcotic use

Reference	Exposure	Outcome	Materials and methods	
First author, year	Medication/*exposed*<br>*Ascertainment*<br>Exposure window: during pregnancy, unless otherwise stated	Classification system, type of cancer/*cases*	Design/*sample size*<br>*Study characteristics*<br>Statistical analysis	Method to account for confounding in the main analysis
		Main outcome: Any brain tumor/*104*<br>Secondary outcomes: Low grade astrocytoma/*37*, high grade astrocytoma/*7*, medulloblastoma/*17*, *e*pendymoma/*15*, others/*27*		
Lupo, 2014 (102)	Any birth control (unspecified) any time before pregnancy/*463*<br>Any birth control (unspecified) at conception/*22*<br>*Maternal retrospective report*	Classification system not stated<br>Main outcome: Rhabdomyosarcoma/*322*<br>Secondary outcomes: Embryonal rhabdomyosarcoma/*215*, alveolar rhabdomyosarcoma/*66*	Case-control/*644*<br>*US, Children's Oncology Group, ages 0-20 years*<br>Conditional logistic regression	Matching on ethnicity, sex, and age. Adjustment for matching factors, parental education and income
McCredie, 1994 (13)	Oral contraceptives/*25*<br>Drugs to prevent miscarriage/*8*<br>Each of the above in the month before or during pregnancy<br>*Maternal retrospective report*	Classification: ICD<br>Brain or cranial nerves tumors/*82*	Case-control/*246*<br>*Australia, ages 0-14 years*<br>Conditional logistic regression	Matching on sex and age. Adjustment for parental education
McKinney, 1987 (14)	Hormones/*not reported*<br>Hormonal confirmation of pregnancy/*not reported*<br>*Maternal retrospective report and medical records, findings from the two data sources are reported separately*	Classification system not stated<br>Main outcome: Leukemias/lymphomas/*234*<br>Secondary outcomes: Leukemias/*171*<br>Only reported separately for significant associations	Case-control/*702*<br>*UK, IRESCC, ages 0-15 years*<br>Mantel-Haenszel	Matching on age and sex
Michalek, 1996 (29)	Sex hormones, any/*24*<br>Sex hormones by indication:<br>Infertility/*6*, Vaginal bleeding/*8*,<br>To maintain pregnancy/*13*,<br>Previous miscarriage/*3*<br>*Maternal retrospective report*	Classification system not stated<br>Neuroblastoma/*183*	Case-control/*555*<br>*US, ages 0-14 years*<br>Unconditional logistic regression	Matching on year of birth
Olshan, 1999 (73)	Oral contraceptives/*278*	Classification system not stated	Case-control/*1008*	Matching on date of birth. Adjustment for maternal

Reference	Exposure	Outcome	Materials and methods	
First author, year	Medication/*exposed*<br>*Ascertainment*<br>Exposure window: during pregnancy, unless otherwise stated	Classification system, type of cancer/*cases*	Design/*sample size*<br>*Study characteristics*<br>Statistical analysis	Method to account for confounding in the main analysis
	Ever, in the 2-12 months prior to pregnancy, in the month prior to pregnancy, or during the first trimester<br>*Maternal retrospective report*	Neuroblastoma/*504*, also neuroblastoma subgroups by MYCN oncogene amplification status in an exploratory analysis	*US and Canada, ages 0-19 years*<br>Conditional logistic regression	ethnicity, education, and income
Operskalski, 1987 (103)	Estrogen in the year before or during pregnancy/*not reported*<br>*Maternal retrospective report*	Classification system not stated<br>Osteosarcoma/*64*	Case-control/*188*<br>*US, ages 0-25 years (88% of the population were under 20 years old)*<br>Breslow & Day's method	Matching on ethnicity, sex, and birth year
Ou Shu, 2002 (104)	Oral contraceptive/*126*, Hormone use other than oral contraceptives (unspecified) in the year prior to pregnancy/*173*, Hormone use other than oral contraceptives (unspecified)/*87*<br>*Maternal retrospective report*	Classification system not stated<br>Main outcome: ALL/*1842*<br>Secondary outcomes: T-cell ALL/*183*, early pre-B-cell ALL/*893*, pre-B-cell ALL/*233*	Case-control/*3828*<br>*US, ages 0-14 years*<br>Conditional logistic regression	Matching on age, ethnicity, telephone area code, and for T-cell ALL also sex. Adjustment for maternal education, ethnicity, age, and income
Pombo de Oliveira, 2006 (17)	Abortive drugs/*130*, Misoprostol/*13*, Hormones (Oral contraceptives, antiabortive progesterone treatment, and thyroid hormones)/*22*<br>Each of the above 3 months before pregnancy, during pregnancy, and/or during breastfeeding<br>*Maternal retrospective report*	Classification system not stated<br>Infant acute leukemias/*202*, also analyzed by MLL gene translocation status	Case-control/*642*<br>*Brazil, ages 0-21 months*<br>Unconditional logistic regression	Matching on age and region. Adjustment for region, sex, birth weight, maternal age, and income
Puumala, 2007 (75)	Ever use of oral contraceptives/*252*, Ever use of non-oral hormonal contraceptives/*20*<br>*Maternal retrospective report*	Classification system not stated<br>Main outcome: Leukemias/*158*<br>Secondary outcomes: ALL/*97*, AML/*61*	Case-control/*331*<br>*US, Children's Oncology Group, ages 0-19 years, all cases and controls have Down syndrome.*<br>Unconditional logistic regression	Matching on age. Adjustment for maternal age, ethnicity, education, and child sex
Robison, 1989 (37)	Oral contraceptives/*130*<br>Hormones (hormones of any sort, such as female hormones, fertility drugs, cortisone, insulin or thyroid hormones)/*23*	Classification system not stated<br>ANLL/*204*	Case-control/*408*<br>*US, ages 0-18 years*<br>Conditional logistic regression	Matching on date of birth, ethnicity, and telephone area code, stated that they adjust for confounders, but these are not specified

Reference<br><br>First author, year	Exposure<br><br>Medication/*exposed*<br>*Ascertainment*<br>Exposure window: during pregnancy, unless otherwise stated	Outcome<br><br>Classification system, type of cancer/*cases*	Materials and methods<br><br>Design/*sample size*<br>*Study characteristics*<br>Statistical analysis	 <br>Method to account for confounding in the main analysis
	Each of the above in the year before pregnancy, through pregnancy and/or during breastfeeding<br>Also analyses by duration and frequency<br>*Maternal retrospective report, validated by comparisons to associations in four other independent case-control studies where cases of different cancers did not report more medication use than controls*			
Salonen, 1976 (20)	Estrogen/*9*<br>Progesterone/*37*<br>*Routinely collected health data*	Classification: ICD<br>Main outcomes: Leukemias/*373*, brain tumors/*245*, other tumors/*354*<br>Secondary outcomes: Subgroups of the “other tumors”-category; kidney tumors/*96*, eye tumors/*37*, bone tumors/*56* (only reported for most commonly used medications)	Case-control/*1944*<br>*Finland, ages 0-14 years*<br>MacNemar’s test, descriptive for rarely used medications	Matching on date of birth
Schüz, 2001 (82)	Oral contraceptives or sex hormones/*38*<br>*Maternal retrospective report from questionnaire data validated through phone interview*	Classification: International Neuroblastoma Staging System<br>Neuroblastoma/*163*	Case-control/*1860*<br>*Germany, ages 0-7 years*<br>Frequency matched conditional logistic regression	Matching on sex, date of birth, and district. Adjustment for age, sex, year of birth, degree of urbanization, and socioeconomic position
Shankar, 2006 (105)	Female hormones six months before pregnancy until delivery, according to self-report/*53*, according to medical records/*123*<br>*Maternal retrospective report and medical records, findings from the two data sources are reported separately*	Classification system not stated<br>Germ cell tumors of all other sites than the brain/*278*	Case-control/*701*<br>*US, Children’s Oncology Group, ages 0 to 14 years*<br>Unconditional logistic regression	Matching on age, sex, and state. Adjustment for matching factors, maternal ethnicity, education and income
Sharpe & Franco, 1996 (22)	Oral contraceptives/*30*<br>*Maternal retrospective report*	Classification system not stated<br>Nephroblastoma/*109*	Case-control/*327*<br>*Brazil, age range not reported, mean age 3 years and 5 months*	Matching on age, sex, and interviewer, stratified on socioeconomic position

Reference	Exposure	Outcome	Materials and methods	
First author, year	Medication/*exposed*<br>*Ascertainment*<br>Exposure window: during pregnancy, unless otherwise stated	Classification system, type of cancer/*cases*	Design/*sample size*<br>*Study characteristics*<br>Statistical analysis	Method to account for confounding in the main analysis
			Logistic regression	
Shaw, 2004 (23)	Oral contraceptive/*19*<br>*Maternal retrospective report*	Classification: ICD<br>ALL/*789*	Case-control/*1578*<br>*Canada, age 0-14 years*<br>Conditional logistic regression	Matching on sex and age at diagnosis. Adjustment for maternal age and education
Shu, 1995 (24)	Birth control pills/*27*<br>*Maternal retrospective report*	Classification system not stated<br>Malignant germ-cell tumors/*105*	Case-control/*744*<br>*US and Canada, CCG (Children's Cancer Group), ages 0-15 years*<br>Unconditional logistic regression	Matching on telephone area code. Adjustment for child age, sex, gestational age, number of siblings, maternal education, and smoking
Stewart, 1958 (106)	Chemical contraceptives before birth of the child (ever before or during pregnancy)/*27*<br>*Maternal retrospective report, validated through a comparison of data completeness for sensitive questions between cases and controls*	Classification system not stated<br>Leukemia/*677*, other cancers/*739*	Case-control/*2301*<br>*England and Wales, ages 0-9 years*<br>Comparison of matched children, not specified further	Matched on age, sex and locality, or chosen at random. Not clear how many were chosen in each fashion
Stålberg, 2010 (25)	Hormones (unspecified)/*<10*<br>*Routinely collected health data*	Classification: ICD<br>Brain tumor (ICD-7 code 193)/*512*	Case-control/*1037*<br>*Sweden, ages 0-15 years*<br>Logistic regression	Matching on sex and birth year. Adjustment for maternal age, parity, country of birth, and level of hospital (primary, secondary, or tertiary)
Tournaire, 2016 (107)	Maternal in-utero exposure to diethylstilbestrol/*4409*<br>*Maternal retrospective report, validated by comparison of prevalence to the prevalence in the general population in Europe*	Classification: ICD<br>Any cancer/*25*	Cohort/*10,612*<br>*France, ages 0-43 years (women average: 15.2, men average: 15.5)*<br>Logistic regression	Adjustment for year of birth
Van Duijn, 1994 (52)	Drugs to maintain pregnancy in the year before or during pregnancy/*7*<br>*Maternal retrospective report, validated by comparison to associations in another case-control study where cases of a different type of cancer did not report more medication use than controls*	Classification system not stated<br>ANLL/*80*	Case-control/*320*<br>*The Netherlands, ages 0-14 years, study reported results for both population- and cancer controls*<br>Conditional logistic regression	Matching on sex and age. Adjustment for sex, year of birth, maternal socioeconomic position, and in pregnancy: alcohol use, smoking, occupational hydrocarbon exposure, other medication

Reference	Exposure	Outcome	Materials and methods	
First author, year	Medication/*exposed*<br>*Ascertainment*<br>Exposure window: during pregnancy, unless otherwise stated	Classification system, type of cancer/*cases*	Design/*sample size*<br>*Study characteristics*<br>Statistical analysis	Method to account for confounding in the main analysis
				use, ultrasound, x-rays, viral infections
Van Steensel-Moll, 1985 (53)	Drugs to maintain pregnancy/*44*<br>*Maternal retrospective report*	Classification: ICD<br>Leukemias/*519*	Case-control/*1026*<br>*The Netherlands, ages 0-15 years*<br>Logistic regression	Matching on date of birth, sex and municipality. Adjustment for age and sex
Wen, 2002 (27)	Oral contraceptives/*123*<br>Hormones (not specified)/*54*<br>Each in the year before pregnancy, through pregnancy and/or during breastfeeding<br>*Maternal retrospective report*	Classification system not stated<br>ALL/*1842*	Case-control/*3828*<br>*US, ages 0-14 years*<br>Conditional logistic regression	Matching on age, ethnicity, and telephone area. Adjustment for parental income, education, ethnicity, smoking and alcohol use
PSYCHOTROPHICS				
Birch, 1990 (92)	Antiepileptics/*not reported*<br>Sedatives or anxiolytics/*not reported*<br>*Maternal retrospective report, validated in prescription records*	Classification: ICD<br>CNS tumors/*78*	Case-control/*234*<br>*UK, IRESCC, ages 0-15 years*<br>Mantel-Haenszel	Matching on age and sex
Buckley, 1989 (33)	Antiepileptics/*0*, Sedatives or anxiolytics/*5*<br>Each of the above in the year before pregnancy or during pregnancy<br>*Maternal retrospective report*	Classification system not stated<br>Hepatoblastoma/*75*	Case-control/*150*<br>*US and Canada, CCSG, upper age limit not stated, but the majority of children were under 2 years of age*<br>Conditional logistic regression	Matching on age
Cardy, 2006 (35)	Antiepileptics/*50*, Barbiturates/*30*<br>*Maternal retrospective report*	Classification: ICD-O<br>Main outcome:<br>Any brain tumor/*1218*<br>Secondary outcomes:<br>Astroglial/*623*, PNET/*259*, other glial/*327* by morphological codes	Case-control/*3441*<br>*Europe, North America, Australia, SEARCH, upper age limit 19 years*<br>Unconditional logistic regression	Matching, not further specified. Adjustment for center, age, sex, year of birth, and parental education. Vitamin supplementation (unclear whether in mothers or children) was considered as both a confounder and effect modifier

Reference	Exposure	Outcome	Materials and methods	
First author, year	Medication/*exposed*<br>*Ascertainment*<br>Exposure window: during pregnancy, unless otherwise stated	Classification system, type of cancer/*cases*	Design/*sample size*<br>*Study characteristics*<br>Statistical analysis	Method to account for confounding in the main analysis
Carozza, 1995 (3)	Amitriptyline/*1*, Amitriptyline HCL/*2*, Chlordiazepoxide HCL/*8*<br>Imipramine HCL/*1*, Nortriptyline HCL/*1*, Thiothixene/*1*<br>*Maternal retrospective report*	Classification: Histological Typing of Tumors of the Central Nervous System. International Histological Classification of Tumors<br>Any brain tumors/*361*	Case-control/*1444*<br>*US, SEER, upper age limit 18 years*<br>Conditional logistic regression, for main exposure (any n-nitrosatable drugs), descriptive statistics for medications stated here	Matching on age, sex and maternal ethnicity
Cordier, 1994 (36)	Barbiturates/*2*<br>*Maternal retrospective report*	Classification system not stated<br>Brain tumors/*75*	Case-control/*188*<br>*France, ages 0-15 years*<br>Conditional logistic regression	Matching on birth year. Adjustment for age, sex and maternal age
Gilman, 1989 (6)	Antidepressants/*93*, Antiepileptics/*37*, Hypnotics/*323*, Sedatives or anxiolytics/*1269*<br>*Maternal retrospective report and medical records. Children are considered exposed if medication use is present in either source*	Classification system not stated<br>Any cancer/*8059*	Case-control/*16,118*<br>*UK, OSCC, age not specified, only that cases are childhood deaths from cancer*<br>Conditional logistic regression	Matching on date of birth, sex and district. Adjustment for abdominal x-rays in pregnancy, birth order among live born siblings, maternal age, and socioeconomic position
Gold, 1978 (108)	Barbiturates/*6*<br>*Maternal retrospective report, uses cancer controls in addition to population controls, where the cancer controls are expected to be influenced by the same recall factors as cases*	Classification system not stated<br>Brain tumors/*73*	Case-control/*136*<br>*US, two control groups: healthy and cancer controls, we report healthy controls, ages 0-19 years*<br>Matched pairs OR, binomial test, exact 95% CI calculated based on the binomial distribution	Matching on ethnicity, sex, date and age of diagnosis
Goldhaber, 1990 (109)	Barbiturates/*58*<br>*Routinely collected health data*	Classification system not stated<br>Intracranial and spinal cord tumors/*86*	Case/control/*258*<br>*US, ages 0-19 years*<br>Conditional logistic regression	Matching on year of birth, sex, date of entry into the medical care program, birth at medical care program hospital
Grufferman, 1982 (7)	Anxiolytics 1 year prior to or during pregnancy/*7*<br>*Maternal retrospective report*	Classification system not stated<br>Rhabdomyosarcoma/*33*	Case-control/*132*<br>*US, ages 0-14 years*	Matching on age, sex, and ethnicity

Reference	Exposure	Outcome	Materials and methods	
First author, year	Medication/*exposed*<br>*Ascertainment*<br>Exposure window: during pregnancy, unless otherwise stated	Classification system, type of cancer/*cases*	Design/*sample size*<br>*Study characteristics*<br>Statistical analysis	Method to account for confounding in the main analysis
			"Unmatched methods", not specified further	
Gurney, 1997 (110)	Antiepileptics 1 month before pregnancy, during pregnancy or during breastfeeding/*27*<br>*Maternal retrospective report*	Classification: ICD-O<br>Any brain tumors/*540*	Case-control/*1341*<br>*US, ages 0-19 years*<br>Unconditional logistic regression	Matching on geographic area, age, sex, and study site
Hartley, 1988 (8)	Sedatives or anxiolytics/*not reported*<br>*Maternal retrospective report, validated in prescription data*	Classification system not stated<br>Bone or soft tissue sarcomas/*73*	Case-control/*219*<br>*UK, IRESCC, ages 0-15 years*<br>Mantel-Haenszel, 95% CI by Cornfield's formula	Matching. Matching factors not reported, but they refer to a paper where methods are described (age and sex)
Howe, 1989 (111)	Barbiturates or phenytoin/*5*<br>*Maternal retrospective report*	Classification system not stated<br>Brain tumors/*74*	Case-control/*212*<br>*Canada, ages 0-19 years*<br>Conditional logistic regression	Matching on sex, year of birth, and area of residence
Kinnier-Wilson, 1981 (100)	Sedatives/*849*<br>*Maternal retrospective report, asks antenatal clinics to verify exposure*	Classification system not stated<br>Any cancers/*2287*	Case-control/*4317*<br>*UK, age not specified, only that cases are childhood deaths from cancer, results are stratified by x-ray exposure*<br>Mantel-Haenszel	Matching, unclear on which factors, stratified on prenatal x-ray exposure
Kramer, 1987 (11)	Anxiolytics/*not reported*<br>*Maternal retrospective report, validated in medical records for a subsample. Validity compared between cases and controls*	Classification system not stated<br>Neuroblastoma/*104*	Case-control/*205*<br>*US, median age 1 year*<br>90% CI calculated by Miettinen's technique	Matching on telephone area code, ethnicity, and year of birth
Kuijten, 1990 (12)	Neurally active medications/*not reported*<br>*Maternal retrospective report*	Classification system not stated<br>Astrocytoma/*163*	Case-control/*326*<br>*US, ages 0-14 years*<br>Conditional logistic regression	Matching on telephone area code, ethnicity, and age
McCredie, 1994 (13)	Hypnotics, sedatives or anxiolytics in the month before or during pregnancy/*12*<br>*Maternal retrospective report*	Classification: ICD<br>Brain or cranial nerves tumors/*82*	Case-control/*246*<br>*Australia, ages 0-14 years*<br>Conditional logistic regression	Matching on sex and age. Adjustment for parental education
McKinney, 1999 (15)	Hypnotics or anxiolytics/*34*<br>*Routinely collected health data*	Classification: ICCC<br>Main outcome: Leukemia/*144*	Case-control/*415*<br>*Scotland, ages 0-14 years*<br>Conditional logistic regression	Matching on age, area of residence, and sex

Reference	Exposure	Outcome	Materials and methods	
First author, year	Medication/*exposed*<br>*Ascertainment*<br>Exposure window: during pregnancy, unless otherwise stated	Classification system, type of cancer/*cases*	Design/*sample size*<br>*Study characteristics*<br>Statistical analysis	Method to account for confounding in the main analysis
		Secondary outcome: ALL/*124*, lymphomas/*45*, CNS tumors/*75*, other solid tumors/*126*		
Momen, 2018 (112)	Antidepressants from 2 years prior until 1 month prior to pregnancy/*30,607*, Antidepressants from 1 month prior to pregnancy until end of pregnancy/*21,488*<br>Comparators are women who used antidepressants prior to, but not during pregnancy. Also analysis by duration<br>*Routinely collected health data, validated in a sensitivity analysis restricted to women with two or more prescription fills*	Classification: ICD<br>Main outcome: Any cancer/*1298*<br>Secondary outcomes: Leukemias/*524*, CNS/SNS tumors/*236*	Cohort/*915,128*<br>*Denmark, ages 0-14 years*<br>Cox regression	Adjustment for maternal age, parity, history of psychiatric disorders, inpatient psychiatric treatment in the two years before pregnancy, psychotropic comedication during pregnancy, education, calendar year at birth of child, and parental history of cancer
Ross, 2003 (19)	Hydroxyzine/*13*<br>*Routinely collected health data*	Classification system not stated<br>IL/*243*, ALL/*157*, AML/*77*	Case-control/*636*<br>*US, ages 0-18 months*<br>Conditional logistic regression	Matching on birth year and telephone area. Adjustment for maternal age, education and income
Robison, 1989 (37)	Sedatives in the year before pregnancy, through pregnancy and/or during breastfeeding/*17*<br>Also analyses by duration and frequency<br>*Maternal retrospective report, validated by comparisons to associations in four other independent case-control studies where cases of different cancers did not report more medication use than controls*	Classification system not stated<br>ANLL/*204*	Case-control/*408*<br>*US, ages 0-18 years*<br>Conditional logistic regression	Matching on date of birth, ethnicity, and telephone area code, stated that they adjust for confounders, but these are not specified
Rodvall, 1990 (47)	Sedatives or sleeping pills (unspecified)/*28*<br>*Routinely collected health data*	Classification system not stated<br>Any cancer/*95*	Case-control/*285*<br>*Sweden, ages 0-16 years*<br>Multiple conditional logistic regression	Matching on sex and birth year and month. Adjustment for maternal age, drug use, obstetric complications, previous miscarriage, socioeconomic position and child gestational age.

Reference	Exposure	Outcome	Materials and methods	
First author, year	Medication/*exposed*<br>*Ascertainment*<br>Exposure window: during pregnancy, unless otherwise stated	Classification system, type of cancer/*cases*	Design/*sample size*<br>*Study characteristics*<br>Statistical analysis	Method to account for confounding in the main analysis
Roman, 1997 (48)	Anticonvulsants/*8*<br>*Routinely collected health data*	Classification system not stated<br>Main outcomes: Leukemias/*143*, NHL/*34*<br>Secondary outcomes: ALL/*113*, AML/*15*	Case-control/*429*<br>*England, ages 3 months to 29 years*<br>Conditional logistic regression	Matching on hospital catchment area of birth, sex, year and month of birth
Salonen, 1976 (20)	Neuroleptics/*7*, Antineurotics/*11*, Barbiturates/*29*<br>*Routinely collected health data*	Classification: ICD<br>Main outcomes: Leukemias/*373*, brain tumors/*245*, other tumors/*354*<br>Secondary outcomes: Subgroups of the "other tumors"-category; kidney tumors/*96*, eye tumors/*37*, bone tumors/*56* (only reported for most commonly used medications)	Case-control/*1944*<br>*Finland, ages 0-14 years*<br>MacNemar's test, descriptive for rarely used medications	Matching on date of birth
Sanders and Draper, 1979 (49)	Phenytoin/*18*, Phenobarbitone/*32*<br>*Maternal retrospective report and medical records, findings from the two data sources are reported separately*	Classification system not stated<br>Any cancer/*39*	Case-controls/*61*<br>*UK, Oxford Survey of Childhood Cancers, ages 0-15. All children were born to mothers with epilepsy*<br>Comparison of the frequencies	It would appear that matching was done, but it is unclear
Sharpe & Franco, 1996 (22)	Anticonvulsants/*5*<br>*Maternal retrospective report*	Classification system not stated<br>Nephroblastoma/*109*	Case-control/*327*<br>*Brazil, age range not reported, mean age 3 years and 5 months*<br>Logistic regression	Matching on age, sex, and interviewer, stratified on socioeconomic position
Shaw, 2004 (23)	CNS depressants (tranquilizers and sleeping pills)/*14*<br>Epileptic medication/*7*<br>*Maternal retrospective report*	Classification: ICD<br>ALL/*789*	Case-control/*1578*<br>*Canada, age 0-14 years*<br>Conditional logistic regression	Matching on sex and age at diagnosis. Adjustment for maternal age and education
Stålberg, 2010 (25)	Neuroleptics/*7*, Antiepileptics/*<10*, Antipsychotics/*<10*, Anxiolytics/*<10*	Classification: ICD<br>Brain tumor (ICD-7 code 193)/*512*	Case-control/*1037*<br>*Sweden, ages 0-15 years*<br>Logistic regression	Matching on sex and birth year. Adjustment for maternal age, parity, country of birth,

Reference	Exposure	Outcome	Materials and methods	
First author, year	Medication/*exposed*<br>*Ascertainment*<br>Exposure window: during pregnancy, unless otherwise stated	Classification system, type of cancer/*cases*	Design/*sample size*<br>*Study characteristics*<br>Statistical analysis	Method to account for confounding in the main analysis
	Routinely collected health data			and level of hospital (primary, secondary, or tertiary)
Van Steensel-Moll, 1985 (53)	Sedatives or sleeping pills/*21*<br>*Maternal retrospective report*	Classification: ICD<br>Leukemias/*519*	Case-control/*1026*<br>*The Netherlands, ages 0-15 years*<br>Logistic regression	Matching on date of birth, sex and municipality. Adjustment for age and sex
Wen, 2002 (27)	Sedatives, tranquilizers, or sleeping pills/*17*, Dilantin or other antiepileptic (seizure) drugs/*7*<br>Each of the above in the year before pregnancy, through pregnancy and/or during breastfeeding<br>*Maternal retrospective report*	Classification system not stated<br>ALL/*1842*	Case-control/*3828*<br>*US, ages 0-14 years*<br>Conditional logistic regression	Matching on age, ethnicity, and telephone area. Adjustment for parental income, education, ethnicity, smoking and alcohol use
SKIN TREATMENT				
Bonaventure, 2015 (1)	Dermatologicals (ATC group D)/*472*<br>*Routinely collected health data*	Classification: ICD<br>ALL/*725*, AML/*128*, NHL/*83*, HL/*31*, astrocytoma/*100*, medulloblastoma/*48*, rhabdomyosarcoma/*54*, Ewings sarcoma/*20*, neuroblastoma/*78*, nephroblasoma/*60*	Case-control/*4122*<br>*UK, UKCCS, mean age 5.8 (max age 15 years)*<br>Unconditional logistic regression	Matching on sex, month and year of birth, and region of residence. Adjustment for year of birth
Gilman, 1989 (6)	Local skin treatment/*22*<br>*Maternal retrospective report and medical records. Children are considered exposed if medication use is present in either source*	Classification system not stated<br>Any cancer/*8059*	Case-control/*16,118*<br>*UK, OSCC, age not specified, only that cases are childhood deaths from cancer*<br>Conditional logistic regression	Matching on date of birth, sex and district. Adjustment for abdominal x-rays in pregnancy, birth order among live born siblings, maternal age, and socioeconomic position
Ross, 1996 (57)	Acne medications/*7*<br>*Maternal retrospective report*	Classification system not stated<br>IL/*84*	Case-control/*181*<br>*US, ages 0-1 year*<br>Conditional logistic regression	Matching on year of birth, geography and ethnicity. Adjustment for maternal education
THYROID THERAPY				

Reference	Exposure	Outcome	Materials and methods	
First author, year	Medication/*exposed*<br>*Ascertainment*<br>Exposure window: during pregnancy, unless otherwise stated	Classification system, type of cancer/*cases*	Design/*sample size*<br>*Study characteristics*<br>Statistical analysis	Method to account for confounding in the main analysis
Cook, 2004 (4)	Levothyroxine during pregnancy or breastfeeding/*17*<br>*Maternal retrospective report*	Classification system not stated<br>Neuroblastoma/*504*	Case-control/*1008*<br>*US and Canada, Children's Cancer Group, upper age limit 18 years*<br>Conditional logistic regression	Matching on age. Adjustment for maternal age, education, and ethnicity
Gilman, 1989 (6)	Thyroid hormone/*20*<br>*Maternal retrospective report and medical records. Children are considered exposed if medication use is present in either source*	Classification system not stated<br>Any cancer/*8059*	Case-control/*16,118*<br>*UK, OSCC, age not specified, only that cases are childhood deaths from cancer*<br>Conditional logistic regression	Matching on date of birth, sex and district. Adjustment for abdominal x-rays in pregnancy, birth order among live born siblings, maternal age, and socioeconomic position
Ognjanovic, 2009 (30)	Thyroid medication/*15*<br>*Maternal retrospective report*	Classification system not stated<br>Main outcome: Leukemias/*158*<br>Secondary outcomes: ALL/*97*, AML/*61*	Case-control/*331*<br>*US, Children's Oncology Group, ages 0-19 years, all cases and controls have Down syndrome*<br>Descriptive statistics	Matching on age and sex
Ross, 2003 (19)	Levothyroxine/*8*<br>*Routinely collected health data*	Classification system not stated<br>IL/*243*, ALL/*157*, AML/*77*	Case-control/*636*<br>*US, ages 0-18 months*<br>Conditional logistic regression	Matching on birth year and telephone area. Adjustment for maternal age, education and income
Shaw, 2004 (23)	Hormones (fertility and thyroid hormones)/*31*<br>*Maternal retrospective report*	Classification: ICD<br>ALL/*789*	Case-control/*1578*<br>*Canada, age 0-14 years*<br>Conditional logistic regression	Matching on sex and age at diagnosis. Adjustment for maternal age and education
MISCELLANEOUS MEDICATIONS				
Bonaventure, 2015 (1)	Blood and blood forming organs (ATC group B)/*1155*<br>*Routinely collected health data*	Classification: ICD<br>ALL/*725*, AML/*128*, NHL/*83*, HL/*31*, astrocytoma/*100*, medulloblastoma/*48*, rhabdomyosarcoma/*54*, Ewings	Case-control/*4122*<br>*UK, UKCCS, mean age 5.8 (max age 15 years)*<br>Unconditional logistic regression	Matching on sex, month and year of birth, and region of residence. Adjustment for year of birth

Reference<br>First author, year	Exposure<br>Medication/*exposed*<br>*Ascertainment*<br>Exposure window: during pregnancy, unless otherwise stated	Outcome<br>Classification system, type of cancer/*cases*	Materials and methods<br>Design/*sample size*<br>*Study characteristics*<br>Statistical analysis	Method to account for confounding in the main analysis
		sarcoma/*20*, neuroblastoma/*78*, nephroblasoma/*60*		
Gilman, 1989 (6)	Gastro-intestinal sedatives/*43*, Anticoagulants/*6*, Vasodilators/*8*,<br>Other cardiac/*29*, Pituitary hormone/*186*, Local ear, nose, throat treatment/*39*, Local eye treatment/*9*<br>*Maternal retrospective report and medical records. Children are considered exposed if medication use is present in either source*	Classification system not stated<br>Any cancer/*8059*	Case-control/*16,118*<br>*UK, OSCC, age not specified, only that cases are childhood deaths from cancer*<br>Conditional logistic regression	Matching on date of birth, sex and district. Adjustment for abdominal x-rays in pregnancy, birth order among live born siblings, maternal age, and socioeconomic position
McKinney, 1987 (14)	Topical steroids/*not reported*<br>*Maternal retrospective report and medical records, findings from the two data sources are reported separately*	Classification system not stated<br>Main outcome: Leukemias/lymphomas/*234*<br>Secondary outcomes: Leukemias/*171*<br>Only reported separately for significant associations	Case-control/*702*<br>*UK, IRESCC, ages 0-15 years*<br>Mantel-Haenszel	Matching on age and sex
Michalek, 1996 (29)	Antipyretics/*20*<br>*Maternal retrospective report*	Classification system not stated<br>Neuroblastoma/*183*	Case-control/*555*<br>*US, ages 0-14 years*<br>Unconditional logistic regression	Matching on year of birth
Salonen, 1976 (20)	Papaverine/*13*<br>*Routinely collected health data*	Classification: ICD<br>Main outcomes: Leukemias/*373*, brain tumors/*245*, other tumors/*354*<br>Secondary outcomes: Subgroups of the “other tumors”-category; kidney tumors/*96*, eye tumors/*37*, bone tumors/*56* (only reported for most commonly used medications)	Case-control/*1944*<br>*Finland, ages 0-14 years*<br>MacNemar’s test, descriptive for rarely used medications	Matching on date of birth

[a]Studies on IVF or unspecified assisted reproduction have been included, as assisted reproduction will almost always include use of medications.
ALL: Acute lymphocytic leukemias, AML: Acute myeloid leukemias, ANLL: Acute non-lymphocytic leukemias, ATC: Anatomical Therapeutic Chemical Classification System, CI: Confidence Interval, CNS: Central Nervous System, GnRH: Gonadotropin-releasing hormone, hCG: human chorionic gonadotropin, HIV: Human immunodeficiency virus, HL:

Hodgkin’s lymphoma, hMG: human menopausal gonadotropin, ICCC: International Classification of Childhood Cancers, ICD: International Classification of Diseases, ICD-O: International Classification of Diseases for Oncology, ICSI: intracytoplasmic sperm injection, IL: Infant leukemias, IVF: in vitro fertilization, MLL: mixed-lineage leukemia, NHL: non-Hodgkin’s lymphoma, NSAID: Non-steroidal anti-inflammatory drug, PNET: Primitive Neuroectodermal Tumor, SEP: Socio-economic position, SNS: Sympathetic Nervous System.

Web Figure 1. Example of Study Design Diagram

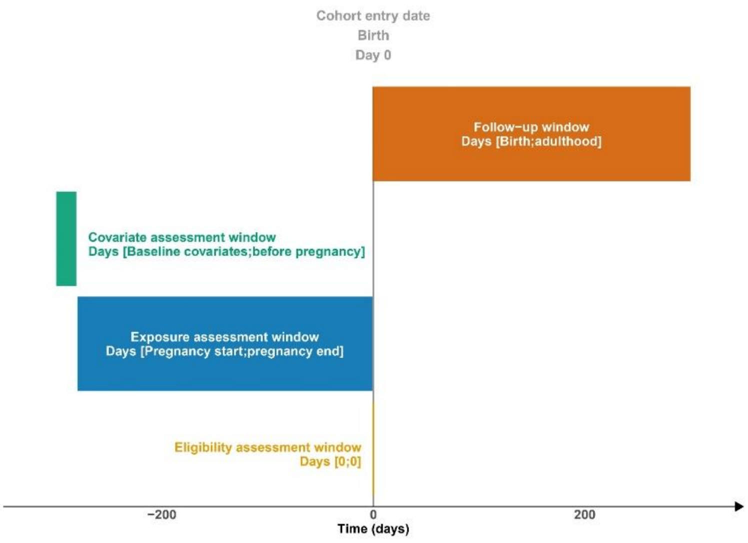

Web Figure 1: Example of study design diagram for diethylstilbestrol and vaginal adenocarcinoma (113). The box illustrating follow-up has been truncated to increase readability.

Web Figure 2. Example of Directed Acyclic Graph

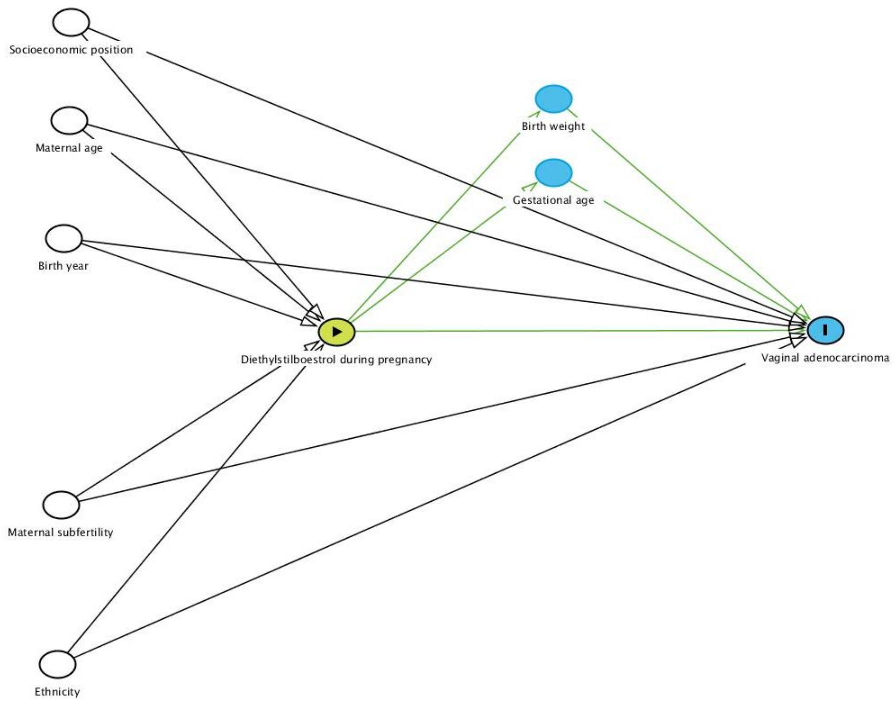

Web Figure 2: Example of directed acyclic graph for diethylstilbestrol and vaginal adenocarcinoma (114). Arrows between covariates are not displayed for reasons of readability. The white circles represent potential confounders, the yellow circle represent exposure, the blue circle with an "I" represents the outcome, the other blue circles represent intermediate variables. The green arrows indicate the causal pathways.

References

1. Bonaventure A, Simpson J, Ansell P, et al. Prescription drug use during pregnancy and risk of childhood cancer – Is there an association? *Cancer Epidemiology*. 2015;39(1):73–78.
2. Bunin GR, Buckley JD, Boesel CP, et al. Risk factors for astrocytic glioma and primitive neuroectodermal tumor of the brain in young children: a report from the Children's Cancer Group. *Cancer Epidemiol. Biomarkers Prev.* 1994;3(3):197–204.
3. Carozza SE, Olshan AF, Faustman EM, et al. Maternal exposure to N-nitrosatable drugs as a risk factor for childhood brain tumours. *Int J Epidemiol*. 1995;24(2):308–312.
4. Cook MN, Olshan AF, Guess HA, et al. Maternal medication use and neuroblastoma in offspring. *Am. J. Epidemiol.* 2004;159(8):721–731.
5. Couto AC, Ferreira JD, Pombo-de-Oliveira MS, et al. Pregnancy, maternal exposure to analgesic medicines, and leukemia in Brazilian children below 2 years of age: *European Journal of Cancer Prevention*. 2015;24(3):245–252.
6. Gilman EA, Wilson LM, Kneale GW, et al. Childhood cancers and their association with pregnancy drugs and illnesses. *Paediatr Perinat Epidemiol*. 1989;3(1):66–94.
7. Grufferman S, Wang HH, DeLong ER, et al. Environmental factors in the etiology of rhabdomyosarcoma in childhood. *J. Natl. Cancer Inst.* 1982;68(1):107–113.
8. Hartley AL, Birch JM, McKinney PA, et al. The Inter-Regional Epidemiological Study of Childhood Cancer (IRESCC): case control study of children with bone and soft tissue sarcomas. *Br. J. Cancer*. 1988;58(6):838–842.
9. Heck JE, Omidakhsh N, Azary S, et al. A case–control study of sporadic retinoblastoma in relation to maternal health conditions and reproductive factors: a report from the Children's Oncology group. *BMC Cancer*. 2015;15(1):735.
10. Kelty E, Hulse G. A Retrospective Cohort Study of Birth Outcomes in Neonates Exposed to Naltrexone in Utero: A Comparison with Methadone-, Buprenorphine- and Non-opioid-Exposed Neonates. *Drugs*. 2017;77(11):1211–1219.
11. Kramer S, Ward E, Meadows AT, et al. Medical and drug risk factors associated with neuroblastoma: a case-control study. *J. Natl. Cancer Inst.* 1987;78(5):797–804.
12. Kuijten RR, Bunin GR, Nass CC, et al. Gestational and familial risk factors for childhood astrocytoma: results of a case-control study. *Cancer Res.* 1990;50(9):2608–2612.
13. McCredie M, Maisonneuve P, Boyle P. Antenatal risk factors for malignant brain tumours in new south wales children. *Int. J. Cancer*. 1994;56(1):6–10.
14. McKinney PA, Cartwright RA, Saiu JM, et al. The inter-regional epidemiological study of childhood cancer (IRESCC): a case control study of aetiological factors in leukaemia and lymphoma. *Arch. Dis. Child.* 1987;62(3):279–287.
15. McKinney PA, Juszczak E, Findlay E, et al. Pre- and perinatal risk factors for childhood leukaemia and other malignancies: a Scottish case control study. *Br. J. Cancer*. 1999;80(11):1844–1851.
16. Ognjanovic S, Blair C, Spector LG, et al. Analgesic use during pregnancy and risk of infant leukaemia: A Children's Oncology Group study. *Br J Cancer*. 2011;104(3):532–536.
17. Pombo-de-Oliveira MS, Koifman S, Brazilian Collaborative Study Group of Infant Acute Leukemia. Infant Acute Leukemia and Maternal Exposures during Pregnancy. *Cancer Epidemiology Biomarkers & Prevention*. 2006;15(12):2336–2341.
18. Preston-Martin S, Yu MC, Benton B, et al. N-Nitroso compounds and childhood brain tumors: a case-control study. *Cancer Res.* 1982;42(12):5240–5245.
19. Ross JA, Xie Y, Davies SM, et al. Prescription medication use during pregnancy and risk of infant leukemia (United States). *Cancer Causes Control*. 2003;14(5):447–451.
20. Salonen T. Prenatal and perinatal factors in childhood cancer. *Ann. Clin. Res.* 1976;8(1):27–42.
21. Schüz J, Weihkopf T, Kaatsch P. Medication use during pregnancy and the risk of childhood cancer in the offspring. *Eur J Pediatr*. 2007;166(5):433–441.
22. Sharpe CR, Franco EL. Use of dipyrone during pregnancy and risk of Wilms' tumor. Brazilian Wilms' Tumor Study Group. *Epidemiology*. 1996;7(5):533–535.
23. Shaw AK, Infante-Rivard C, Morrison HI. Use of medication during pregnancy and risk of childhood leukemia (Canada). *Cancer Causes Control*. 2004;15(9):931–937.
24. Shu XO, Nesbit ME, Buckley JD, et al. An exploratory analysis of risk factors for childhood malignant germ-cell tumors: report from the Childrens Cancer Group (Canada, United States). *Cancer Causes Control*. 1995;6(3):187–198.
25. Stålberg K, Haglund B, Strömberg B, et al. Prenatal exposure to medicines and the risk of childhood brain tumor. *Cancer Epidemiology*. 2010;34(4):400–404.

26. Wang Y, Gao P, Liang G, et al. Maternal prenatal exposure to environmental factors and risk of childhood acute lymphocytic leukemia: A hospital-based case-control study in China. *Cancer Epidemiology*. 2019;58:146–152.
27. Wen W, Shu XO, Potter JD, et al. Parental medication use and risk of childhood acute lymphoblastic leukemia. *Cancer*. 2002;95(8):1786–1794.
28. Zanrosso CW, Emerenciano M, Goncalves BA d. A, et al. N -Acetyltransferase 2 Polymorphisms and Susceptibility to Infant Leukemia with Maternal Exposure to Dipyrone during Pregnancy. *Cancer Epidemiology Biomarkers & Prevention*. 2010;19(12):3037–3043.
29. Michalek AM, Buck GM, Nasca PC, et al. Gravid health status, medication use, and risk of neuroblastoma. *Am. J. Epidemiol.* 1996;143(10):996–1001.
30. Ognjanovic S, Puumala S, Spector LG, et al. Maternal health conditions during pregnancy and acute leukemia in children with Down syndrome: A Children's Oncology Group study. *Pediatr. Blood Cancer*. 2009;52(5):602–608.
31. Seppälä LK, Vettenranta K, Pitkäniemi J, et al. Maternal diabetes and risk of childhood cancer in the offspring. *Int. J. Cancer*. 2020;147(3):662–668.
32. Søegaard SH, Rostgaard K, Kamper-Jørgensen M, et al. Maternal diabetes and risk of childhood acute lymphoblastic leukaemia in the offspring. *Br J Cancer*. 2018;118(1):117–120.
33. Buckley JD, Sather H, Ruccione K, et al. A case-control study of risk factors for hepatoblastoma. A report from the Childrens Cancer Study Group. *Cancer*. 1989;64(5):1169–1176.
34. Bunin GR, Meadows AT, Emanuel BS, et al. Pre- and postconception factors associated with sporadic heritable and nonheritable retinoblastoma. *Cancer Res.* 1989;49(20):5730–5735.
35. Cardy AH, Little J, McKean-Cowdin R, et al. Maternal medication use and the risk of brain tumors in the offspring: The SEARCH international case-control study. *Int. J. Cancer*. 2006;118(5):1302–1308.
36. Cordier S, Iglesias M-J, Le Goaster C, et al. Incidence and risk factors for childhood brain tumors in the ILE DE France. *Int. J. Cancer*. 1994;59(6):776–782.
37. Robison LL, Buckley JD, Daigle AE, et al. Maternal drug use and risk of childhood nonlymphoblastic leukemia among offspring. An epidemiologic investigation implicating marijuana (a report from the Childrens Cancer Study Group). *Cancer*. 1989;63(10):1904–1911.
38. Bunin GR, Kramer S, Marrero O, et al. Gestational risk factors for Wilms' tumor: results of a case-control study. *Cancer Res.* 1987;47(11):2972–2977.
39. Olshan AF, Breslow NE, Falletta JM, et al. Risk factors for Wilms tumor. Report from the National Wilms Tumor Study. *Cancer*. 1993;72(3):938–944.
40. Gradel KO, Kaerlev L. Antibiotic use from conception to diagnosis of child leukaemia as compared to the background population: A nested case-control study. *Pediatr Blood Cancer*. 2015;62(7):1155–1161.
41. Infante-Rivard C, Fortier I, Olson E. Markers of infection, breast-feeding and childhood acute lymphoblastic leukaemia. *Br. J. Cancer*. 2000;83(11):1559–1564.
42. Kaatsch P, Scheidemann-Wesp U, Schüz J. Maternal use of antibiotics and cancer in the offspring: results of a case–control study in Germany. *Cancer Causes Control*. 2010;21(8):1335–1345.
43. Kumar A, Vashist M, Rathee R. Maternal Factors and Risk of Childhood Leukemia. *Asian Pacific Journal of Cancer Prevention*. 2014;15(2):781–784.
44. Kwan ML, Metayer C, Crouse V, et al. Maternal illness and drug/medication use during the period surrounding pregnancy and risk of childhood leukemia among offspring. *Am. J. Epidemiol.* 2007;165(1):27–35.
45. Momen NC, Olsen J, Gissler M, et al. Exposure to systemic antibacterial medications during pregnancy and risk of childhood cancer. *Pharmacoepidemiol Drug Saf*. 2015;24(8):821–829.
46. Naumburg E, Bellocco R, Cnattingius S, et al. Perinatal exposure to infection and risk of childhood leukemia. *Med. Pediatr. Oncol.* 2002;38(6):391–397.
47. Rodvall Y, Pershagen G, Hrubec Z, et al. Prenatal X-ray exposure and childhood cancer in Swedish twins. *Int. J. Cancer*. 1990;46(3):362–365.
48. Roman E, Ansell P, Bull D. Leukaemia and non-Hodgkin's lymphoma in children and young adults: are prenatal and neonatal factors important determinants of disease? *Br. J. Cancer*. 1997;76(3):406–415.
49. Sanders BM, Draper GJ. Childhood cancer and drugs in pregnancy. *Br Med J*. 1979;1(6165):717–718.
50. Shaw AK, Li P, Infante-Rivard C. Early infection and risk of childhood brain tumors (Canada). *Cancer Causes Control*. 2006;17(10):1267–1274.

51. Thapa PB, Whitlock JA, Brockman Worrell KG, et al. Prenatal exposure to metronidazole and risk of childhood cancer: a retrospective cohort study of children younger than 5 years. *Cancer*. 1998;83(7):1461–1468.
52. van Duijn CM, van Steensel-Moll HA, Coebergh JW, et al. Risk factors for childhood acute non-lymphocytic leukemia: an association with maternal alcohol consumption during pregnancy? *Cancer Epidemiol. Biomarkers Prev.* 1994;3(6):457–460.
53. van Steensel-Moll HA, Valkenburg HA, Vandenbroucke JP, et al. Are maternal fertility problems related to childhood leukaemia? *Int J Epidemiol*. 1985;14(4):555–559.
54. Walsh LK, Donelle J, Dodds L, et al. Health outcomes of young children born to mothers who received 2009 pandemic H1N1 influenza vaccination during pregnancy: retrospective cohort study. *BMJ*. 2019;l4151.
55. Ye X, Monchka BA, Righolt CH, et al. Maternal use of antibiotics and cancer incidence risk in offspring: A population-based cohort study in Manitoba, Canada. *Cancer Med*. 2019;8(11):5367–5372.
56. McKinney PA, Cartwright RA, Stiller CA, et al. Inter-Regional Epidemiological Study of Childhood Cancer (IRESCC): childhood cancer and the consumption of debendox and related drugs in pregnancy. *Br. J. Cancer*. 1985;52(6):923–929.
57. Ross JA, Potter JD, Reaman GH, et al. Maternal exposure to potential inhibitors of DNA topoisomerase II and infant leukemia (United States): A report from the Children's Cancer Group. *Cancer Causes Control*. 1996;7(6):581–590.
58. Tegethoff M, Olsen J, Schaffner E, et al. Asthma During Pregnancy and Clinical Outcomes in Offspring: A National Cohort Study. *PEDIATRICS*. 2013;132(3):483–491.
59. Ajrouche R, Rudant J, Orsi L, et al. Maternal reproductive history, fertility treatments and folic acid supplementation in the risk of childhood acute leukemia: the ESTELLE Study. *Cancer Causes Control*. 2014;25(10):1283–1293.
60. Bradbury BD, Jick H. In vitro fertilization and childhood retinoblastoma. *Br J Clin Pharmacol*. 2004;58(2):209–211.
61. Brinton LA, Krüger Kjær S, Thomsen BL, et al. Childhood tumor risk after treatment with ovulation-stimulating drugs. *Fertility and Sterility*. 2004;81(4):1083–1091.
62. Foix-L'Helias L, Aerts I, Marchand L, et al. Are children born after infertility treatment at increased risk of retinoblastoma? *Human Reproduction*. 2012;27(7):2186–2192.
63. Hargreave M, Jensen A, Nielsen TSS, et al. Maternal use of fertility drugs and risk of cancer in children-A nationwide population-based cohort study in Denmark: Maternal use of fertility drugs and risk of cancer in children. *Int. J. Cancer*. 2015;136(8):1931–1939.
64. Hargreave M, Jensen A, Hansen MK, et al. Association Between Fertility Treatment and Cancer Risk in Children. *JAMA*. 2019;322(22):2203–2210.
65. Heck JE, Lombardi CA, Meyers TJ, et al. Perinatal characteristics and retinoblastoma. *Cancer Causes Control*. 2012;23(9):1567–1575.
66. Heck JE, Meyers TJ, Lombardi C, et al. Case-control study of birth characteristics and the risk of hepatoblastoma. *Cancer Epidemiology*. 2013;37(4):390–395.
67. Kallen B, Finnstrom O, Lindam A, et al. Cancer Risk in Children and Young Adults Conceived by In Vitro Fertilization. *PEDIATRICS*. 2010;126(2):270–276.
68. Klip H, Burger CW, de Kraker J, et al. Risk of cancer in the offspring of women who underwent ovarian stimulation for IVF. *Human Reproduction*. 2001;16(11):2451–2458.
69. Lerner-Geva L, Boyko V, Ehrlich S, et al. Possible risk for cancer among children born following assisted reproductive technology in Israel: Lerner-geva et al. *Pediatr Blood Cancer*. 2017;64(4):e26292.
70. Mallol-Mesnard N, Menegaux F, Lacour B, et al. Birth characteristics and childhood malignant central nervous sytem tumors: The ESCALE study (French Society for Childhood Cancer). *Cancer Detection and Prevention*. 2008;32(1):79–86.
71. McLaughlin CC, Baptiste MS, Schymura MJ, et al. Maternal and Infant Birth Characteristics and Hepatoblastoma. *American Journal of Epidemiology*. 2006;163(9):818–828.
72. Munzer C, Menegaux F, Lacour B, et al. Birth-related characteristics, congenital malformation, maternal reproductive history and neuroblastoma: The ESCALE study (SFCE). *Int. J. Cancer*. 2007;122(10):2315–2321.
73. Olshan AF, Smith J, Cook MN, et al. Hormone and fertility drug use and the risk of neuroblastoma: a report from the Children's Cancer Group and the Pediatric Oncology Group. *Am. J. Epidemiol.* 1999;150(9):930–938.
74. Petridou ET, Sergentanis TN, Panagopoulou P, et al. In vitro fertilization and risk of childhood leukemia in Greece and Sweden: IVF and Childhood ALL. *Pediatr. Blood Cancer*. 2012;58(6):930–936.
75. Puumala SE, Ross JA, Olshan AF, et al. Reproductive history, infertility treatment, and the risk of acute leukemia in children with down syndrome: A Report From the Children's Oncology Group. *Cancer*. 2007;110(9):2067–2074.

76. Puumala SE, Spector LG, Wall MM, et al. Infant leukemia and parental infertility or its treatment: a Children's Oncology Group report. *Human Reproduction*. 2010;25(6):1561–1568.
77. Puumala SE, Ross JA, Wall MM, et al. Pediatric germ cell tumors and parental infertility and infertility treatment: A Children's Oncology Group report. *Cancer Epidemiology*. 2011;35(5):e25–e31.
78. Puumala SE, Ross JA, Feusner JH, et al. Parental infertility, infertility treatment and hepatoblastoma: a report from the Children's Oncology Group. *Human Reproduction*. 2012;27(6):1649–1656.
79. Reigstad MM, Larsen IK, Myklebust TÅ, et al. Risk of Cancer in Children Conceived by Assisted Reproductive Technology. *Pediatrics*. 2016;137(3):e20152061.
80. Rudant J, Amigou A, Orsi L, et al. Fertility treatments, congenital malformations, fetal loss, and childhood acute leukemia: The ESCALE study (SFCE): Maternal and Birth Characteristics and AL. *Pediatr. Blood Cancer*. 2013;60(2):301–308.
81. Schüz J, Kaatsch P, Kaletsch U, et al. Association of childhood cancer with factors related to pregnancy and birth. *Int J Epidemiol*. 1999;28(4):631–639.
82. Schüz J, Kaletsch U, Meinert R, et al. Risk factors for neuroblastoma at different stages of disease. Results from a population-based case-control study in Germany. *J Clin Epidemiol*. 2001;54(7):702–709.
83. Spector LG, Brown MB, Wantman E, et al. Association of In Vitro Fertilization With Childhood Cancer in the United States. *JAMA Pediatr*. 2019;173(6):e190392.
84. Sundh KJ, Henningsen A-KA, Kallen K, et al. Cancer in children and young adults born after assisted reproductive technology: a Nordic cohort study from the Committee of Nordic ART and Safety (CoNARTaS). *Human Reproduction*. 2014;29(9):2050–2057.
85. Wainstock T, Walfisch A, Shoham-Vardi I, et al. Fertility treatments and pediatric neoplasms of the offspring: results of a population-based cohort with a median follow-up of 10 years. *American Journal of Obstetrics and Gynecology*. 2017;216(3):314.e1-314.e14.
86. Benhammou V, Warszawski J, Bellec S, et al. Incidence of cancer in children perinatally exposed to nucleoside reverse transcriptase inhibitors: *AIDS*. 2008;22(16):2165–2177.
87. Hleyhel M, Goujon S, Delteil C, et al. Risk of cancer in children exposed to didanosine in utero: *AIDS*. 2016;30(8):1245–1256.
88. Chambers CD, Johnson DL, Xu R, et al. Birth outcomes in women who have taken adalimumab in pregnancy: A prospective cohort study. *PLoS ONE*. 2019;14(10):e0223603.
89. Chaparro M, Verreth A, Lobaton T, et al. Long-Term Safety of In Utero Exposure to Anti-TNFα Drugs for the Treatment of Inflammatory Bowel Disease: Results from the Multicenter European TEDDY Study: *American Journal of Gastroenterology*. 2018;113(3):396–403.
90. Green DM, Fiorello A, Zevon MA, et al. Birth defects and childhood cancer in offspring of survivors of childhood cancer. *Arch Pediatr Adolesc Med*. 1997;151(4):379–383.
91. Li FP, Fine W, Jaffe N, et al. Offspring of patients treated for cancer in childhood. *J. Natl. Cancer Inst.* 1979;62(5):1193–1197.
92. Birch JM, Hartley AL, Teare MD, et al. The inter-regional epidemiological study of childhood cancer (IRESCC): Case-control study of children with central nervous system tumours. *British Journal of Neurosurgery*. 1990;4(1):17–25.
93. Erjaee A, Niknam M, Sadeghi A, et al. A Significant Breakthrough in the Incidence of Childhood Cancers and Evaluation of its Risk Factors in Southern Iran. *Indian J Med Paediatr Oncol*. 2017;38(2):158–164.
94. Gholami A, Salarilak S, Hejazi S, et al. Parental risk factors of childhood acute leukemia: a case-control study. *J Res Health Sci*. 2011;11(2):69–76.
95. Gold E, Gordis L, Tonascia J, et al. RISK FACTORS FOR BRAIN TUMORS IN CHILDREN. *American Journal of Epidemiology*. 1979;109(3):309–319.
96. Golding J, Paterson M, Kinlen LJ. Factors associated with childhood cancer in a national cohort study. *Br. J. Cancer*. 1990;62(2):304–308.
97. Hargreave M, Mørch LS, Andersen KK, et al. Maternal use of hormonal contraception and risk of childhood leukaemia: a nationwide, population-based cohort study. *Lancet Oncol*. 2018;19(10):1307–1314.
98. Herbst AL, Ulfelder H, Poskanzer DC. Adenocarcinoma of the vagina. Association of maternal stilbestrol therapy with tumor appearance in young women. *N. Engl. J. Med.* 1971;284(15):878–881.
99. Johnston HE, Mann JR, Williams J, et al. The Inter-Regional, Epidemiological Study of Childhood Cancer (IRESCC): case-control study in children with germ cell tumours. *Carcinogenesis*. 1986;7(5):717–722.
100. Kinnier-Wilson LM, Kneale GW, Stewart AM. Childhood cancer and pregnancy drugs. *Lancet*. 1981;2(8241):314–315.
101. Linet MS, Gridley G, Cnattingius S, et al. Maternal and perinatal risk factors for childhood brain tumors (Sweden). *Cancer Causes Control*. 1996;7(4):437–448.

102. Lupo PJ, Danysh HE, Skapek SX, et al. Maternal and birth characteristics and childhood rhabdomyosarcoma: a report from the Children's Oncology Group. *Cancer Causes Control*. 2014;25(7):905–913.
103. Operskalski EA, Preston-Martin S, Henderson BE, et al. A CASE-CONTROL STUDY OF OSTEOSARCOMA IN YOUNG PERSONS. *American Journal of Epidemiology*. 1987;126(1):118–126.
104. Ou SX, Han D, Severson RK, et al. Birth characteristics, maternal reproductive history, hormone use during pregnancy, and risk of childhood acute lymphocytic leukemia by immunophenotype (United States). *Cancer Causes Control*. 2002;13(1):15–25.
105. Shankar S, Davies S, Giller R, et al. In utero exposure to female hormones and germ cell tumors in children. *Cancer*. 2006;106(5):1169–1177.
106. Stewart A, Webb J, Hewitt D. A survey of childhood malignancies. *Br Med J*. 1958;1(5086):1495–1508.
107. Tournaire M, Epelboin S, Devouche E, et al. Adverse health effects in children of women exposed in utero to diethylstilbestrol (DES). *Therapie*. 2016;71(4):395–404.
108. Gold E, Gordis L, Tonascia J, et al. Increased risk of brain tumors in children exposed to barbiturates. *J. Natl. Cancer Inst.* 1978;61(4):1031–1034.
109. Goldhaber MK, Selby JV, Hiatt RA, et al. Exposure to barbiturates in utero and during childhood risk of intracranial and spinal cord tumors. *Cancer Res.* 1990;50(15):4600–4603.
110. Gurney JG, Mueller BA, Preston-Martin S, et al. A Study of Pediatric Brain Tumors and Their Association with Epilepsy and Anticonvulsant Use. *Neuroepidemiology*. 1997;16(5):248–255.
111. Howe GR, Burch JD, Chiarelli AM, et al. An exploratory case-control study of brain tumors in children. *Cancer Res.* 1989;49(15):4349–4352.
112. Momen NC, Munk-Olsen T, Li J, et al. Antidepressant use during pregnancy and childhood cancer in the offspring. *Pharmacoepidemiol Drug Saf*. 2018;27(1):114–118.
113. Schneeweiss S, Rassen JA, Brown JS, et al. Graphical Depiction of Longitudinal Study Designs in Health Care Databases. *Ann Intern Med*. 2019;170(6):398.
114. Textor J, Hardt J, Knüppel S. DAGitty: A Graphical Tool for Analyzing Causal Diagrams. *Epidemiology*. 2011;22(5):745.
